# Supplementary material for: Trio-pharmacophore DNA-encoded chemical library for simultaneous selection of fragments and linkers
Source: Nat Commun. 2023 Mar 17;14:1481. doi: 10.1038/s41467-023-37071-1 (PMC10023787; doi:10.1038/s41467-023-37071-1)
Supplement: Supplementary file 1 — Supplementary Information [file 41467_2023_37071_MOESM1_ESM.pdf]

Trio-pharmacophore DNA-encoded Chemical Library for Simultaneous Selection of Fragments and Linkers

Supplementary Methods

All oligonucleotides were purchased from IBA Lifesciences (Göttingen, Germany), Metabion (Oberbayern, Germany), and Eurofins (Ebersberg, Germany) in High-Performance Liquid Chromatography (HPLC)-purified grade, molecular biology grade, Next Generation Sequencing (NGS) grade or on the Controlled Pore Glass (CPG) solid support according to different purposes.

Building blocks of the libraries were purchased from Sigma-Aldrich (St. Louis, USA), Enamine (Kyiv, Ukraine), Alinda Chemical (Moscow, Russia), ChemBridge Corporation (San Diego, USA) and Maybridge Chemical Company (Altrincham, UK).

All chemicals other than building blocks, unless otherwise noted, were purchased from Sigma-Aldrich (St. Louis, USA), Merck Millipore (Darmstadt, Germany), Iris Biotech (Marktredwitz, Germany), Thermo Fisher Scientific (Waltham, USA), Carl Roth (Karlsruhe, Germany), or VWR (Radnor, USA).

All enzymes, unless otherwise noted, were purchased from New England Biolabs (Frankfurt, Germany), or Thermo Fisher Scientific (Waltham, USA).

Bovine trypsin (T8003), bovine carbonic anhydrase II (C7749), and alpha-1-acid glycoprotein from human plasma (G9885) were purchased from Sigma-Aldrich (St. Louis, USA). Human matrix metalloprotease-2 (10082-HNAH) and -9 (10327-HNAH) were purchased from Sino Biological (Beijing, China). Builiding blocks sulfanilamide, compound A, 4-aminomethyl benzamidine, compound B, **66**, **182**, **693**, **787**, **826**, and **828** were purchased from Sigma-Aldrich (St. Louis, USA) and Enamine (Kyiv, Ukraine).

Synthesis of single-sided DNA-compound conjugates

Building blocks were conjugated at 5’-amine-modifed DNA and 3’-amine-modified DNA, resulting in 5’-DNA-compound conjugates and 3’-DNA-compound conjugates, respectively.

Conjugation of building blocks with carboxylic acid to amine-modified DNA on pseudo-solid support.

Anion-exchange resin (diethylaminoethanol, DEAE, GE Healthcare) was used as solid support for immobilizing oligonucleotides. 50 µl of DEAE slurry was loaded on a centrifuge column

(Pierce Spin Columns, Thermo Fisher Scientific) and washed with 500 µl of DEAE binding buffer (10 mM AcOH and 0.005% Triton-X 100) for three times by spinning at 492 g for 1 min and discarding the flow-through. 2 nmol of amine-modified oligonucleotide dissolved in 100 µl of DEAE binding buffer was loaded on the resin and agitated for 15 min on an orbital shaker. Next, binding buffer was discarded after centrifugation. In parallel, 50 mM of small organic molecule with carboxylic acid was activated by EDC-HCl (1 eq), HOAt (1 eq), and DIPEA (5 eq) in 100 µl of DMF: MeOH (3:2) for 30 min at RT. Activated carboxylic acid was added on the resin to react with the amine-modified oligonucleotide. After 1 h, the reaction solution was removed and replaced with fresh-activated carboxylic acid. Coupling was repeated three times to obtain high reaction yield. Next, the reaction solution was removed, and resin was washed with DMF three times and binding buffer three times. The small molecule-DNA conjugate was then eluted with 3 M NaOAc, pH 4.7 by agitating the resin for five minutes. The eluted conjugate was subjected the ethanol precipitation and characterized with UPLC-ESI-MS.

Conjugation of building block with anhydride to amine-modified DNA

2 nmol of amine- modified DNA was dissolved in 0.2 M $NaHCO_3$ buffer to a final volume of 100 µL. Building block was dissolved in DMF to reach 500 mM. 5 µL of building block solution was added to DNA and stirred for 3 h at RT. The reaction crude was then desalted by ethanol precipitation and characterized by UPLC-ESI-MS.

Conjugation of 3-iodophenyl isothiocyanate to amine-modified DNA

2 nmol of amino-modified oligo was dissolved in 100 µL of 0.1 M Na_2CO_3 buffer. 3-Iodophenyl isothiocyanate was dissolved in DMSO to reach 100 mM. 10 µL of compound solution was added to oligo followed by adding 300 µL of DMSO. The reaction was stirred for 3 h at RT. The reaction crude was then desalted by ethanol precipitation and characterized by UPLC-ESI-MS.

HPLC purification and validation by UPLC-ESI-MS

The crudes were re-suspended with 1 ml MilliQ water and purified via reverse-phase HPLC (Waters, USA) on a Clarity 3u Oligo-RT C18 reverse−phase HPLC column (Phenomenex, CA, USA), applying a gradient from 5% ACN/100 mM TEAA to 35% ACN/100 mM TEAA over 30 min. The correct fraction was confirmed via UPLC-ESI-MS, Waters, USA) combined with an analytical ACQUITY UPLC OST C18 column (Waters, USA). The fractions were dried using a Vacuum concentrator. The conjugates dissolved in MilliQ were quantified by measuring the absorbance at 260 nm.

The mass of HPLC purified oligonucleotide-conjugate was measured by liquid chromatography with electrospray ionization mass spectrometry (UPLC-ESI-MS). The reverse-phased ACQUITY UPLC OST C18 column, (Waters, USA) (1.7 µm, 2.1 x 100 mm) was used as stationary phase. The mobile phase was Buffer A (5 mM triethylammonium bicarbonate, TEAB in H2O) and Buffer B (5 mM TEAB in acetonitrile). The electrospray

ionization mass spectrometry (ACQUITY TQ Detector, Waters, Massachusetts, USA) was used in negative ion-mode for DNA mass detection and analysis. The ESI parameters were set as following: cone voltage: 30 V; scan duration: 1 s; mass range: 500–2000 m/z.

Synthesis of DNA-compound-DNA conjugates via DNA-templated reactions

Different types of DNA-templated reactions were implemented between the matching functional groups through hybridization of complementary regions of DNA under reaction conditions. The reaction crude was subjected to ethanol precipitation to desalt. Next, the pellet was dissolved in 1X Urea PAGE Loading Dye and split in 12 fractions and loaded on a 10% denaturing Urea PAGE to monitor the reaction yield and purify the product obtaining the DNA-compound-DNA conjugate.

DNA-templated amine acylation

DNA-templated amine acylation was performed between one 5’-DNA-compound conjugate and one 3’-DNA-compound conjugates displaying a carboxylic acid and an amine, respectively. 500 pmol of 5’-DNA-compound conjugate and 500 pmol of 3’-DNA-compound conjugate were mixed in 100 µl of buffer 1 (1x phosphate buffered saline (PBS), 1 M NaCl, pH 7.5) and allowed to anneal at RT for 20 min. To the solution was added DMTMM at a final concentration of 32 mM. The reaction was stirred at RT overnight.

DNA-templated reductive amination

DNA-templated reductive amination was performed between one 5’-DNA-compound conjugate and one 3’-DNA-compound conjugate displaying an aldehyde and an amine, respectively. 500 pmol of 5’-DNA-compound conjugate and 500 pmol of 3’-DNA-compound conjugate were mixed in 100 µl of buffer 1 and allowed to anneal at RT for 20 min. To the solution was added $NaBH_3CN$ at a final concentration of 50 mM and the reaction was stirred at RT overnight.

DNA-templated Cu(I)-catalyzed azide-alkyne cycloaddition

DNA-templated Cu(I)-catalyzed azide-alkyne cycloaddition was performed between one 5’-DNA-compound conjugate and one 3’-DNA-compound conjugate displaying an azide and an alkyne, respectively. 500 pmol of 5’-DNA-compound conjugate and 500 pmol of 3’-DNA-compound conjugate were mixed in 100 µl of buffer 1 and allowed to anneal at RT for 20 min. $CuSO_4$ was premixed with 10 fold of THPTA to reach 1 mM in buffer 1. 10 µl of $CuSO_4$: THPTA mixture was added to DNA and sodium ascorbate was added to reach 1 mM final concentration. The reaction was stirred for 3 h at RT.

DNA-templated Pd(II)-catalyzed reactions

DNA-templated enone formation was performed between 5’-DNA-compound conjugate and one 3’-DNA-compound conjugate displaying an alkyne and alkene, respectively. DNA-templated transmetallation, insertion, and beta-hydride elimination was performed between 5’-

DNA-compound conjugate and one 3'-DNA-compound conjugate displaying an alkyne or alkene and boronic acid. DNA-templated Heck reaction was performed between 5'-DNA-compound conjugate and one 3'-DNA-compound conjugates displaying an alkene and an aryl iodide. All reactions were catalyzed by Pd (II) (Na_2PdCl_4) according to a previously reported protocol[1]. 500 pmol of 5'-DNA-compound conjugate and 500 pmol of 3'-DNA-compound conjugates were mixed in 100 µl of buffer 1 and allowed to anneal at RT for 20 min. The reactions were initiated by adding 5 µl of Na_2PdCl_4. Reaction was stirred at 37 °C for 1 h, before being quenched by 1 µl 1 M 2-mercaptoethanol.

DNA-templated Diels-Alder cycloaddition

DNA-templated Diels-Alder cycloaddition was performed between one 5'-DNA-compound conjugate and one 3'-DNA-compound conjugates displaying a maleimide and a diene, respectively. 500 pmol of 5'-DNA-compound conjugate and 500 pmol of 3'-DNA-compound conjugates were mixed in 100 µl of buffer 2 (3 M NaOAc, pH 4.7) and allowed to react overnight at RT.

DNA-templated copper-free click reaction

DNA-templated copper-free click reaction was performed between one 5'-DNA-compound conjugate and one 3'-DNA-compound conjugate displaying an azide and a DBCO, respectively. 500 pmol of 5'-DNA-compound conjugate and 500 pmol of 3'-DNA-compound conjugates were mixed in 100 µl of buffer 1 and allowed to react overnight at RT.

DNA-templated photochemical reaction

DNA-templated photochemical reaction was performed between one 5'-DNA-compound conjugate and one 3'-DNA-compound conjugate displaying an aryl azide and an amine, respectively. 500 pmol of 5'-DNA-compound conjugate and 500 pmol of 3'-DNA-compound conjugate were mixed in 50 µl of buffer 1 and allowed to anneal at RT for 20 min. The mixture was then irradiated by UV lamp (254 nm) on ice for 15 min.

DNA-templated Michael addition

DNA-templated Michael addition was performed between one 5'-DNA-compound conjugate and one 3'-DNA-compound conjugate displaying a maleimide / alkene and a thiol / amino group, respectively. 500 pmol of 5'-DNA-compound conjugate and 500 pmol of 3'-DNA-compound conjugate were mixed in 100 µl of buffer 1 and allowed to react overnight at RT.

Off-DNA hit resynthesis of C-0, C-1, C-2, C-3, C-4, and C-5.

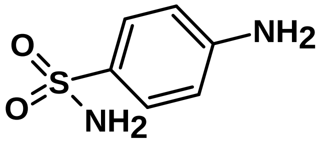

Sulfanilamide

1H NMR (600 MHz, DMSO-d6) δ 7.60 – 7.55 (m, 2H), 7.14 (s, 1H), 6.67 – 6.62 (m, 2H), 5.68 (d, J = 5.9 Hz, 1H), 5.63 (d, J = 5.6 Hz, 1H). 13C NMR (150 MHz, DMSO-d6) δ 151.98, 132.06, 127.56, 113.12. MW: 172.20492, observed MW: 172

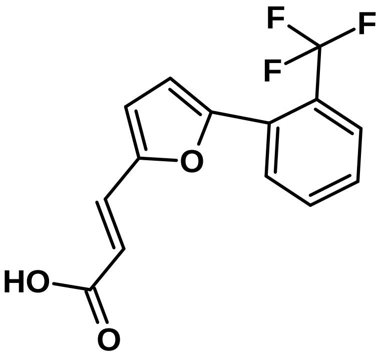

Compound A

1H NMR (600 MHz, DMSO-d6) δ 12.09 (s, 1H), 7.76 (dd, J = 8.4, 1.2 Hz, 1H), 7.65 (ddd, J = 10.3, 6.5, 1.3 Hz, 1H), 7.58 (dd, J = 10.4, 1.6 Hz, 1H), 7.43 – 7.36 (m, 2H), 7.18 (d, J = 5.6 Hz, 1H), 7.11 (d, J = 5.3 Hz, 1H), 6.34 (d, J = 16.8 Hz, 1H). 13C NMR (150 MHz, DMSO-d6) δ 168.43, 153.71, 153.69, 151.95, 129.38, 128.91, 128.03, 127.78, 127.19, 126.13, 122.93, 116.83, 114.62.MW: 282.21467, observed MW:282

Synthesis of (2*E*)-*N*-(2-aminoethyl)-3-{5-[2-(trifluoromethyl) phenyl] furan-2-yl} prop-2-enamide

Compound A ((2*E*)-3-{5-[2-(trifluoromethyl) phenyl] furan-2-yl} prop-2-enoic acid) was conjugated to ethylenediamine to display an amine functionality for the synthesis of C-1–C-5.

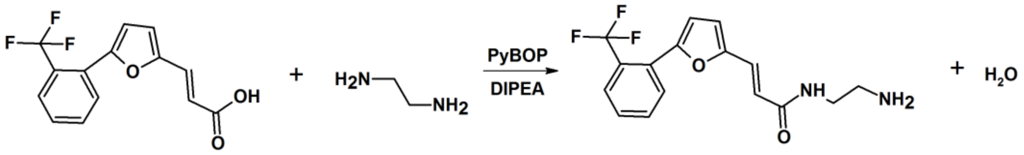

(2*E*)-3-{5-[2-(trifluoromethyl)phenyl]furan-2-yl}prop-2-enoic acid (2*E*)-*N*-(2-aminoethyl)-3-{5-[2-(trifluoromethyl)phenyl]furan-2-yl}prop-2-enamide

Compound A

50 mg of compound A was dissolved in DMF (50 mM) and benzotriazol-1-yl-oxytripyrrolidinophosphonium hexafluorophosphate (PyBOP) (1 eq, 92 mg) was added to

activate for 20 min under argon atmosphere. Activated compound A was added drop by drop to ethylenediamine (5 eq, 53 mg) while stirring. The reaction was stirred under argon protection for 18 h at RT. Small amount of reaction crude (2 µmol) was injected in UPLC-ESI-MS to evaluate the reaction yield. When the reaction product was detected, two-fold volume of water was added to the reaction and the crude was freeze-dried. The dried reaction crude was then dissolved in 500 µL ACN/H_2O (1:1) and was purified by reverse-phase HPLC to obtain the reaction product. HPLC fractions were injected in UPLC-ESI-MS to identify the best fraction, and the desired fractions were freeze-dried yielding 43 mg (75%).

Synthesis of C-0

17 mg of compound A was dissolved in 500 µL of DMF and added to 1 eq (31 mg) to PyBOP in 500 µL of DMF for activation under argon protection for 20 min at RT. 12 mg of sulfanilamide was dissolved in 500 µL of DMF and pH was tuned to 9 by DIPEA. To the activated compound A was added sulfanilamide solution and the mixture was allowed to react under argon protection for 18 h at RT. The product was confirmed by UPLC-ESI-MS, purified from reverse-phase HPLC and dried to obtain 15 mg (57%) C-0. 1H NMR (600 MHz, DMSO-d6) δ 10.10 (s, 1H), 7.79 (s, 3H), 7.76 (dd, J = 8.4, 1.2 Hz, 1H), 7.63 (ddd, J = 10.3, 6.5, 1.2 Hz, 1H), 7.58 (dd, J = 10.3, 1.6 Hz, 1H), 7.40 (ddd, J = 8.2, 6.5, 1.6 Hz, 1H), 7.36 (d, J = 16.2 Hz, 1H), 7.18 (d, J = 5.6 Hz, 1H), 7.15 – 7.11 (m, 3H), 6.67 (d, J = 16.2 Hz, 1H).

Synthesis of C-1

4-[(4-sulfamoylanilino) methyl] benzoic acid was synthesized by reductive amination between sulfanilamide and 4-formyl benzoic acid with the following procedure. 20 mg of sulfanilamide and 1 eq (17.5 mg) of formyl benzoic acid were dissolved in methanol (50 mM) and stirred for 30 min at RT. To the solution 5 eq (36.7 mg) of $NaBH_3CN$ was added as powder and the reaction was stirred for 18 h under argon protection. After the overnight reaction, white precipitant was observed as the product. The precipitant was filtered, dissolved in water, and purified by reverse-phase HPLC for the reaction with (2E)-N-(2-aminoethyl)-3-{5-[2-(trifluoromethyl) phenyl] furan-2-yl} prop-2-enamide (17 mg, 60%).

8 mg of 4-[(4-sulfamoylanilino) methyl] benzoic acid obtained from the above reaction was dissolved in 200 µL of DMF and activated by 1 eq (13 mg) PyBOP for 20 min under argon protection at RT. To the activated mixture was added 8.4 mg of (2E)-N-(2-aminoethyl)-3-{5-[2-(trifluoromethyl) phenyl] furan-2-yl} prop-2-enamide in 100 µL of DMF, whose pH was tuned in advance by adding 5 eq of DIPEA. The reaction was stirred under argon protection for 18 h at RT. Reaction crude was purified by reverse-phase HPLC to obtain C-1 (7.7 mg, 48%). 1H NMR (600 MHz, DMSO-d6) δ 8.34 (t, J = 5.2 Hz, 1H), 7.86 – 7.81 (m, 2H), 7.76 (dd, J = 8.4, 1.2 Hz, 1H), 7.68 – 7.62 (m, 3H), 7.58 (dd, J = 10.4, 1.6 Hz, 1H), 7.50 (t, J = 5.1 Hz, 1H), 7.42 (d, J = 1.2 Hz, 1H), 7.43 – 7.37 (m, 2H), 7.31 (d, J = 16.1 Hz, 1H), 7.17 (d, J = 5.6 Hz, 1H),

7.15 – 7.11 (m, 3H), 6.83 – 6.76 (m, 2H), 6.62 (t, J = 5.4 Hz, 1H), 6.50 (d, J = 16.5 Hz, 1H), 4.36 (dt, J = 5.2, 0.9 Hz, 2H), 3.47 (q, J = 4.8 Hz, 2H), 3.41 (dd, J = 5.2, 4.2 Hz, 2H).

Synthesis of C-2

30 mg of catechol-*O*,*O*-diacetic acid was dissolved in 500 µL of DMF. To the solution of catechol-*O,O*-diacetic acid, 1 eq (50 mg) of 1-[Bis (dimethylamino) methylene]-1*H*-1,2,3-triazolo [4,5-*b*] pyridinium 3-oxide hexafluorophosphate (HATU) and 1 eq (18 mg) of 1-Hydroxy-7-azabenzotriazole (HOAt) was added to activate the carboxylic acid. After 20 min activation under argon protection at RT, 23 mg of sulfanilamide in 500 µL of DMF pre-mixed with 5 eq (86 mg, 116 µl) of DIPEA was added to the activated carboxylic acid drop by drop. The reaction was stirred under argon protection for 18 h at RT. The resulting product {2-[2-oxo-2-(4-sulfamoylanilino) ethoxy] phenoxy} acetic acid was purified by reverse-phase HPLC and dried under vacuum.

7.5 mg of {2-[2-oxo-2-(4-sulfamoylanilino) ethoxy] phenoxy} acetic acid was dissolved 100 µL of DMF and activated by 1 eq of PyBOP under argon protection for 20 min at RT. To the activated carboxylic acid was added 6.4 mg of (2*E*)-*N*-(2-aminoethyl)-3-{5-[2-(trifluoromethyl) phenyl] furan-2-yl} prop-2-enamide in 100 µL of DMF. The reaction was stirred under argon protection for 18 h at RT. Reaction crude was purified by reverse-phase HPLC to afford C-2 (8 mg, 60%). 1H NMR (600 MHz, DMSO-d6) δ 9.98 (s, 1H), 7.94 (t, J = 4.9 Hz, 1H), 7.79 – 7.71 (m, 5H), 7.65 (ddd, J = 10.3, 6.5, 1.2 Hz, 1H), 7.58 (dd, J = 10.4, 1.6 Hz, 1H), 7.52 (t, J = 5.2 Hz, 1H), 7.39 (ddd, J = 8.2, 6.5, 1.6 Hz, 1H), 7.33 (d, J = 16.1 Hz, 1H), 7.18 (d, J = 5.6 Hz, 1H), 7.15 – 7.11 (m, 3H), 6.94 – 6.85 (m, 4H), 6.48 (d, J = 16.5 Hz, 1H), 4.72 (s, 1H), 4.48 (s, 1H), 3.34 (dt, J = 5.0, 3.9 Hz, 2H), 3.33 – 3.28 (m, 2H).

Synthesis of C-3

60 mg of sulfanilamide was dissolved in 500 µL of DMF and the pH was tuned to 9 by DIPEA. To the solution of sulfanilamide, glutaric anhydride (1 eq, 40 mg) dissolved in 200 µL of DMF was added drop by drop and the reaction was stirred for 18 h at RT. Reaction crude was purified by reverse-phase HPLC to afford 5-oxo-5-(4-sulfamoylanilino)pentanoic acid.

7 mg of 5-oxo-5-(4-sulfamoylanilino) pentanoic acid was dissolved in 100 µL of DMF and was activated by 1 eq HATU and 1 eq HOAt in 100 µL of DMF for 20 min at RT under argon protection. 9 mg of (2E)-N-(2-aminoethyl)-3-{5-[2-(trifluoromethyl) phenyl] furan-2-yl} prop-2-enamide was dissolved in 50 µL of DMF, mixed with 5 eq DIPEA and added to activated carboxylic acid. The reaction was stirred under argon protection for 18 h at RT and purified by reverse-phase HPLC to obtain C-3 (9.4 mg, 65%). 1H NMR (600 MHz, DMSO-d6) δ 9.73 (s, 1H), 7.79 – 7.74 (m, 3H), 7.73 – 7.68 (m, 2H), 7.65 (ddd, J = 10.3, 6.5, 1.2 Hz, 1H), 7.61 – 7.54 (m, 2H), 7.52 (s, 1H), 7.52 (d, J = 10.3 Hz, 1H), 7.40 (ddd, J = 8.3, 6.5, 1.6 Hz, 1H), 7.31 (d, J = 16.1 Hz, 1H), 7.18 (d, J = 5.6 Hz, 1H), 7.15 – 7.11 (m, 3H), 6.49 (d, J = 16.5 Hz, 1H),

3.36 – 3.31 (m, 2H), 3.30 – 3.24 (m, 2H), 2.37 (t, J = 8.4 Hz, 2H), 2.26 (t, J = 8.6 Hz, 2H), 1.83 (p, J = 8.7 Hz, 2H).

Synthesis of C-4

C-4 was obtained by Cu (I)-catalyzed azide alkyne cycloaddition between 4-azido-N-(4-sulfamoylphenyl) benzamide and N-(2-{[(2E)-3-{5-[2-(trifluoromethyl)phenyl]furan-2-yl}prop-2-enoyl]amino}ethyl)but-3-ynamide. 4-azido-N-(4-sulfamoylphenyl) benzamide was obtained by conjugating sulfanilamide to 4-azido benzoic acid.

16.3 mg of 4-azido benzoic acid was dissolved in 500 μL of DMF and activated with 1 eq (52 mg) PyBOP under argon protection for 20 min at RT. 26 mg of sulfanilamide in 200 μL of DMF was tuned to pH 9 by DIPEA and was added to activated carboxylic acid and the mixture was stirred under argon protection for 18 h at RT in darkness. The reaction crude was purified by reverse-phase HPLC to obtain 4-azido-N-(4-sulfamoylphenyl) benzamide.

N-(2-{[(2*E*)-3-{5-[2-(trifluoromethyl)phenyl]furan-2-yl}prop-2-enoyl]amino}ethyl)but-3-ynamide was obtained by conjugating pentynoic acid to (2*E*)-*N*-(2-aminoethyl)-3-{5-[2-(trifluoromethyl)phenyl]furan-2-yl} prop-2-enamide. 2 eq of pentynoic acid was activated by 2 eq PyBOP in 200 μL of DMF under argon protection for 20 min at RT. 7 mg (1 eq) of (2E)-N-(2-aminoethyl)-3-{5-[2-(trifluoromethyl) phenyl] furan-2-yl} prop-2-enamide was mixed with DIPEA to tune the pH to 9, and was added to the activated pentynoic acid. The reaction was stirred under argon protection for 18 h at RT and the crude was purified by reverse-phase HPLC to obtain the product.

4.5 mg of 4-azido-*N*-(4-sulfamoylphenyl) benzamide and 6 mg of N-(2-{[(2E)-3-{5-[2-(trifluoromethyl) phenyl] furan-2-yl} prop-2-enoyl] amino} ethyl) but-3-ynamide were dissolved and mixed in 500 μL of DMF. 0.3 eq of $CuSO_4$ and 0.6 eq of THPTA were mixed in 100 μL of 1x PBS pH 7.4 and the mixture was added to the reaction partner. 1.5 eq of sodium ascorbate dissolved in 1x PBS was added to trigger the reaction by reducing Cu^{++} to Cu^{+}. The reaction was stirred under argon protection for 18 h at RT and the crude was purified by reverse-phase HPLC to obtain C-4 (2.3 mg, 13%). 1H NMR (600 MHz, DMSO-d6) δ 9.81 (s, 1H), 8.06 – 8.02 (m, 4H), 7.91 – 7.86 (m, 4H), 7.83 (s, 1H), 7.81 – 7.73 (m, 11H), 7.68 – 7.62 (m, 4H), 7.58 (dd, J = 10.4, 1.6 Hz, 2H), 7.52 (s, 1H), 7.51 (d, J = 10.3 Hz, 1H), 7.40 (ddd, J = 8.2, 6.5, 1.6 Hz, 2H), 7.31 (d, J = 16.1 Hz, 2H), 7.19 (d, J = 5.6 Hz, 2H), 7.15 – 7.11 (m, 6H), 6.50 (d, J = 16.5 Hz, 1H), 3.34 (dt, J = 5.3, 4.0 Hz, 4H), 3.30 – 3.25 (m, 4H), 2.96 (t, J = 8.2 Hz, 4H), 2.62 (t, J = 8.3 Hz, 4H).

Synthesis of C-5

25 mg of diglycolic anhydride was dissolved in 200 μL of DMF. 37 mg of sulfanilamide was dissolved in 200 μL of DMF and pH was tuned to 9 by DIPEA. Sulfanilamide was added to diglycolic anhydride drop by drop and the reaction was stirred under argon protection for 18 h

at RT. The crude was purified by reverse-phase HPLC to obtain [2-oxo-2-(4-sulfamoylanilino) ethoxy] acetic acid.

7.2 mg of [2-oxo-2-(4-sulfamoylanilino)ethoxy]acetic acid was dissolved in 100 µL of DMF and was activated by 1 eq (15.5 mg) PyBOP for 20 min at RT under argon protection. To the activated carboxylic acid 8 mg of (2*E*)-*N*-(2-aminoethyl)-3-{5-[2-(trifluoromethyl) phenyl] furan-2-yl} prop-2-enamide was added in 100 µL of DMF, pH 9. The reaction was stirred under argon protection for 18 h at RT the crude was purified by reverse-phase HPLC to obtain C-5 (10 mg, 58%). 1H NMR (600 MHz, DMSO-d6) δ 9.81 (s, 1H), 8.06 – 8.01 (m, 4H), 7.91 – 7.86 (m, 4H), 7.83 (s, 1H), 7.81 – 7.73 (m, 11H), 7.68 – 7.62 (m, 4H), 7.55 (dd, J = 10.4, 1.6 Hz, 2H), 7.52 (s, 1H), 7.50 (d, J = 10.3 Hz, 1H), 7.41 (ddd, J = 8.2, 6.5, 1.6 Hz, 2H), 7.31 (d, J = 16.1 Hz, 2H), 7.18 (d, J = 5.6 Hz, 2H), 7.15 – 7.11 (m, 6H), 6.50 (d, J = 16.5 Hz, 1H), 3.33 (dt, J = 5.3, 4.0 Hz, 4H), 3.30 – 3.25 (m, 4H), 2.96 (t, J = 8.2 Hz, 4H), 2.63 (t, J = 8.3 Hz, 4H).

Off-DNA hit resynthesis of T-0, T-1, T-2, T-3, T-4 and T-5

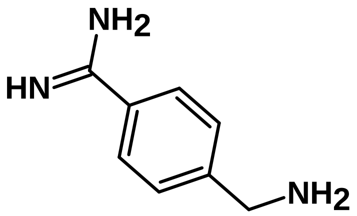

4-aminomethyl benzamidine

1H NMR (600 MHz, DMSO-d6) δ 8.02 (s, 1H), 7.66 – 7.61 (m, 2H), 7.24 (dt, J = 8.1, 1.1 Hz, 2H), 6.70 (s, 1H), 4.06 – 3.96 (m, 2H), 3.88 (dt, J = 7.2, 6.2 Hz, 1H).13C NMR (150 MHz, DMSO-d6) δ 164.01, 142.78, 130.16, 127.48, 126.86, 45.91. MW: 149.19304, observed MW: 149

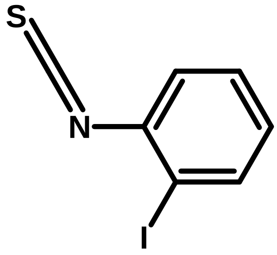

Compound B

1H NMR (600 MHz, DMSO-d6) δ 7.63 (dd, J = 6.8, 1.1 Hz, 1H), 7.24 (ddd, J = 6.9, 5.9, 1.1 Hz, 1H), 7.22 (dd, J = 7.3, 1.7 Hz, 1H), 7.01 – 6.95 (m, 1H). 13C NMR (150 MHz, DMSO-d6) δ 138.71, 138.32, 136.05, 128.96, 127.56, 126.79, 94.25. MW: 261.08283, observed MW: 261

Synthesis of *N*-(3-iodophenyl) thiourea

Compound B (1-iodo-3-isothiocyanatobenzene) was conjugated to ethylenediamine to display an amine functionality for the synthesis of T-1 to T-5.

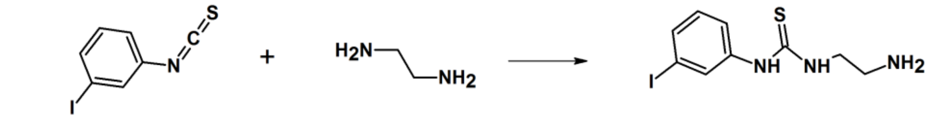

1-iodo-3-isothiocyanatobenzene (50 mg) was dissolved in 2 ml DMSO, added to ethylenediamine (5 eq, 57.5 mg) in 1 ml DMSO. The reaction was stirred under argon protection for 18 h at RT. Small amount of reaction crude (2 μmol) was injected in UPLC-ESI-MS to evaluate the reaction yield. When the reaction product was detected, two-fold volume of water was added to the reaction and the crude was freeze-dried. The resulting crude was purified by reverse-phase HPLC to obtain the desired N-(3-iodophenyl) thiourea (38 mg, 65%).

Synthesis of T-0

22 mg of 4-aminomethyl benzamidine was dissolved in 300 μL of DMSO and pH was tuned to 8-9 by Et_3N. 33.1 mg of compound B was dissolved in 200 μL of DMSO and was mixed with 4-aminomethyl benzamidine. The reaction was allowed to stir under argon protection for 16 h at RT. The reaction mixture was purified by reverse-phase HPLC to obtain T-0 (36 mg, 61%). 1H NMR (600 MHz, DMSO-d6) δ 9.47 (s, 1H), 8.04 – 8.00 (m, 2H), 7.99 (t, J = 2.1 Hz, 1H), 7.67 – 7.62 (m, 2H), 7.54 (ddd, J = 7.2, 2.2, 1.1 Hz, 1H), 7.50 (ddd, J = 7.8, 1.9, 0.9 Hz, 1H), 7.25 (dt, J = 8.4, 1.1 Hz, 2H), 7.12 (t, J = 7.5 Hz, 1H), 6.69 (s, 1H), 4.77 (dt, J = 5.0, 1.0 Hz, 2H).

Synthesis of T-1

39 mg of 4-aminomethyl benzamidine was dissolved in 1.5 ml of DMSO and the pH was tuned to 9 by DIPEA. To the solution of sulfanilamide, glutaric anhydride (1 eq) dissolved in 500 μL of DMSO was added drop by drop and the reaction was stirred for 18 h at RT. Reaction crude was analyzed by UPLC-ESI-MS and the resulting product 5-{[(4-carbamimidoylphenyl)methyl]amino}-5-oxopentanoic acid was used after drying without purification.

8 mg of 5-{[(4-carbamimidoylphenyl) methyl] amino}-5-oxopentanoic acid was dissolved in 100 μL of DMSO and activated by 1 eq (15.8 mg) of PyBOP for 20 min under argon protection at RT. 1 eq of N-(3-iodophenyl) thiourea dissolved in 100 μL of DMSO was added to the activated carboxylic acid and the reaction was allowed to stir under argon protection for 16 h at RT. The

reaction crude was purified by reverse-phase HPLC to obtain T-1 (10.7 mg, 62%). 1H NMR (600 MHz, DMSO-d6) δ 8.03 (s, 0H), 7.99 (t, J = 2.1 Hz, 0H), 7.90 (t, J = 4.5 Hz, 0H), 7.67 – 7.62 (m, 1H), 7.57 – 7.51 (m, 1H), 7.48 (ddd, J = 7.8, 1.9, 0.9 Hz, 0H), 7.24 (dt, J = 8.4, 1.1 Hz, 1H), 7.13 (t, J = 7.5 Hz, 0H), 6.70 (s, 1H), 4.35 (dt, J = 5.6, 1.0 Hz, 1H), 3.68(q, J = 4.5 Hz, 1H), 2.28 (dt, J = 16.7, 8.5 Hz, 2H), 1.77 (p, J = 8.6 Hz, 1H).

Synthesis of T-2

33 mg of 4-formyl benzoic acid in 500 µL methanol was mixed with 49 mg of 4-aminomethyl benzamidine in 3.2 ml of methanol at RT. 69 mg (5 eq) of $NaBH_3CN$ was added to the mixture and the reaction was allowed to take place under argon protection for 18 h at RT. The product 4-({[(4-carbamimidoylphenyl) methyl] amino} methyl) benzoic acid was precipitated from the mixture as white pellet and was used after filtering without purification.

6 mg of 4-({[(4-carbamimidoylphenyl) methyl] amino} methyl) benzoic acid was dissolved in 100 µL of water and was activated by 1 eq of EDC and 1 eq of NHS for 30 min at RT. 1 eq of N-(3-iodophenyl) thiourea dissolved in 350 µL of DMSO was added to the activated carboxylic acid and the reaction was stirred under argon protection for 16 h at RT. The crude was purified by reverse-phase HPLC to obtain T-2 (6.6 mg, 56%). 1H NMR (600 MHz, DMSO-d6) δ 8.03 – 7.98 (m, 1H), 7.85 – 7.81 (m, 1H), 7.68 – 7.61 (m, 2H), 7.57 – 7.48 (m, 1H), 7.39 (dd, J = 8.2, 1.3 Hz, 1H), 7.23 (dt, J = 8.8, 1.3 Hz, 1H), 7.13 (t, J = 7.5 Hz, 1H), 6.70 (s, 1H), 4.02 – 3.98 (m, 2H), 3.71 (q, J = 4.6 Hz, 1H), 3.53 (t, J = 4.8 Hz, 1H).

Synthesis of T-3

26 mg of suberic acid dissolved in 500 µL of DMSO was activated with 1 eq (56.7 mg) HATU and 1 eq (20.3 mg) of HOAt for 30 min at RT. 36 mg of 4-aminomethyl benzamidine in 1.8 ml of DMSO, pH 8 was added to the activated suberic acid and the reaction was stirred stir under argon protection for 18 h at RT. The reaction product was purified from reverse-phase HPLC to obtain 8-{[(4-carbamimidoylphenyl) methyl] amino}-8-oxooctanoic acid.

7.2 mg of 8-{[(4-carbamimidoylphenyl) methyl] amino}-8-oxooctanoic acid was dissolved in 200 µL of DMSO and was activated with 1 eq of PyBOP for 20 min at RT. 1 eq of N-(3-iodophenyl) thiourea dissolved in 100 µL of DMSO, pH 9 was added to the activated carboxylic acid and the reaction was allowed to stir under argon protection for 16 h at RT. The reaction product was purified from the crude by reverse-phase HPLC to obtain T-3 (6.6 mg, 46%). 1H NMR (600 MHz, DMSO-d6) δ 9.40 (s, 1H), 8.03 (t, J = 5.6 Hz, 1H), 8.02 (s, 1H), 7.99 (t, J = 2.1 Hz, 1H), 7.93 (t, J = 4.5 Hz, 1H), 7.67 – 7.62 (m, 2H), 7.59 (t, J = 5.1 Hz, 1H), 7.54 (ddd, J = 7.2, 2.2, 1.1 Hz, 1H), 7.50 (ddd, J = 7.8, 1.9, 0.9 Hz, 1H), 7.23 (dt, J = 8.4, 1.1 Hz, 2H), 7.13 (t, J = 7.5 Hz, 1H), 6.70 (s, 1H), 4.35 (dt, J = 5.6, 0.9 Hz, 2H), 3.66 (q, J = 4.5 Hz, 2H), 3.37 – 3.32 (m, 2H), 2.21 (t, J = 8.5 Hz, 2H), 2.16 (t, J = 8.5 Hz, 2H), 1.56 (pd, J = 8.5, 8.0, 0.9 Hz, 4H), 1.39 – 1.30 (m, 4H).

Synthesis of T-4

29 mg of 1, 4 cyclohexane dicarboxylic acid was dissolved in 500 µL of methanol and was activated by 1 eq (32 mg) EDC and 1 eq (19.4 mg) NHS for 30 min at RT. 37 mg of 4-aminomethyl benzamidine was dissolved in 1.7 ml of methanol and pH was tuned to 8 by DIPEA. To the activated carboxylic acid, 4-aminomethyl benzamidine was added and the reaction was stirred under argon protection for 16 h at RT. The reaction crude was purified by reverse-phase HPLC to obtain 4-{[(4-carbamimidoylphenyl) methyl] carbamoyl} cyclohexane-1-carboxylic acid.

8 mg of 4-{[(4-carbamimidoylphenyl) methyl] carbamoyl} cyclohexane-1-carboxylic acid in 200 µL of DMSO was activated by 1 eq (13.7 mg) PyBOP for 20 min at RT. To the solution, 1 eq of N-(3-iodophenyl) thiourea dissolved in 100 µL of DMSO pH 9 was added. The reaction was stirred under argon protection for 18 h at RT. The reaction crude was purified by reverse-phase HPLC to obtain T-4 (6.3 mg, 40%). 1H NMR (600 MHz, DMSO-d6) δ 8.03 – 7.98 (m, 1H), 7.95 – 7.86 (m, 1H), 7.67 – 7.62 (m, 1H), 7.57 – 7.47 (m, 1H), 7.25 (dt, J = 8.4, 1.1 Hz, 1H), 7.13 (t, J = 7.5 Hz, 0H), 6.70 (s, 1H), 4.35 (dt, J = 5.8, 0.9 Hz, 1H), 3.68 (q, J = 4.6 Hz, 1H), 3.34 (dt, J = 5.3, 4.6 Hz, 1H), 2.37 – 2.26 (m, 1H), 1.85 – 1.73 (m, 2H), 1.74 – 1.63 (m, 2H).

Synthesis of T-5

10 mg of succinimidyl 4-(N-maleimidomethyl) cyclohexane-1-carboxylate (SMCC) was mixed with 1 eq (9.1 mg) N-(3-iodophenyl) thiourea in 300 µL of DMSO to form an amide bond. The reaction was stirred for 18 h at RT and the reaction product was purified from the crude by reverse-phase HPLC. HPLC fractions were combined by dissolving in 200 µL of DMSO and 3 mg of 4-aminomethyl benzamidine in DMSO, pH 8 was added. The reaction was stirred for 18 h at RT. The reaction crude was purified by reverse-phase HPLC to obtain T-5 (2.7 mg, 23%). 1H NMR (600 MHz, DMSO-d6) δ 9.40 (s, 1H), 8.03 – 7.98 (m, 2H), 7.93 (t, J = 4.5 Hz, 1H), 7.66 – 7.61 (m, 2H), 7.54 (ddd, J = 7.2, 2.2, 1.1 Hz, 1H), 7.52 – 7.48 (m, 2H), 7.22 (dt, J = 8.4, 1.0 Hz, 2H), 7.13 (t, J = 7.5 Hz, 1H), 6.70 (s, 1H), 3.95 – 3.85 (m, 3H), 3.82 (dd, J = 11.7, 5.4 Hz, 1H), 3.74 (dd, J = 11.7, 5.5 Hz, 1H), 3.68 (q, J = 4.6 Hz, 2H), 3.36 – 3.31 (m, 2H), 2.93 (dt, J = 7.2, 5.9 Hz, 1H), 2.80 (dd, J = 12.5, 4.1 Hz, 1H), 2.56 (dd, J = 12.5, 4.0 Hz, 1H), 2.29 (p, J = 6.2 Hz, 1H), 2.04 (pt, J = 6.5, 5.5 Hz, 1H), 1.89 – 1.77 (m, 2H), 1.68 – 1.56 (m, 4H), 1.41 – 1.31 (m, 2H).

Off-DNA synthesis of hit compounds from MMP selections

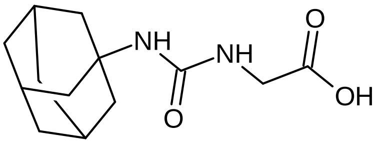

66

1H NMR (600 MHz, DMSO-d6) δ 12.41 (s, 1H), 6.36 (t, J = 5.8 Hz, 1H), 5.61 (s, 1H), 3.86 (d, J = 5.9 Hz, 2H), 2.05 (dq, J = 10.5, 5.3 Hz, 3H), 1.95 (d, J = 5.2 Hz, 6H), 1.65 (t, J = 5.4 Hz, 6H). 13C NMR (150 MHz, DMSO-d6) δ 172.40, 157.12, 50.92, 41.64, 35.90, 29.38. MW:252.3095, measured MW:252

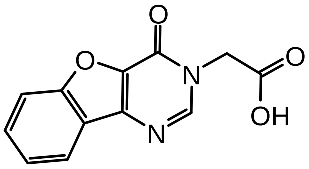

182

1H NMR (600 MHz, DMSO-d6) δ 12.83 (s, 1H), 9.04 (s, 1H), 8.10 (dd, J = 8.7, 1.2 Hz, 2H), 7.66 – 7.62 (m, 2H), 7.55 (ddd, J = 8.7, 6.7, 1.2 Hz, 2H), 7.44 (td, J = 8.7, 1.4 Hz, 2H). 13C NMR (150 MHz, DMSO-d6) δ 170.10, 153.92, 151.07, 149.53, 141.08, 137.59, 131.60, 124.55, 123.89, 117.01, 114.55, 46.80. MW: 244.20292, measured MW: 244

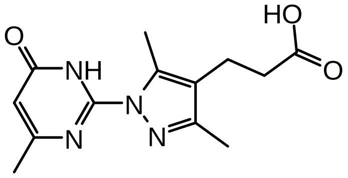

693

1H NMR (600 MHz, DMSO-d6) δ 10.97 (s, 1H), 10.54 (s, 1H), 5.82 (q, J = 1.2 Hz, 1H), 2.80 (t, J = 8.5 Hz, 2H), 2.56 – 2.43 (m, 6H), 2.17 (s, 2H). 13C NMR (150 MHz, DMSO-d6) δ 175.87, 163.94, 162.38, 150.56, 148.04, 134.36, 120.60, 105.06, 33.95, 22.26, 20.08, 12.49, 11.92. MW: 276.29114, observed MW: 276

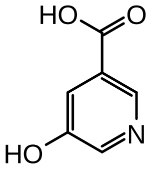

828

1H NMR (600 MHz, DMSO-d6) δ 12.86 (s, 1H), 10.48 (s, 1H), 8.77 (t, J = 1.7 Hz, 2H), 8.32 (t, J = 1.9 Hz, 2H), 7.58 (t, J = 2.0 Hz, 2H). 13C NMR (150 MHz, DMSO-d6) δ 166.94, 154.49, 143.02, 140.81, 127.67, 118.39. MW: 139.1088, observed MW:139

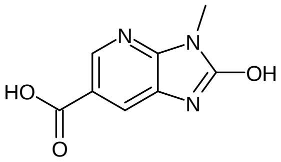

826

1H NMR (600 MHz, DMSO-d6) δ 12.91 (s, 2H), 10.33 (s, 2H), 8.82 (d, J = 1.9 Hz, 3H), 8.16 (d, J = 1.9 Hz, 3H). 13C NMR (150 MHz, DMSO-d6) δ 166.68, 157.24, 150.40, 147.02, 131.51, 125.32, 120.39, 25.70. MW: 193.15948, observed MW: 193

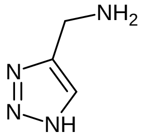

787

1H NMR (600 MHz, DMSO-d6) δ 12.33 (d, J = 1.5 Hz, 1H), 8.03 (d, J = 1.9 Hz, 1H), 4.28 (d, J = 13.4 Hz, 1H), 3.95 (dt, J = 8.4, 6.6 Hz, 1H), 3.86 (dt, J = 8.7, 6.6 Hz, 1H). 13C NMR (150 MHz, DMSO-d6) δ 137.58, 126.08, 35.57. MW: 98.10654, observed MW: 98

Synthesis of fragment pairs linked by linker 1.

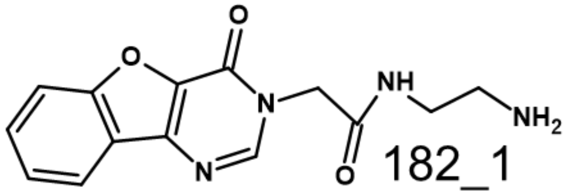

Synthesis of 182_1

200 mg of **182** was dissolved in 2ml DMF and PyBOP (1 eq, 426 mg) in 1 ml DMF was added to activate for 20 min under argon atmosphere. Activated **182** was added drop by drop to ethylenediamine (5 eq, 246 mg, 273 µl in 1 ml DMF) while stirring. The reaction was stirred under argon protection for 3 h at RT. A small amount of reaction crude (2 µmol) was injected in UPLC-ESI-MS to evaluate the reaction yield. When the reaction product was detected, a two-fold volume of water was added to the reaction, and the crude was freeze-dried. The dried

reaction crude was then dissolved in 2 ml DMSO and was purified by reverse-phase HPLC to obtain the reaction product. HPLC fractions were injected in UPLC-ESI-MS to identify the best fraction, and the desired fractions were freeze-dried yielding 212 mg of **182_1** (90%).

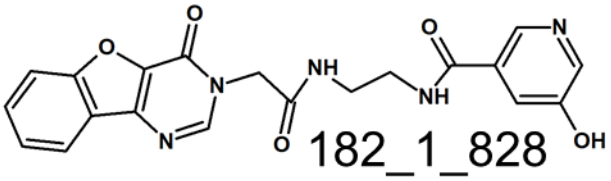

182_1_828

Synthesis of 182_1_828

5 mg of **828** was dissolved in 0.6 ml of DMF and was activated by HATU (1 eq, 13.6 mg in 100 µl DMF) and HOAt (1 eq, 4.9 mg in 100 µl DMF) under argon protection for 20 min at RT. To the solution, 1 eq of **182_1** (10 mg in 0.2 ml of DMF) dissolved with 5 eq of DIPEA (23.22 mg, 31.3 µl) was added. The reaction was stirred for 3 h at RT. A small amount of reaction crude (2 µmol) was injected in UPLC-ESI-MS to evaluate the reaction yield. When the reaction product was detected, a two-fold volume of water was added to the reaction and the crude was freeze-dried. The dried reaction crude was then dissolved in 0.2 ml DMSO and was purified by reverse-phase HPLC to obtain the reaction product. HPLC fractions were injected in UPLC-ESI-MS to identify the best fraction, and the desired fractions were freeze-dried yielding 3.8 mg (26%) of **182_1_828**. 1H NMR (600 MHz, DMSO-d6) δ 10.41 (s, 1H), 9.00 (s, 1H), 8.68 (t, J = 1.9 Hz, 1H), 8.52 (t, J = 5.3 Hz, 1H), 8.33 (t, J = 1.9 Hz, 1H), 8.10 (dd, J = 8.6, 1.3 Hz, 1H), 7.94 (t, J = 5.1 Hz, 1H), 7.64 (td, J = 3.2, 1.3 Hz, 2H), 7.55 (ddd, J = 8.7, 6.7, 1.3 Hz, 1H), 7.44 (td, J = 8.7, 1.4 Hz, 1H), 4.53 (s, 1H), 3.43 – 3.35 (m, 2H), 3.34 (dd, J = 5.1, 4.2 Hz, 2H).

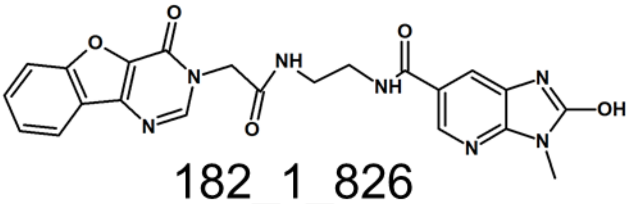

182_1_826

Synthesis of 182_1_826

4 mg of **826** was dissolved in 0.7 ml of DMF and was activated by HATU (1 eq, 9.8 mg in 100 µl DMF) and HOAt (1 eq, 3.5 mg in 100 µl) under argon protection for 20 min at RT. To the solution was added 1 eq of **182_1** (7.4 mg in 0.1 ml of DMF) dissolved with 5 eq of DIPEA (13.4 mg, 18 µl). The reaction was stirred for 3 h at RT. A small amount of reaction crude (2 µmol) was injected in UPLC-ESI-MS to evaluate the reaction yield. When the reaction product was detected, a two-fold volume of water was added to the reaction and the crude was freeze-

dried. The dried reaction crude was then dissolved in 0.2 ml DMSO and was purified by reverse-phase HPLC to obtain the reaction product. HPLC fractions were injected in UPLC-ESI-MS to identify the best fraction, and the desired fractions were freeze-dried yielding 4.3 mg (45%) of **182_1_826**. 1H NMR (600 MHz, DMSO-d6) δ 10.33 (s, 1H), 9.00 (s, 1H), 8.80 (d, J = 1.5 Hz, 1H), 8.54 (t, J = 5.3 Hz, 1H), 8.22 (d, J = 1.5 Hz, 1H), 8.10 (dd, J = 8.5, 1.3 Hz, 1H), 7.94 (t, J = 5.1 Hz, 1H), 7.64 (dd, J = 6.6, 1.3 Hz, 1H), 7.55 (ddd, J = 8.7, 6.7, 1.3 Hz, 1H), 7.44 (td, J = 8.7, 1.4 Hz, 1H), 4.53 (s, 1H), 3.71 (s, 2H), 3.43 – 3.37 (m, 2H), 3.34 (dd, J = 5.1, 4.2 Hz, 2H).

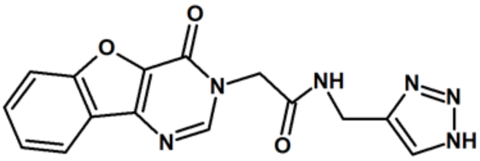

182_1_787

Synthesis of 182_1_787

5 mg of **182** was dissolved in 0.3 ml DMF and was activated by HATU (1 eq, 7.8 mg in 100 µl DMF) and HOAt (1 eq, 2.8 mg in 100 µl DMF) under argon protection for 20 min at RT. 1 eq of **787** (2 mg) was dissolved in 0.5 ml DMSO and pH was tuned to 8 by DIPEA, followed by addition of the activated **182**. The reaction was stirred for 3 h at RT. Small amount of reaction crude (2 µmol) was injected in UPLC-ESI-MS to evaluate the reaction yield. When the reaction product was detected, two-fold volume of water was added to the reaction and the crude was freeze-dried. The dried reaction crude was then dissolved in 0.2 ml DMSO and was purified by reverse-phase HPLC to obtain the reaction product. HPLC fractions were injected in UPLC-ESI-MS to identify the best fractions, and the desired fractions were freeze-dried yielding 4.98 mg (75%) of **182_1_787**. 1H NMR (600 MHz, DMSO-d6) δ 12.58 (d, J = 1.5 Hz, 1H), 9.00 (s, 1H), 8.55 (t, J = 6.1 Hz, 1H), 8.10 (dd, J = 8.6, 1.3 Hz, 1H), 8.07 (d, J = 1.2 Hz, 1H), 7.64 (dd, J = 6.6, 1.3 Hz, 1H), 7.55 (ddd, J = 8.7, 6.7, 1.3 Hz, 1H), 7.44 (td, J = 8.7, 1.4 Hz, 1H), 4.35 (d, J = 5.9 Hz, 2H).

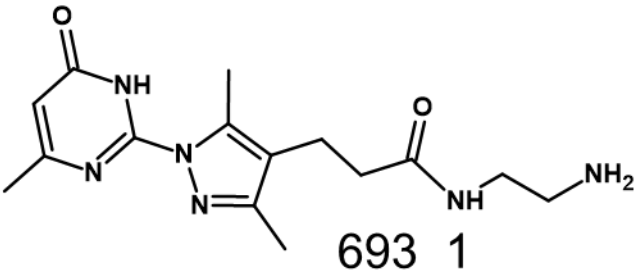

693_1

Synthesis of 693_1

100 mg of **693** was dissolved in 3 ml DMF and PyBOP (1 eq, 188 mg) in 1 ml DMF was added to activate for 20 min under argon atmosphere. Activated **693** was added drop by drop to ethylenediamine (5 eq, 108 mg, 120 µl) dissolved in 2 ml DMF while stirring. The reaction was stirred under argon protection for 3 h at RT. A Small amount of reaction crude (2 µmol) was injected in UPLC-ESI-MS to evaluate the reaction yield. When the reaction product was detected, a two-fold volume of water was added to the reaction, and the crude was freeze-dried. The dried reaction crude was then dissolved in 2 ml DMSO and was purified by reverse-phase HPLC to obtain the reaction product. HPLC fractions were injected in UPLC-ESI-MS to identify the best fraction, and the desired fractions were freeze-dried yielding 25 mg (22%) **693_1**.

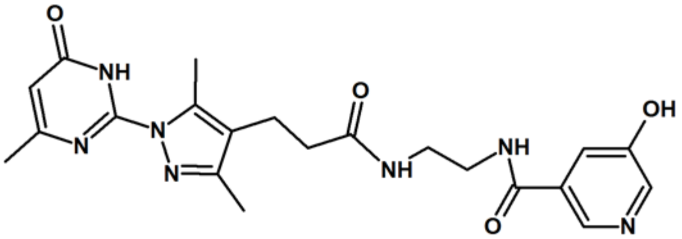

693_1_828

Synthesis of 693_1_828

5 mg of **828** was dissolved in 0.6 ml of DMF and was activated by HATU (1 eq, 13.7 mg in 100 µl DMF) and HOAt (1 eq, 4.9 mg in 100 µl) under argon protection for 20 min at RT. To the solution was added 1 eq of **693_1** (11.5 mg in 0.3 ml of DMF) dissolved with 5 eq of DIPEA (23.4 mg, 31.6 µl). The reaction was stirred for 3 h at RT. A small amount of reaction crude (2 µmol) was injected in UPLC-ESI-MS to evaluate the reaction yield. When the reaction product was detected, a two-fold volume of water was added to the reaction, and the crude was freeze-dried. The dried reaction crude was then dissolved in 0.2 ml DMSO and was purified by reverse-phase HPLC to obtain the reaction product. HPLC fractions were injected in UPLC-ESI-MS to identify the best fraction, and the desired fractions were freeze-dried yielding 5.8 mg (36%) of **693_1_828**. 1H NMR (600 MHz, DMSO-d6) δ 10.54 (s, 1H), 10.41 (s, 1H), 8.68 (t, J = 1.9 Hz, 1H), 8.52 (t, J = 5.2 Hz, 1H), 8.33 (t, J = 1.9 Hz, 1H), 7.66 – 7.61 (m, 2H), 5.82 (q, J = 1.2 Hz, 1H), 3.42 – 3.35 (m, 4H), 2.88 (dt, J = 17.7, 8.0 Hz, 1H), 2.80 – 2.69 (m, 1H), 2.51 – 2.45 (m, 5H), 2.44 (s, 2H), 2.17 (s, 2H).

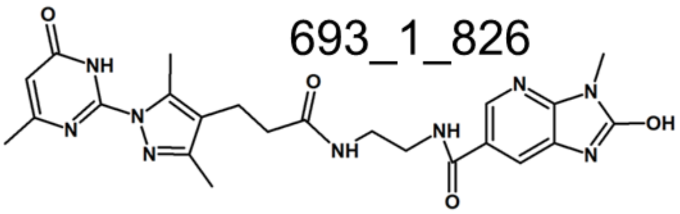

Synthesis of 693_1_826

4 mg of **826** was dissolved in 0.7 ml of DMF and was activated by HATU (1 eq, 9.8 mg in 100 µl DMF) and HOAt (1 eq, 3.5 mg in 100 µl) under argon protection for 20 min at RT. To the solution was added 1 eq of **693_1** (6.6 mg in 0.1 ml of DMF) dissolved with 5 eq of DIPEA (13.4 mg, 18 µl). The reaction was stirred for 3 h at RT. A small amount of reaction crude (2 µmol) was injected in UPLC-ESI-MS to evaluate the reaction yield. When the reaction product was detected, a two-fold volume of water was added to the reaction, and the crude was freeze-dried. The dried reaction crude was then dissolved in 0.2 ml DMSO and was purified by reverse-phase HPLC to obtain the reaction product. HPLC fractions were injected in UPLC-ESI-MS to identify the best fraction, and the desired fractions were freeze-dried yielding 7.3 mg (70%) of **693_1_826**. 1H NMR (600 MHz, DMSO-d6) δ 10.54 (s, 1H), 10.33 (s, 1H), 8.80 (d, J = 1.5 Hz, 1H), 8.54 (t, J = 5.1 Hz, 1H), 8.22 (d, J = 1.5 Hz, 1H), 7.64 (t, J = 4.9 Hz, 1H), 5.82 (q, J = 1.2 Hz, 1H), 3.73 (s, 2H), 3.42 – 3.35 (m, 4H), 2.87 (dt, J = 17.8, 8.0 Hz, 1H), 2.80 – 2.71 (m, 1H), 2.51 – 2.45 (m, 5H), 2.44 (s, 2H), 2.17 (s, 2H).

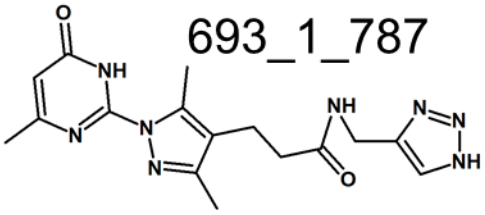

Synthesis of 693_1_787

5 mg of **693** was dissolved in 0.3 ml DMF and was activated by HATU (1 eq, 6.9 mg in 100 µl) and HOAt (1 eq, 2.5 mg in 100 µl) under argon protection for 20 min at RT. 1 eq of **787** (1.8 mg) was dissolved in 0.5 ml DMSO and pH was tuned to 8 by DIPEA, followed by addition to the activated **693**. The reaction was stirred for 3 h at RT. A small amount of reaction crude (2 µmol) was injected in UPLC-ESI-MS to evaluate the reaction yield. When the reaction product was detected, a two-fold volume of water was added to the reaction, and the crude was freeze-dried. The dried reaction crude was then dissolved in 0.2 ml DMSO and was purified by reverse-phase HPLC to obtain the reaction product. HPLC fractions were injected in UPLC-ESI-MS to identify the best fraction, and the desired fractions were freeze-dried yielding 1.8

mg (28%) of **693_1_787**. 1H NMR (600 MHz, DMSO-d6) δ 12.58 (d, J = 1.5 Hz, 1H), 10.54 (s, 1H), 8.41 (t, J = 6.1 Hz, 1H), 8.07 (d, J = 1.2 Hz, 1H), 5.82 (q, J = 1.2 Hz, 1H), 4.33 (d, J = 5.9 Hz, 2H), 2.88 (dt, J = 17.7, 8.0 Hz, 1H), 2.80 – 2.71 (m, 1H), 2.53 – 2.47 (m, 5H), 2.17 (s, 2H).

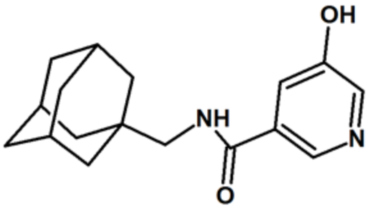

66_1_828

Synthesis of 66_1_828

5 mg of **828** was dissolved in 0.6 ml of DMF and was activated by HATU (1 eq, 13.7 mg in 100 µl DMF) and HOAt (1 eq, 4.9 mg in 100 µl) under argon protection for 20 min at RT. The solution was added 1 eq of 1-adamantane methyl amine (6 mg) dissolved in 0.1 ml of DMF dissolved with 5 eq of DIPEA (23.2 mg in 31.3 µl). The reaction was stirred for 3 h at RT. A small amount of reaction crude (2 µmol) was injected in UPLC-ESI-MS to evaluate the reaction yield. When the reaction product was detected, a two-fold volume of water was added to the reaction, and the crude was freeze-dried. The dried reaction crude was then dissolved in 0.2 ml DMSO and was purified by reverse-phase HPLC to obtain the reaction product. HPLC fractions were injected in UPLC-ESI-MS to identify the best fraction, and the desired fractions were freeze-dried yielding 6.4 mg (61%) of **66_1_828**. 1H NMR (600 MHz, DMSO-d6) δ 10.41 (s, 1H), 8.68 (t, J = 1.9 Hz, 1H), 8.33 (t, J = 1.9 Hz, 1H), 8.08 (t, J = 6.2 Hz, 1H), 7.63 (t, J = 2.0 Hz, 1H), 3.19 (dd, J = 13.7, 6.2 Hz, 1H), 3.12 (dd, J = 13.7, 6.2 Hz, 1H), 1.99 (dh, J = 10.9, 5.3 Hz, 3H), 1.65 (t, J = 5.8 Hz, 5H), 1.54 (s, 3H).

66_1_826

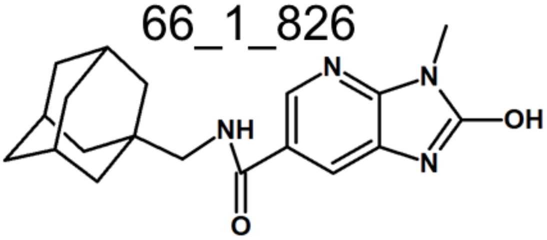

Synthesis of 66_1_826

5 mg of **826** was dissolved in 0.6 ml of DMF and was activated by HATU (1 eq, 9.8 mg in 100 µl DMF) and HOAt (1 eq, 3.5 mg in 100 µl DMF) under argon protection for 20 min at RT. To the solution was added 1 eq of 1-adamantane methyl amine (4.3 mg) dissolved in 0.1 ml of

DMF dissolved with 5 eq of DIPEA (16.7 mg, 22.6 µl). The reaction was stirred for 3 h at RT. A small amount of reaction crude (2 µmol) was injected in UPLC-ESI-MS to evaluate the reaction yield. When the reaction product was detected, a two-fold volume of water was added to the reaction, and the crude was freeze-dried. The dried reaction crude was then dissolved in 0.2 ml DMSO and was purified by reverse-phase HPLC to obtain the reaction product. HPLC fractions were injected in UPLC-ESI-MS to identify the best fraction, and the desired fractions were freeze-dried yielding 1.8 mg (21%) of **66_1_826**. 1H NMR (600 MHz, DMSO-d6) δ 10.33 (s, 1H), 8.80 (d, J = 1.5 Hz, 1H), 8.24 (d, J = 1.5 Hz, 1H), 8.10 (t, J = 6.1 Hz, 1H), 3.73 (s, 2H), 3.21 – 3.15 (m, 1H), 3.12 (dd, J = 13.9, 6.1 Hz, 1H), 1.99 (dh, J = 10.9, 5.3 Hz, 3H), 1.65 (t, J = 5.8 Hz, 5H), 1.55 (d, J = 5.0 Hz, 6H).

66_1_787

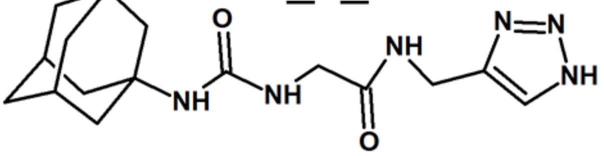

Synthesis of 66_1_787

5 mg of **66** was dissolved in 0.6 ml of DMF and was activated by HATU (1 eq, 7.5 mg in 100 µl) and HOAt (1 eq, 2.7 mg in 100 µl) under argon protection for 20 min at RT. 1 eq of **787** (1.94 mg) was dissolved in 0.5 ml DMSO and pH was tuned to 8 by DIPEA, followed by addition to the activated **66**. The reaction was stirred for 3 h at RT. A small amount of reaction crude (2 µmol) was injected in UPLC-ESI-MS to evaluate the reaction yield. When the reaction product was detected, a two-fold volume of water was added to the reaction and the crude was freeze-dried. The dried reaction crude was then dissolved in 0.2 ml DMSO and was purified by reverse-phase HPLC to obtain the reaction product. HPLC fractions were injected in UPLC-ESI-MS to identify the best fraction, and the desired fractions were freeze-dried yielding 3.7 mg (56%) of **66_1_787**. 1H NMR (600 MHz, DMSO-d6) δ 12.58 (d, J = 1.5 Hz, 1H), 8.41 (d, J = 11.5 Hz, 1H), 8.41 (s, 1H), 8.07 (d, J = 1.2 Hz, 1H), 6.11 (t, J = 5.3 Hz, 1H), 5.46 (s, 1H), 4.35 (d, J = 5.6 Hz, 2H), 3.75 (d, J = 5.3 Hz, 2H), 2.05 (dq, J = 10.6, 5.3 Hz, 3H), 1.95 (d, J = 5.2 Hz, 6H), 1.65 (t, J = 5.4 Hz, 6H).

Synthesis of fragment pairs linked by linker 4.

182_4

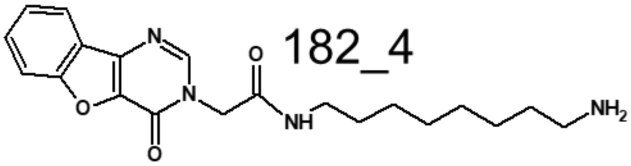

Synthesis of 182_4

50 mg of **182** was dissolved in 2ml DMF and PyBOP (1 eq, 105 mg) in 1 ml DMF was added to activate for 20 min under argon atmosphere. Activated **182** was added drop by drop to octane-1,8 diamine (5 eq, 150 mg) dissolved in 2 ml DMF while stirring. The reaction was stirred under argon protection for 3 h at RT. A small amount of reaction crude (2 µmol) was injected in UPLC-ESI-MS to evaluate the reaction yield. When the reaction product was detected, a two-fold volume of water was added to the reaction and the crude was freeze-dried. The dried reaction crude was then dissolved in 2 ml DMSO and was purified by reverse-phase HPLC to obtain the reaction product. HPLC fractions were injected in UPLC-ESI-MS to identify the best fraction, and the desired fractions were freeze-dried yielding 35 mg (46%) **182_4**.

182_4_828

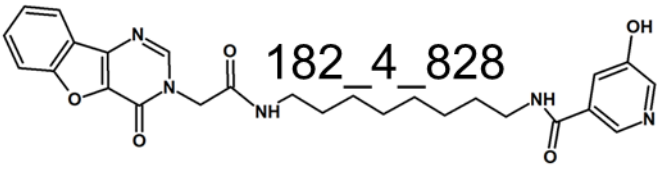

Synthesis of 182_4_828

5 mg of **828** was dissolved in 0.6 ml of DMF and was activated by HATU (1 eq, 13.7 mg in 100 µl DMF) and HOAt (1 eq, 4.9 mg in 100 µl) under argon protection for 20 min at RT. To the solution was added 1 eq of **182_4** (7.6 mg in 0.1 ml of DMF) dissolved with 5 eq of DIPEA (13 mg, 17.8 µl). The reaction was stirred for 3 h at RT. A small amount of reaction crude (2 µmol) was injected in UPLC-ESI-MS to evaluate the reaction yield. When the reaction product was detected, a two-fold volume of water was added to the reaction and the crude was freeze-dried. The dried reaction crude was then dissolved in 0.2 ml DMSO and was purified by reverse-phase HPLC to obtain the reaction product. HPLC fractions were injected in UPLC-ESI-MS to identify the best fraction, and the desired fractions were freeze-dried yielding 6.6 mg (36%) of **182_4_828**. 1H NMR (600 MHz, DMSO-d6) δ 10.41 (s, 1H), 9.00 (s, 1H), 8.66 (t, J = 1.7 Hz, 1H), 8.37 (t, J = 5.1 Hz, 1H), 8.33 (t, J = 1.9 Hz, 1H), 8.10 (dd, J = 8.6, 1.4 Hz, 1H), 7.75 (t, J = 5.0 Hz, 1H), 7.67 – 7.61 (m, 2H), 7.58 – 7.52 (m, 1H), 7.44 (td, J = 8.7, 1.4 Hz, 1H), 3.24 (q, J = 5.3 Hz, 2H), 3.14 (q, J = 5.3 Hz, 2H), 1.57 (tt, J = 7.5, 5.6 Hz, 2H), 1.50 (tt, J = 7.7, 5.5 Hz, 2H), 1.37 – 1.30 (m, 2H), 1.32 – 1.25 (m, 6H).

182_4_826

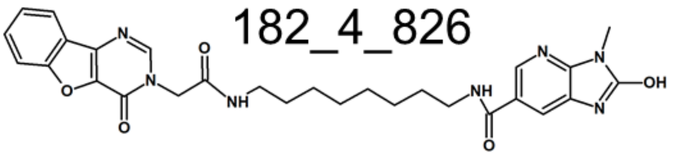

Synthesis of 182_4_826

4 mg of **826** was dissolved in 0.7 ml of DMF and was activated by HATU (1 eq, 9.8 mg in 100 µl DMF) and HOAt (1 eq, 3.5 mg in 100 µl) under argon protection for 20 min at RT. To the solution, 1 eq of **182_4** (7.7 mg in 0.1 ml of DMF) dissolved with 5 eq of DIPEA (13.4 mg in 18 µl) was added. The reaction was stirred for 3 h at RT. A small amount of reaction crude (2 µmol) was injected in UPLC-ESI-MS to evaluate the reaction yield. When the reaction product was detected, a two-fold volume of water was added to the reaction, and the crude was freeze-dried. The dried reaction crude was then dissolved in 0.2 ml DMSO and was purified by reverse-phase HPLC to obtain the reaction product. HPLC fractions were injected in UPLC-ESI-MS to identify the best fraction, and the desired fractions were freeze-dried yielding 2.5 mg (22%) of **182_4_826**. 1H NMR (600 MHz, DMSO-d6) δ 10.33 (s, 1H), 9.00 (s, 1H), 8.80 (d, J = 1.5 Hz, 1H), 8.39 (t, J = 5.0 Hz, 1H), 8.24 (d, J = 1.5 Hz, 1H), 8.10 (dd, J = 8.6, 1.2 Hz, 1H), 7.75 (t, J = 5.0 Hz, 1H), 7.64 (dd, J = 6.6, 1.3 Hz, 1H), 7.55 (ddd, J = 8.7, 6.7, 1.3 Hz, 1H), 7.44 (td, J = 8.7, 1.4 Hz, 1H), 4.53 (s, 1H), 3.23 (q, J = 5.3 Hz, 2H), 3.17 – 3.12 (m, 2H), 1.57 (tt, J = 7.6, 5.6 Hz, 2H), 1.50 (tt, J = 7.7, 5.6 Hz, 2H), 1.37 – 1.31 (m, 2H), 1.33 – 1.25 (m, 6H).

182_4_787

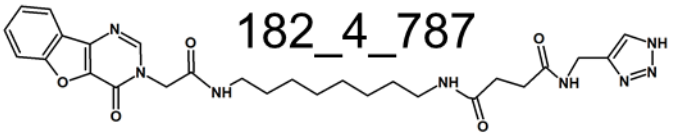

Synthesis of 182_4_787

8 mg (1 eq) of **182_4** was dissolved in 0.3 ml of DMSO. 1.1 eq of succinic anhydride (2.4 mg in 0.5 ml DMSO) was added to **182_4** to convert terminal amino group the carboxylic group. After 30 min stirring, the resulting product was confirmed by UPLC-ESI-MS. Further, 1.3 eq of HATU (11.9 mg in 100 µl) and 1.3 eq of HOAt (4.2 mg in 100 µl) was added to activate the carboxylic group. After 20 min activation under argon protection, 2 eq of **787** (4.7 mg in 0.5 ml DMSO) was added to the crude. The reaction was stirred for 3h at RT. A small amount of reaction crude (2 µmol) was injected in UPLC-ESI-MS to evaluate the reaction yield. When the reaction product was detected, a two-fold volume of water was added to the reaction and the crude was freeze-dried. The dried reaction crude was then dissolved in 0.2 ml DMSO and was purified by reverse-phase HPLC to obtain the reaction product. HPLC fractions were

injected in UPLC-ESI-MS to identify the best fraction, and the desired fractions were freeze-dried yielding 8.2 mg (62%) of **182_4_787**. 1H NMR (600 MHz, DMSO-d6) δ 12.58 (d, J = 1.5 Hz, 1H), 9.00 (s, 1H), 8.38 (t, J = 6.1 Hz, 1H), 8.12 – 8.06 (m, 2H), 7.75 (t, J = 5.0 Hz, 1H), 7.67 – 7.59 (m, 2H), 7.58 – 7.52 (m, 1H), 7.44 (td, J = 8.7, 1.4 Hz, 1H), 4.53 (s, 1H), 4.33 (d, J = 5.9 Hz, 2H), 3.13 (dt, J = 10.6, 5.3 Hz, 4H), 2.43 – 2.33 (m, 4H), 1.54 – 1.41 (m, 4H), 1.34 – 1.26 (m, 7H).

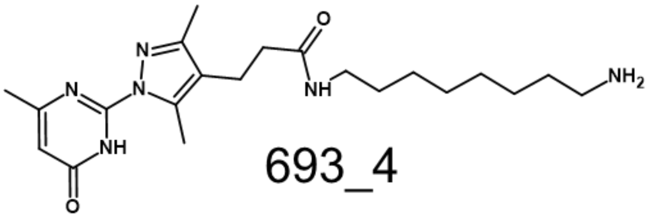

Synthesis of 693_4

100 mg of **693** was dissolved in 3 ml DMF and PyBOP (1 eq, 188 mg) in 1 ml DMF was added to activate for 20 min under argon atmosphere. Activated **693** was added drop by drop to octane-1,8 diamine (5 eq, 261 mg) dissolved in 2 ml DMF while stirring. The reaction was stirred under argon protection for 3 h at RT. A small amount of reaction crude (2 µmol) was injected in UPLC-ESI-MS to evaluate the reaction yield. When the reaction product was detected, a two-fold volume of water was added to the reaction and the crude was freeze-dried. The dried reaction crude was then dissolved in 2 ml DMSO and was purified by reverse-phase HPLC to obtain the reaction product. HPLC fractions were injected in UPLC-ESI-MS to identify the best fraction, and the desired fractions were freeze-dried leading to 47 mg (32%) of **693_4**.

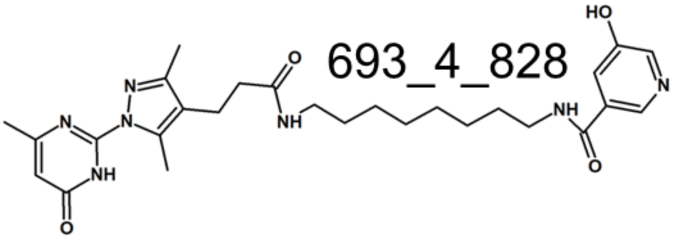

Synthesis of 693_4_828

5 mg of **828** was dissolved in 0.6 ml of DMF and was activated by HATU (1 eq, 13.7 mg in 100 µl DMF) and HOAt (1 eq, 4.9 mg in 100 µl) under argon protection for 20 min at RT. To the solution, 1 eq (14 mg) of **693_4** dissolved in 0.1 ml of DMF dissolved with 5 eq of DIPEA (13 mg, 17.8 µl) was added. The reaction was stirred for 3 h at RT. A small amount of reaction crude (2 µmol) was injected in UPLC-ESI-MS to evaluate the reaction yield. When the reaction

product was detected, a two-fold volume of water was added to the reaction, and the crude was freeze-dried. The dried reaction crude was then dissolved in 0.2 ml DMSO and was purified by reverse-phase HPLC to obtain the reaction product. HPLC fractions were injected in UPLC-ESI-MS to identify the best fraction, and the desired fractions were freeze-dried yielding 10.5 mg (55.6%) of **693_4_828**. 1H NMR (600 MHz, DMSO-d6) δ 10.54 (s, 1H), 10.41 (s, 1H), 8.66 (t, J = 1.7 Hz, 1H), 8.37 (t, J = 5.1 Hz, 1H), 8.33 (t, J = 1.9 Hz, 1H), 7.63 (t, J = 2.0 Hz, 1H), 7.53 (s, 1H), 7.53 (d, J = 9.6 Hz, 0H), 5.82 (q, J = 1.2 Hz, 1H), 3.24 (q, J = 5.5 Hz, 2H), 3.07 (q, J = 5.5 Hz, 2H), 2.88 (dt, J = 17.7, 8.0 Hz, 1H), 2.80 – 2.71 (m, 1H), 2.49 (d, J = 1.2 Hz, 3H), 2.47 (t, J = 8.1 Hz, 2H), 2.44 (s, 2H), 1.57 (tt, J = 7.6, 5.4 Hz, 2H), 1.45 (ddt, J = 8.0, 7.2, 5.3 Hz, 2H), 1.37 – 1.25 (m, 8H).

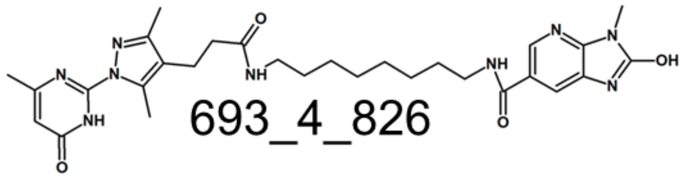

Synthesis of 693_4_826

4 mg of **826** was dissolved in 0.7 ml of DMF and was activated by HATU (1 eq, 9.8 mg in 100 µl DMF) and HOAt (1 eq, 3.5 mg in 100 µl) under argon protection for 20 min at RT. To the solution was added 1 eq (8.4 mg) of **693_4** dissolved in 0.1 ml of DMF dissolved with 5 eq of DIPEA. The reaction was stirred for 3 h at RT. A small amount of reaction crude (2 µmol) was injected in UPLC-ESI-MS to evaluate the reaction yield. When the reaction product was detected, a two-fold volume of water was added to the reaction and the crude was freeze-dried. The dried reaction crude was then dissolved in 0.2 ml DMSO and was purified by reverse-phase HPLC to obtain the reaction product. HPLC fractions were injected in UPLC-ESI-MS to identify the best fraction, and the desired fractions were freeze-dried yielding 7.8 mg (64.9%) of **693_4_826**. 1H NMR (600 MHz, DMSO-d6) δ 10.54 (s, 1H), 10.33 (s, 1H), 8.80 (d, J = 1.5 Hz, 1H), 8.39 (t, J = 5.0 Hz, 1H), 8.24 (d, J = 1.5 Hz, 1H), 7.53 (s, 1H), 7.53 (d, J = 9.6 Hz, 1H), 5.82 (q, J = 1.2 Hz, 1H), 3.26 – 3.21 (m, 2H), 3.07 (q, J = 5.5 Hz, 2H), 2.88 (dt, J = 17.7, 8.0 Hz, 1H), 2.80 – 2.71 (m, 1H), 2.49 (d, J = 1.2 Hz, 3H), 2.47 (t, J = 8.1 Hz, 2H), 2.44 (s, 2H), 2.17 (s, 2H), 1.57 (tt, J = 7.5, 5.3 Hz, 2H), 1.45 (ddt, J = 8.0, 7.2, 5.3 Hz, 2H), 1.37 – 1.25 (m, 9H).

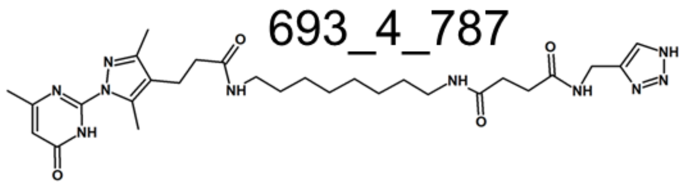

Synthesis of 693_4_787

8 mg of **693_4** was dissolved in 0.2 ml DMSO. 26 µl of DIPEA was added to tune the pH to 8, and 3 mg of succinic anhydride (1.5 eq) was added to the mixture. The reaction was stirred at RT for 30 min, and reaction product was validated by UPLC-ESI-MS. The product was activated by 11.4 mg of HATU in 100 µl of DMSO and 2.53 mg of HOAt in 100 µl of DMSO under argon protection for 20 min at RT. To the activated carboxylic acid was added 5.2 mg of **787** dissolved in 200 µl of DMSO. When the reaction product was detected, a two-fold volume of water was added to the reaction and the crude was freeze-dried. The dried reaction crude was then dissolved in 0.2 ml DMSO and was purified by reverse-phase HPLC to obtain the reaction product. HPLC fractions were injected in UPLC-ESI-MS to identify the best fraction, and the desired fractions were freeze-dried yielding 8.7 mg (78.9%) of **693_4_787**. 1H NMR (600 MHz, DMSO-d6) δ 12.58 (d, J = 1.5 Hz, 1H), 10.54 (s, 0H), 8.38 (t, J = 6.1 Hz, 1H), 8.07 (d, J = 1.2 Hz, 1H), 5.82 (q, J = 1.4 Hz, 1H), 4.33 (d, J = 5.9 Hz, 2H), 3.10 (dq, J = 33.4, 5.2 Hz, 3H), 2.88 (dt, J = 17.7, 8.0 Hz, 1H), 2.76 (dt, J = 17.4, 8.0 Hz, 1H), 2.49 (s, 1H), 2.51 – 2.43 (m, 4H), 2.43 – 2.33 (m, 3H), 1.45 (tt, J = 7.6, 5.5 Hz, 3H), 1.34 – 1.26 (m, 6H).

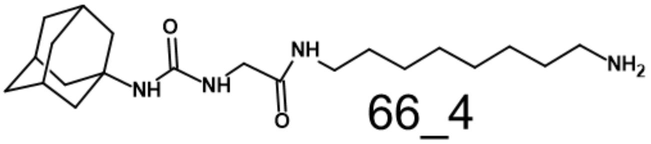

66_4

Synthesis of 66_4

100 mg of **66** was dissolved in 5 ml DMF and PyBOP (1 eq, 206 mg) in 1 ml DMF was added to activate for 20 min under argon atmosphere. Activated **66** was added drop by drop to octane-1,8-diamine (5 eq, 286 mg) dissolved in 2 ml DMF while stirring. The reaction was stirred under argon protection for 3 h at RT. A small amount of reaction crude (2 µmol) was injected in UPLC-ESI-MS to evaluate the reaction yield. When the reaction product was detected, a two-fold volume of water was added to the reaction, and the crude was freeze-dried. The dried reaction crude was then dissolved in 2 ml DMSO and was purified by reverse-phase HPLC to obtain the reaction product. HPLC fractions were injected in UPLC-ESI-MS to identify the best fraction, and the desired fractions were freeze-dried yielding 78 mg (52%) of **66_4**.

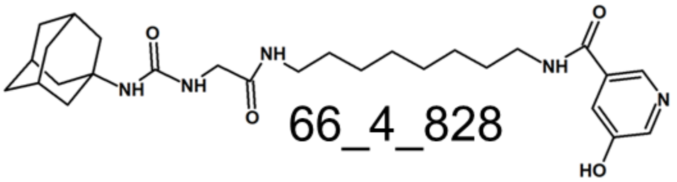

66_4_828

Synthesis of 66_4_828

5 mg of **828** was dissolved in 0.6 ml of DMF and was activated by HATU (1 eq, 13.7 mg in 100 µl DMF) and HOAt (1 eq, 4.9 mg in 100 µl) under argon protection for 20 min at RT. To the solution was added 1 eq (13.6 mg) of **66_4** dissolved in 0.1 ml of DMF dissolved with 5 eq

of DIPEA (13 mg, 17.8 µl). The reaction was stirred for 3 h at RT. A small amount of reaction crude (2 µmol) was injected in UPLC-ESI-MS to evaluate the reaction yield. When the reaction product was detected, a two-fold volume of water was added to the reaction and the crude was freeze-dried. The dried reaction crude was then dissolved in 0.2 ml DMSO and was purified by reverse-phase HPLC to obtain the reaction product. HPLC fractions were injected in UPLC-ESI-MS to identify the best fraction, and the desired fractions were freeze-dried yielding 4.9 mg (27%) of **66_4_828**. 1H NMR (600 MHz, DMSO-d6) δ 10.41 (s, 1H), 8.66 (t, J = 1.7 Hz, 1H), 8.37 (t, J = 5.1 Hz, 1H), 8.33 (t, J = 1.9 Hz, 1H), 7.63 (t, J = 2.0 Hz, 1H), 7.60 (t, J = 4.7 Hz, 1H), 6.11 (t, J = 5.3 Hz, 1H), 5.46 (s, 1H), 3.73 (d, J = 5.3 Hz, 2H), 3.24 (q, J = 5.3 Hz, 2H), 3.13 (td, J = 5.6, 4.6 Hz, 2H), 2.05 (hept, J = 5.3 Hz, 3H), 1.94 (s, 2H), 1.65 (t, J = 5.4 Hz, 5H), 1.57 (tt, J = 7.6, 5.6 Hz, 2H), 1.50 (tt, J = 7.7, 5.6 Hz, 2H), 1.37 – 1.30 (m, 2H), 1.32 – 1.25 (m, 6H).

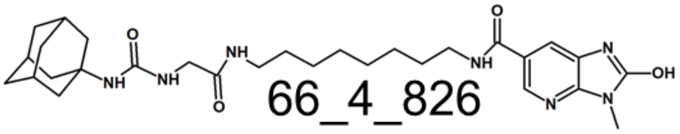

Synthesis of 66_4_826

4 mg of **826** was dissolved in 0.7 ml of DMF and was activated by HATU (1 eq, 9.8 mg in 100 µl DMF) and HOAt (1 eq, 3.5 mg in 100 µl) under argon protection for 20 min at RT. To the solution was added 1 eq (7.8 mg) **4** dissolved in 0.1 ml of DMF dissolved with 5 eq of DIPEA (13.3 mg, 18 µl). The reaction was stirred for 3 h at RT. A small amount of reaction crude (2 µmol) was injected in UPLC-ESI-MS to evaluate the reaction yield. When the reaction product was detected, a two-fold volume of water was added to the reaction and the crude was freeze-dried. The dried reaction crude was then dissolved in 0.2 ml DMSO and was purified by reverse-phase HPLC to obtain the reaction product. HPLC fractions were injected in UPLC-ESI-MS to identify the best fraction, and the desired fractions were freeze-dried yielding 3.6 mg (32%) of **66_4_826**. 1H NMR (600 MHz, DMSO-d6) δ 10.33 (s, 1H), 8.80 (d, J = 1.5 Hz, 1H), 8.39 (t, J = 5.0 Hz, 1H), 8.24 (d, J = 1.5 Hz, 1H), 7.60 (t, J = 4.7 Hz, 1H), 6.11 (t, J = 5.3 Hz, 1H), 5.46 (s, 1H), 3.73 (d, J = 5.3 Hz, 2H), 3.23 (q, J = 5.3 Hz, 2H), 3.13 (td, J = 5.5, 4.6 Hz, 2H), 2.05 (p, J = 5.3 Hz, 3H), 1.95 (d, J = 5.2 Hz, 6H), 1.65 (t, J = 5.4 Hz, 5H), 1.57 (tt, J = 7.6, 5.6 Hz, 2H), 1.50 (tt, J = 7.7, 5.6 Hz, 2H), 1.37 – 1.30 (m, 2H), 1.30 (tdd, J = 5.0, 3.9, 2.3 Hz, 6H).

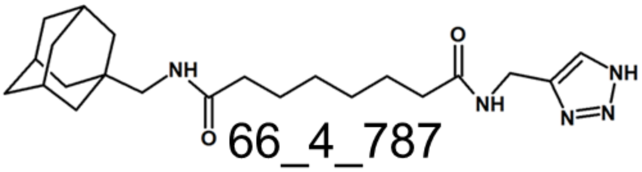

66_4_787

Synthesis of 66_4_787

100 mg of Suberic acid was dissolved in 1 ml DMF. To the solution, was added 230 mg of HATU (230 mg in 1 ml DMF) and HOAt (80 mg in 0.3 ml DMF) drop by drop to activate the carboxylic acid. To the activated suberic acid, 20 mg of 1-adamantane methylamine in 0.2 ml of DMF was added drop by drop. The reaction was stirred for 3 h at RT. A small amount of reaction crude (2 µmol) was injected in UPLC-ESI-MS to evaluate the reaction yield. When the reaction product was detected, a two-fold volume of water was added to the reaction and the crude was freeze-dried. 20 mg of the obtained reaction product was activated by 1 eq (23.6 mg) HATU and 1 eq (8.5 mg) HOAt under argon protection for 20 min at RT. 11 mg of **787** in 0.2 ml of DMF was added to the crude. Reaction was stirred for 3 h at RT. A small amount of reaction crude (2 µmol) was injected in UPLC-ESI-MS to evaluate the reaction yield. When the reaction product was detected, a two-fold volume of water was added to the reaction, and the crude was freeze-dried yielding 15.3 mg (34%) of **66_4_787**. 1H NMR (600 MHz, DMSO-d6) δ 12.58 (d, J = 1.5 Hz, 1H), 8.27 (t, J = 6.1 Hz, 1H), 8.07 (d, J = 1.2 Hz, 1H), 7.12 (t, J = 6.1 Hz, 1H), 4.33 (d, J = 5.9 Hz, 2H), 2.99 (d, J = 6.2 Hz, 2H), 2.20 (dt, J = 16.2, 8.6 Hz, 4H), 2.03 – 1.95 (m, 3H), 1.64 (t, J = 5.8 Hz, 5H), 1.57 (qd, J = 8.3, 7.3 Hz, 4H), 1.48 (s, 3H), 1.38 – 1.30 (m, 4H).

Synthesis of fragment pairs linked by linker 10.

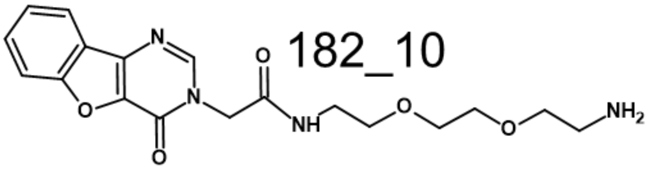

182_10

Synthesis of 182_10

100 mg 182 in 1 ml DMF was activated by 1 eq (155 mg) HATU and 1 eq (55.8 mg) HOAt at RT for 20 min under argon atmosphere. Activated 182 was added to 2,2′-(ethylenedioxy) bis(ethylamine) (5 eq, 303 mg) dissolved in 0.5 ml DMF while stirring. The reaction was stirred under argon protection for 3 h at RT. A small amount of reaction crude (2 µmol) was injected in UPLC-ESI-MS to evaluate the reaction yield. When the reaction product was detected, a two-fold volume of water was added to the reaction and the crude was freeze-dried. The dried

reaction crude was then dissolved in 2 ml DMSO and was purified by reverse-phase HPLC to obtain the reaction product. HPLC fractions were injected in UPLC-ESI-MS to identify the best fraction, and the desired fractions were freeze-dried yielding 105 mg (69%) of **182_10**.

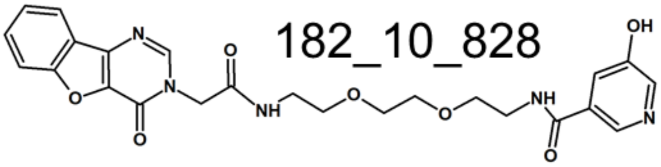

Synthesis of 182_10_828

5 mg of **828** was dissolved in 0.6 ml of DMF and was activated by HATU (1 eq, 13.7 mg in 100 µl DMF) and HOAt (1 eq, 4.9 mg in 100 µl) under argon protection for 20 min at RT. To the activated **828,** 13.4 mg of **182_10** in 0.5 ml DMF was added. The reaction was stirred under argon protection for 3 h at RT. A small amount of reaction crude (2 µmol) was injected in UPLC-ESI-MS to evaluate the reaction yield. When the reaction product was detected, a two-fold volume of water was added to the reaction and the crude was freeze-dried yielding 13.2 mg (74%) of **182_10_828**. 1H NMR (600 MHz, DMSO-d6) δ 10.41 (s, 1H), 9.00 (s, 1H), 8.68 (t, J = 1.9 Hz, 2H), 8.45 (d, J = 10.3 Hz, 1H), 8.45 (s, 1H), 8.33 (t, J = 1.9 Hz, 2H), 8.10 (dd, J = 8.6, 1.3 Hz, 2H), 7.96 (d, J = 10.3 Hz, 1H), 7.96 (s, 1H), 7.64 (td, J = 3.2, 1.3 Hz, 4H), 7.55 (ddd, J = 8.7, 6.7, 1.3 Hz, 2H), 7.44 (td, J = 8.7, 1.4 Hz, 2H), 4.53 (s, 3H), 3.57 (dt, J = 22.6, 4.2 Hz, 8H), 3.46 (dt, J = 5.1, 4.1 Hz, 4H), 3.33 (dt, J = 5.1, 4.2 Hz, 4H).

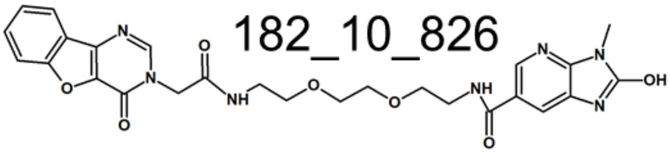

Synthesis of 182_10_826

4 mg of **826** was dissolved in 0.7 ml of DMF and was activated by HATU (1 eq, 9.8 mg in 100 µl DMF) and HOAt (1 eq, 3.5 mg in 100 µl) under argon protection for 20 min at RT. To the activated **826** was added 7.8 mg of **182_10** in 0.5 ml DMF. The reaction was stirred under argon protection for 3 h at RT. A small amount of reaction crude (2 µmol) was injected in UPLC-ESI-MS to evaluate the reaction yield. When the reaction product was detected, a two-fold volume of water was added to the reaction and the crude was freeze-dried yielding 5.4 mg (48%) of **182_10_826**. 1H NMR (600 MHz, DMSO-d6) δ 10.33 (s, 1H), 9.00 (s, 1H), 8.80 (d, J = 1.5 Hz, 1H), 8.44 (t, J = 5.3 Hz, 1H), 8.24 (d, J = 1.5 Hz, 1H), 8.10 (dd, J = 8.6, 1.3 Hz, 1H), 7.96 (d, J = 10.3 Hz, 1H), 7.96 (s, 1H), 7.66 – 7.62 (m, 1H), 7.55 (ddd, J = 8.7, 6.7, 1.2 Hz, 1H), 7.44 (td, J = 8.7, 1.4 Hz, 1H), 4.53 (s, 1H), 3.73 (s, 2H), 3.57 (dt, J = 22.7, 4.2 Hz, 4H), 3.46 (dt, J = 5.1, 4.1 Hz, 2H), 3.33 (dt, J = 5.1, 4.2 Hz, 2H).

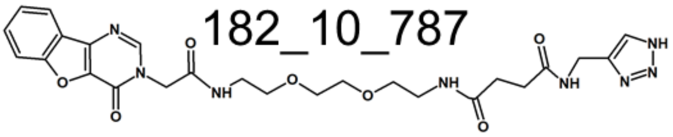

182_10_787

Synthesis of 182_10_787

10 mg of **182_10** was dissolved in 0.3 ml DMSO. To the solution was added 4 mg of succinic anhydride in 0.3 ml DMSO. The reaction was stirred for 30 min at RT and the product was validated by UPLC-ESI-MS. The product was activated by 1 eq HATU and 1 eq HOAt RT for 20 min under argon atmosphere. 4 mg (1.5 eq) of **787** was dissolved 0.3 ml DMSO and added to the crude. When the reaction product was detected, a two-fold volume of water was added to the reaction, and the crude was freeze-dried. The dried reaction crude was then dissolved in 0.2 ml DMSO and was purified by reverse-phase HPLC to obtain the reaction product. HPLC fractions were injected in UPLC-ESI-MS to identify the best fraction, and the desired fractions were freeze-dried yielding 6.5 mg (25%) of **182_110_787**. 1H NMR (600 MHz, DMSO-d6) δ 12.58 (d, J = 1.5 Hz, 1H), 9.00 (s, 1H), 8.38 (t, J = 6.1 Hz, 1H), 8.10 (dd, J = 8.6, 1.3 Hz, 1H), 8.07 (d, J = 1.2 Hz, 1H), 7.96 (d, J = 10.3 Hz, 0H), 7.96 (s, 1H), 7.64 (dd, J = 6.6, 1.3 Hz, 1H), 7.55 (ddd, J = 8.7, 6.7, 1.2 Hz, 1H), 7.47 – 7.41 (m, 2H), 4.53 (s, 1H), 4.33 (d, J = 5.9 Hz, 2H), 3.55 (td, J = 4.2, 2.1 Hz, 4H), 3.33 (dq, J = 5.3, 4.3 Hz, 4H), 2.42 – 2.33 (m, 4H).

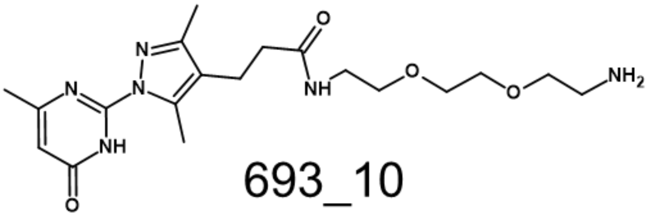

693_10

Synthesis of 693_10

100 mg of **693** was dissolved in 3 ml DMF and PyBOP (1 eq, 188 mg) in 1 ml DMF was added to activate for 20 min under argon atmosphere. Activated **693** was added to 2,2′-(ethylenedioxy) bis(ethylamine) (5 eq, 53.6 mg) dissolved in 0.5 ml DMF while stirring. The reaction was stirred under argon protection for 3 h at RT. A small amount of reaction crude (2 μmol) was injected in UPLC-ESI-MS to evaluate the reaction yield. When the reaction product was detected, a two-fold volume of water was added to the reaction and the crude was freeze-dried. The dried reaction crude was then dissolved in 2 ml DMSO and was purified by reverse-phase HPLC to obtain the reaction product. HPLC fractions were injected in UPLC-ESI-MS to identify the best fraction, and the desired fractions were freeze-dried yielding 88 mg (61%) of **693_10**.

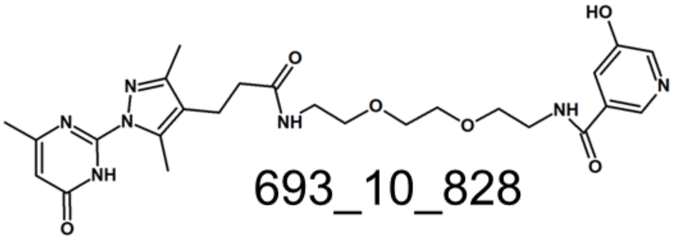

Synthesis of 693_10_828

5 mg of **828** was dissolved in 0.6 ml of DMF and was activated by HATU (1 eq, 13.7 mg in 100 µl DMF) and HOAt (1 eq, 4.9 mg in 100 µl) under argon protection for 20 min at RT. To the activated **828** was added 14.7 mg of **693_10** in 0.5 ml DMF. The reaction was stirred under argon protection for 3 h at RT. A small amount of reaction crude (2 µmol) was injected in UPLC-ESI-MS to evaluate the reaction yield. When the reaction product was detected, a two-fold volume of water was added to the reaction, and the crude was freeze-dried yielding 8.2 mg (43%) of **693_10_828**. 1H NMR (600 MHz, DMSO-d6) δ 10.54 (s, 1H), 10.41 (s, 1H), 8.68 (t, J = 1.9 Hz, 2H), 8.45 (d, J = 10.3 Hz, 1H), 8.45 (s, 1H), 8.33 (t, J = 1.9 Hz, 2H), 7.64 (t, J = 2.0 Hz, 2H), 7.53 (s, 1H), 7.53 (d, J = 10.3 Hz, 1H), 5.82 (q, J = 1.2 Hz, 2H), 3.57 (dt, J = 20.7, 4.2 Hz, 8H), 3.46 (dt, J = 5.2, 4.1 Hz, 4H), 3.32 (dt, J = 5.1, 4.3 Hz, 4H), 2.93 – 2.87 (m, 1H), 2.86 (d, J = 8.1 Hz, 1H), 2.80 – 2.71 (m, 2H), 2.51 – 2.43 (m, 14H), 2.17 (s, 4H).

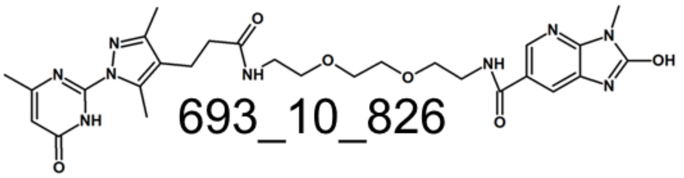

Synthesis of 693_10_826

4 mg of **826** was dissolved in 0.7 ml of DMF and was activated by HATU (1 eq, 9.8 mg in 100 µl DMF) and HOAt (1 eq, 3.5 mg in 100 µl) under argon protection for 20 min at RT. To the activated **826** was added 8.4 mg of **693_10** in 0.5 ml DMF. The reaction was stirred under argon protection for 3 h at RT. Small amount of reaction crude (2 µmol) was injected in UPLC-ESI-MS to evaluate the reaction yield. When the reaction product was detected, two-fold volume of water was added to the reaction and the crude was freeze-dried yielding 6.9 mg (58%) of **693_10_826**. 1H NMR (600 MHz, DMSO-d6) δ 10.54 (s, 1H), 10.33 (s, 1H), 8.80 (d, J = 1.5 Hz, 1H), 8.44 (t, J = 5.3 Hz, 1H), 8.24 (d, J = 1.5 Hz, 1H), 7.53 (s, 1H), 7.53 (d, J = 10.3 Hz, 1H), 5.82 (q, J = 1.3 Hz, 1H), 3.73 (s, 2H), 3.57 (dt, J = 20.7, 4.2 Hz, 4H), 3.46 (dt, J = 5.1, 4.1 Hz, 2H), 3.32 (dt, J = 5.1, 4.2 Hz, 2H), 2.88 (dt, J = 17.7, 8.0 Hz, 1H), 2.80 – 2.71 (m, 1H), 2.51 – 2.45 (m, 5H), 2.44 (s, 2H), 2.17 (s, 2H).

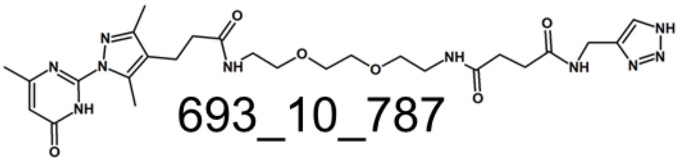

693_10_787

Synthesis of 693_10_787

10 mg of **693_10** was dissolved in 0.3 ml DMSO. To the solution was added 4 mg of succinic anhydride in 0.3 ml DMSO. The reaction was stirred for 30 min at RT and the product was validated by UPLC-ESI-MS. The product was activated by 1 eq HATU and 1 eq HOAt RT for 20 min under argon atmosphere.3.6 mg (1.5 eq) of **787** was dissolved 0.3 ml DMSO and added to the crude. When the reaction product was detected, a two-fold volume of water was added to the reaction, and the crude was freeze-dried. The dried reaction crude was then dissolved in 0.2 ml DMSO and was purified by reverse-phase HPLC to obtain the reaction product. HPLC fractions were injected in UPLC-ESI-MS to identify the best fraction, and the desired fractions were freeze-dried yielding 7.4 mg (54%) of **693_10_787**. 1H NMR (600 MHz, DMSO-d6) δ 12.58 (d, J = 1.5 Hz, 1H), 10.54 (s, 1H), 8.38 (t, J = 6.1 Hz, 1H), 8.07 (d, J = 1.2 Hz, 1H), 7.53 (s, 1H), 7.53 (d, J = 10.3 Hz, 0H), 7.44 (t, J = 5.0 Hz, 1H), 5.82 (q, J = 1.3 Hz, 1H), 4.33 (d, J = 5.9 Hz, 2H), 3.55 (t, J = 4.3 Hz, 4H), 3.35 – 3.29 (m, 4H), 2.88 (dt, J = 17.7, 8.0 Hz, 1H), 2.76 (dt, J = 17.4, 8.0 Hz, 1H), 2.49 – 2.44 (m, 3H), 2.42 – 2.33 (m, 4H), 2.17 (s, 2H).

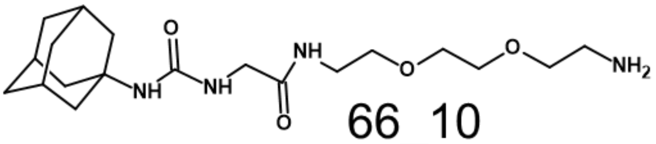

66_10

Synthesis of 66_10

100 mg of **66** in 1 ml DMF was activated by 1 eq (206 mg) of PyBOP at RT for 20 min under argon atmosphere. Activated **66** was added to 2,2′-(Ethylenedioxy) bis(ethylamine) (5 eq,293 mg) dissolved in 0.5 ml DMF while stirring. The reaction was stirred under argon protection for 3 h at RT. A small amount of reaction crude (2 μmol) was injected in UPLC-ESI-MS to evaluate the reaction yield. When the reaction product was detected, a two-fold volume of water was added to the reaction, and the crude was freeze-dried. The dried reaction crude was then dissolved in 2 ml DMSO and was purified by reverse-phase HPLC to obtain the reaction product. HPLC fractions were injected in UPLC-ESI-MS to identify the best fraction, and the desired fractions were freeze-dried yielding 89.3 mg (59%) of **66_10**.

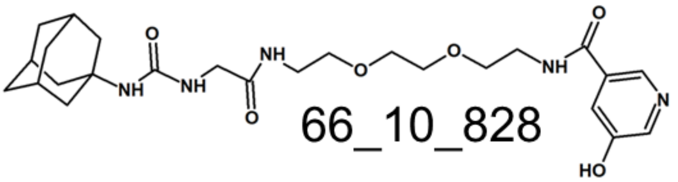

Synthesis of 66_10_828

5 mg of **828** was dissolved in 0.6 ml of DMF and was activated by HATU (1 eq, 13.7 mg in 100 µl DMF) and HOAt (1 eq, 4.9 mg in 100 µl) under argon protection for 20 min at RT. To the activated **828** was added 13.7 mg of **66_10** in 0.5 ml DMF. The reaction was stirred under argon protection for 3 h at RT. A small amount of reaction crude (2 µmol) was injected in UPLC-ESI-MS to evaluate the reaction yield. When the reaction product was detected, a two-fold volume of water was added to the reaction and the crude was freeze-dried yielding 5.8 mg (32%) of **66_10_828**. 1H NMR (600 MHz, DMSO-d6) δ 10.41 (s, 1H), 8.68 (t, J = 1.9 Hz, 1H), 8.45 (d, J = 10.3 Hz, 1H), 8.45 (s, 1H), 8.33 (t, J = 1.9 Hz, 1H), 7.81 (d, J = 9.6 Hz, 1H), 7.81 (s, 1H), 7.64 (t, J = 2.0 Hz, 1H), 6.11 (t, J = 5.3 Hz, 1H), 5.45 (s, 1H), 3.73 (d, J = 5.3 Hz, 2H), 3.59 (t, J = 4.1 Hz, 2H), 3.55 (t, J = 4.4 Hz, 2H), 3.46 (dt, J = 5.1, 4.1 Hz, 2H), 3.29 (q, J = 4.4 Hz, 2H), 2.05 (hept, J = 5.3 Hz, 3H), 1.95 (d, J = 5.2 Hz, 6H), 1.63 (t, J = 5.4 Hz, 6H).

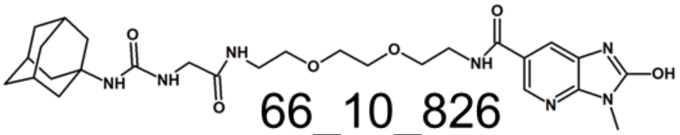

Synthesis of 66_10_826

4 mg of **826** was dissolved in 0.7 ml of DMF and was activated by HATU (1 eq, 9.8 mg in 100 µl DMF) and HOAt (1 eq, 3.5 mg in 100 µl) under argon protection for 20 min at RT. To the activated **826** was added 7.9 mg of **66_10** in 0.5 ml DMF. The reaction was stirred under argon protection for 3 h at RT. A small amount of reaction crude (2 µmol) was injected in UPLC-ESI-MS to evaluate the reaction yield. When the reaction product was detected, a two-fold volume of water was added to the reaction and the crude was freeze-dried yielding 4.2 mg (36%) of **66_10_826**. 1H NMR (600 MHz, DMSO-d6) δ 10.33 (s, 1H), 8.80 (d, J = 1.5 Hz, 2H), 8.44 (t, J = 5.3 Hz, 2H), 8.24 (d, J = 1.5 Hz, 2H), 7.81 (d, J = 9.6 Hz, 1H), 7.81 (s, 1H), 6.11 (t, J = 5.3 Hz, 2H), 5.46 (s, 1H), 3.73 (d, J = 5.0 Hz, 10H), 3.59 (t, J = 4.1 Hz, 4H), 3.55 (t, J = 4.4 Hz, 4H), 3.46 (dt, J = 5.1, 4.1 Hz, 4H), 3.29 (q, J = 4.4 Hz, 4H), 2.05 (hept, J = 5.3 Hz, 6H), 1.95 (d, J = 5.2 Hz, 12H), 1.65 (t, J = 5.4 Hz, 11H).

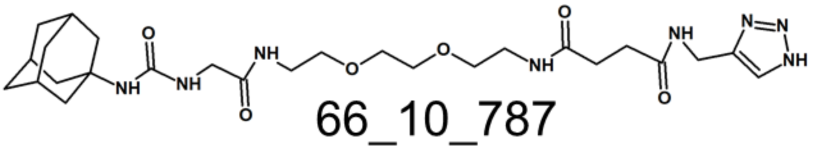

Synthesis of 66_10_787

10 mg of **66_10** was dissolved in 0.3 ml DMSO. To the solution was added 4 mg of succinic anhydride in 0.3 ml DMSO. The reaction was stirred for 30 min at RT and the product was validated by UPLC-ESI-MS. The product was activated by 1 eq (15.2 mg) HATU and 1 eq (5.4 mg) HOAt RT for 20 min under argon atmosphere. 1.5 eq (5.9 mg) of **787** was dissolved 0.3 ml DMSO and added to the crude. When the reaction product was detected, a two-fold volume of water was added to the reaction and the crude was freeze-dried. The dried reaction crude was then dissolved in 0.2 ml DMSO and was purified by reverse-phase HPLC to obtain the reaction product. HPLC fractions were injected in UPLC-ESI-MS to identify the best fraction, and the desired fractions were freeze-dried yielding 5.6 mg (25%) of **66_10_787**. 1H NMR (600 MHz, DMSO-d6) δ 12.58 (d, J = 1.5 Hz, 1H), 8.36 (t, J = 6.1 Hz, 1H), 8.07 (d, J = 1.2 Hz, 1H), 7.44 (t, J = 5.0 Hz, 1H), 6.11 (t, J = 5.3 Hz, 1H), 5.46 (s, 0H), 4.33 (d, J = 5.9 Hz, 1H), 3.73 (d, J = 5.3 Hz, 1H), 3.60 (s, 2H), 3.55 (q, J = 4.3 Hz, 3H), 3.35 – 3.27 (m, 3H), 2.42 – 2.33 (m, 3H), 2.05 (hept, J = 5.3 Hz, 2H), 1.95 (d, J = 5.2 Hz, 4H), 1.65 (t, J = 5.4 Hz, 4H).

Synthesis of fragment pairs linked by linker 12

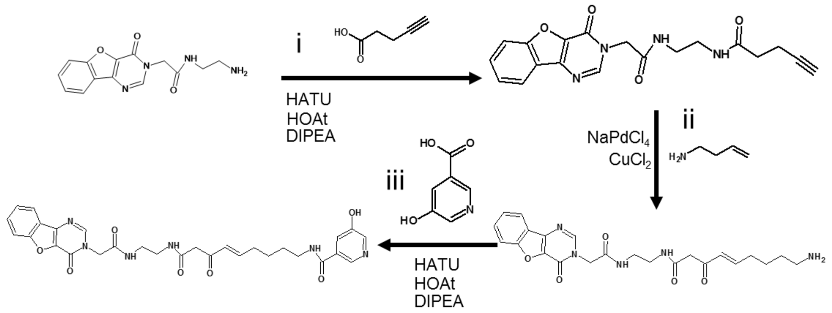

Synthesis of 182_12_828

i) 30 mg of pentynoic acid was dissolved in 0.3 ml of DMF and was activated by 1 eq (116 mg) HATU and 1 eq (41 mg) HOAt at RT for 20 min under argon atmosphere. To the activation solution, 1 eq 90 mg of **182_1** in 1 ml DMF was added after tuning the pH to 8 by DIPEA. The reaction was stirred at RT for 3 hours to give rise to *N*-{2-[2-(4-oxo[1]benzofuro[3,2-*d*]pyrimidin-3(4*H*)-yl)acetamido]ethyl}pent-4-ynamide. The reaction was monitored by UPLC-ESI-MS and purified by HPLC. HPLC fractions containing the product was subjected to freeze-drying. ii) The synthesis of α,ß-unsaturated ketone between alkynamide and alkene was performed according to a previous report by David R. Liu group[2]. 20 mg (1.5 eq) of 1-Amino-3-buten-hydrochloride was dissolved in 1 ml ACN: H_2O (5:1) solution. To the solution was

added 15 mol% Na_2PdCl_4 and 20 mol% $CuCl_2$. 1 eq of *N*-{2-[2-(4-oxo[1]benzofuro[3,2-*d*]pyrimidin-3(4*H*)-yl)acetamido]ethyl}pent-4-ynamide was dissolved in DMSO and was added to the mixture dropwise over 8 h. The crude was then freeze-dried, dissolved in DMSO for HPLC purification. The purified fractions were injected in UPLC-ESI-MS for validation. Fractions containing reaction product were collected and freeze-dried. iii) To generate 182_12_828, 1 mg of **828** was dissolved in 0.6 ml of DMF and was activated by HATU (1 eq, 2.7 mg in 100 µl DMF) and HOAt (1 eq, 0.2 mg in 100 µl) under argon protection for 20 min at RT. To the activated **828** was added 1 eq of the reaction product from step ii, dissolved in 0.5 ml DMF. The reaction was stirred under argon protection for 3 h at RT. A small amount of reaction crude (2 µmol) was injected in UPLC-ESI-MS to evaluate the reaction yield. When the reaction product was detected, a two-fold volume of water was added to the reaction, and the crude was freeze-dried. The dried reaction crude was then dissolved in DMSO and was purified by reverse-phase HPLC to obtain the reaction product. HPLC fractions were injected in UPLC-ESI-MS to identify the best fraction, and the desired fractions were freeze-dried yielding 2.1 mg (3.6%) of **182_12_828**. 1H NMR (600 MHz, DMSO-d6) δ 10.41 (s, 1H), 9.00 (s, 1H), 8.66 (t, J = 1.7 Hz, 1H), 8.36 (t, J = 5.0 Hz, 1H), 8.33 (t, J = 1.9 Hz, 1H), 8.09 (dd, J = 8.5, 1.3 Hz, 1H), 7.96 (td, J = 5.2, 1.2 Hz, 2H), 7.67 – 7.61 (m, 2H), 7.55 (ddd, J = 8.7, 6.7, 1.2 Hz, 1H), 7.44 (td, J = 8.7, 1.4 Hz, 1H), 6.67 (dt, J = 15.3, 5.9 Hz, 1H), 6.19 (dt, J = 15.2, 1.0 Hz, 1H), 4.53 (s, 1H), 3.34 – 3.29 (m, 2H), 3.30 – 3.23 (m, 4H), 2.25 – 2.18 (m, 2H), 1.63 (tt, J = 8.0, 5.0 Hz, 2H), 1.53 – 1.44 (m, 2H).

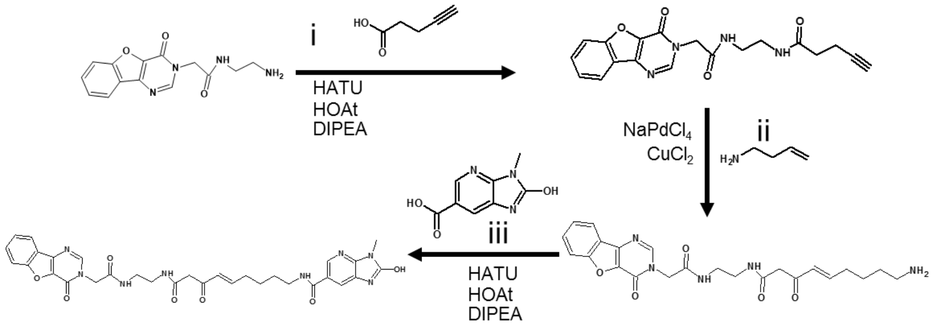

Synthesis of 182_12_826

i) 30 mg of pentynoic acid was dissolved in 0.3 ml of DMF and was activated by 1 eq (116 mg) HATU and 1 eq (41 mg) HOAt at RT for 20 min under argon atmosphere. To the activation solution, 1 eq 90 mg of 182_1 in 1 ml DMF was added after tuning the pH to 8 by DIPEA. The reaction was stirred at RT for 3 hours to give rise to *N*-{2-[2-(4-oxo[1]benzofuro[3,2-*d*]pyrimidin-3(4*H*)-yl)acetamido]ethyl}pent-4-ynamide. The reaction was monitored by UPLC-ESI-MS and purified by HPLC. HPLC fractions containing the product was subjected to freeze-drying. ii) The synthesis of α,ß-unsaturated ketone between alkynamide and alkene was performed according to a previous report by David R. Liu group[2]. 20 mg (1.5 eq) of 1-Amino-

3-buten-hydrochloride was dissolved in 1 ml ACN: H_2O (5:1) solution. To the solution, 15 mol% Na_2PdCl_4 and 20 mol% $CuCl_2$ were added. 1 eq of *N*-{2-[2-(4-oxo[1]benzofuro[3,2-*d*]pyrimidin-3(4*H*)-yl)acetamido]ethyl}pent-4-ynamide was dissolved in DMSO and was added to the mixture dropwise over 8 h. The crude was then freeze-dried, dissolved in DMSO for HPLC purification. The purified fractions were injected in UPLC-ESI-MS for validation. Fractions containing reaction product was collected and freeze-dried. iii) To generate **182_12_826**, 2 mg of **826** was dissolved in 0.5 ml DMF and was activated by 1 eq (4 mg) HATU and 1 eq (1.4 mg) HOAt at RT for 20 min under argon atmosphere. To the activated **826** was added 1 eq of the reaction product from step ii, dissolved in 0.5 ml DMF. The reaction was stirred under argon protection for 3 h at RT. A small amount of reaction crude (2 µmol) was injected in UPLC-ESI-MS to evaluate the reaction yield. When the reaction product was detected, a two-fold volume of water was added to the reaction, and the crude was freeze-dried. The dried reaction crude was then dissolved in DMSO and was purified by reverse-phase HPLC to obtain the reaction product. HPLC fractions were injected in UPLC-ESI-MS to identify the best fraction, and the desired fractions were freeze-dried yielding 1.7 mg (1.3%) of **182_12_826**. 1H NMR (600 MHz, DMSO-d6) δ 10.33 (s, 1H), 9.00 (s, 1H), 8.80 (d, J = 1.5 Hz, 1H), 8.38 (t, J = 5.0 Hz, 1H), 8.24 (d, J = 1.5 Hz, 1H), 8.10 (dd, J = 8.6, 1.3 Hz, 1H), 7.95 (td, J = 5.2, 1.2 Hz, 2H), 7.64 (dd, J = 6.6, 1.3 Hz, 1H), 7.55 (ddd, J = 8.7, 6.7, 1.2 Hz, 1H), 7.44 (td, J = 8.7, 1.4 Hz, 1H), 6.68 (dt, J = 15.3, 5.9 Hz, 1H), 6.19 (dt, J = 15.2, 0.9 Hz, 1H), 4.53 (s, 1H), 3.73 (s, 2H), 3.39 (s, 1H), 3.34 – 3.29 (m, 2H), 3.30 – 3.24 (m, 4H), 2.25 – 2.18 (m, 2H), 1.63 (tt, J = 7.9, 4.9 Hz, 2H), 1.53 – 1.44 (m, 2H).

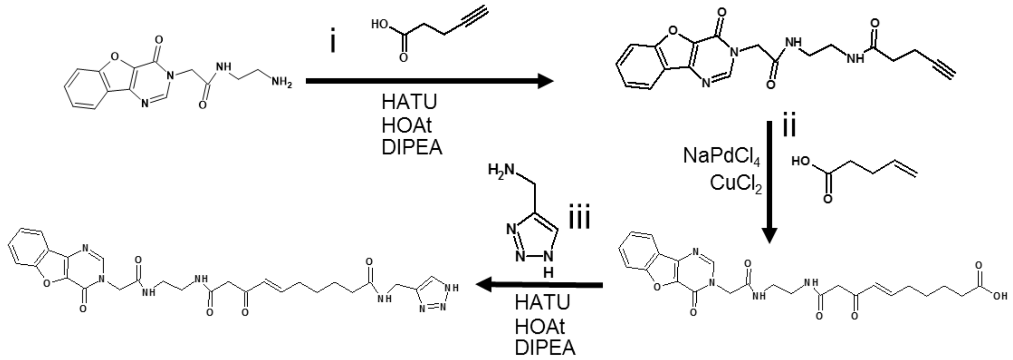

Synthesis of 182_12_787

i) 30 mg of pentynoic acid was dissolved in 0.3 ml of DMF and was activated by 1 eq HATU (116 mg) and 1 eq (41 mg) HOAt at RT for 20 min under argon atmosphere. To the activation solution, 1 eq 90 mg of 182_1 in 1 ml DMF was added after tuning the pH to 8 by DIPEA. The reaction was stirred at RT for 3 hours to give rise to *N*-{2-[2-(4-oxo[1]benzofuro[3,2-*d*]pyrimidin-3(4*H*)-yl)acetamido]ethyl}pent-4-ynamide. The reaction was monitored by UPLC-ESI-MS and purified by HPLC. HPLC fractions containing the product was subjected to freeze-drying. ii) The synthesis of α,ß-unsaturated ketone between alkynamide and alkene was

performed according to a previous report by David R. Liu group[2]. 20 mg (1.5 eq) of 4-pentenoic acid was dissolved in 1 ml ACN: H_2O (5:1) solution. To the solution, 15 mol% Na_2PdCl_4 and 20 mol% $CuCl_2$ was added. 1 eq of *N*-{2-[2-(4-oxo[1]benzofuro[3,2-*d*]pyrimidin-3(4*H*)-yl)acetamido]ethyl}pent-4-ynamide was dissolved in DMSO and was added to the mixture dropwise over 8 h. The crude was then freeze-dried dissolved in DMSO for HPLC purification. The purified fractions were injected in UPLC-ESI-MS for validation. Fractions containing the reaction product were collected and freeze-dried. iii) 24 mg of the reaction product from step ii was dissolved in 0.5 mM DMF and was activated by 1 eq HATU and 1 eq HOAt at RT for 20 min under argon atmosphere. After activation, 8 mg of **787** in 0.4 ml DMF whose pH was tuned to 8 by DIPEA was added to the solution. The reaction was stirred for 3 h at RT. A small amount of reaction crude (2 µmol) was injected in UPLC-ESI-MS to evaluate the reaction yield. When the reaction product was detected, a two-fold volume of water was added to the reaction, and the crude was freeze-dried. The dried reaction crude was then dissolved in DMSO and was purified by reverse-phase HPLC to obtain the reaction product. HPLC fractions were injected in UPLC-ESI-MS to identify the best fraction, and the desired fractions were freeze-dried yielding 1.8 mg (1.1%) of **182_12_787**. 1H NMR (600 MHz, DMSO-d6) δ 12.58 (d, J = 1.5 Hz, 1H), 9.00 (s, 1H), 8.26 (t, J = 6.1 Hz, 1H), 8.10 (dd, J = 8.5, 1.3 Hz, 1H), 8.07 (d, J = 1.2 Hz, 1H), 7.95 (td, J = 5.2, 1.2 Hz, 2H), 7.64 (dd, J = 6.6, 1.3 Hz, 1H), 7.55 (ddd, J = 8.7, 6.7, 1.3 Hz, 1H), 7.44 (td, J = 8.7, 1.4 Hz, 1H), 6.67 (dt, J = 15.3, 5.9 Hz, 1H), 6.19 (dt, J = 15.2, 0.9 Hz, 1H), 4.54 (s, 1H), 4.33 (d, J = 5.9 Hz, 2H), 3.35 – 3.23 (m, 4H), 2.27 – 2.18 (m, 4H), 1.62 – 1.54 (m, 2H), 1.52 – 1.44 (m, 2H).

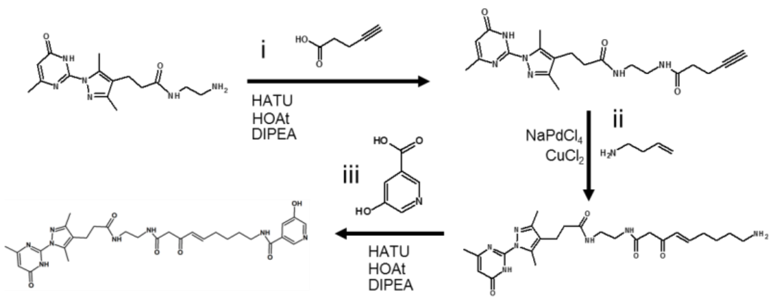

Synthesis of 693_12_828

i) 30 mg of pentynoic acid was dissolved in 0.3 ml of DMF and was activated by 1 eq HATU (116 mg) and 1 eq (41 mg) HOAt at RT for 20 min under argon atmosphere. To the activation solution, 1 eq 9 mg of **693_1** in 1 ml DMF was added after tuning the pH to 8 by DIPEA. The reaction was stirred at RT for 3 hours to give rise to *N*-{2-[2-(4-oxo[1]benzofuro[3,2-*d*]pyrimidin-3(4*H*)-yl)acetamido]ethyl}pent-4-ynamide. The reaction was monitored by UPLC-

ESI-MS and purified by HPLC. HPLC fractions containing the product was subjected to freeze-drying. ii) The synthesis of α,ß-unsaturated ketone between alkynamide and alkene was performed according to a previous report by David R. Liu group[2]. 20 mg (1.5 eq) of 1-Amino-3-buten-hydrochloride was dissolved in 1 ml ACN: H_2O (5:1) solution. To the solution, 15 mol% Na_2PdCl_4 and 20 mol% $CuCl_2$ was added. 1 eq alkynamide was dissolved in DMSO and was added to the mixture dropwise over 8 h. The crude was then freeze-dried and dissolved in DMSO for HPLC purification. The purified fractions were injected in UPLC-ESI-MS for validation. Fractions containing reaction product was collected and freeze-dried. Iii) To generate **693_12_828**, 3 mg of **828** was dissolved in 0.5 ml DMF and was activated by 1 eq (8.2 mg) HATU and 1 eq (2.9 mg) HOAt at RT for 20 min under argon atmosphere. To the activated 828, 1 eq of the reaction product from step ii was added, dissolved in 0.5 ml DMF. The reaction was stirred under argon protection for 3 h at RT. A small amount of reaction crude (2 µmol) was injected in UPLC-ESI-MS to evaluate the reaction yield. When the reaction product was detected, a two-fold volume of water was added to the reaction and the crude was freeze-dried. The dried reaction crude was then dissolved in DMSO and was purified by reverse-phase HPLC to obtain the reaction product. HPLC fractions were injected in UPLC-ESI-MS to identify the best fraction, and the desired fractions were freeze-dried yielding 1.9 mg (1%) of **693_12_828**. 1H NMR (600 MHz, DMSO-d6) δ 10.54 (s, 1H), 10.41 (s, 1H), 8.66 (t, J = 1.7 Hz, 1H), 8.36 (t, J = 5.0 Hz, 1H), 8.32 (t, J = 1.9 Hz, 1H), 7.98 (t, J = 5.1 Hz, 1H), 7.68 – 7.61 (m, 2H), 6.68 (dt, J = 15.3, 5.9 Hz, 1H), 6.22 – 6.16 (m, 1H), 5.82 (q, J = 1.2 Hz, 1H), 3.34 – 3.29 (m, 2H), 3.26 (qd, J = 4.7, 1.3 Hz, 4H), 2.88 (dt, J = 17.7, 8.0 Hz, 1H), 2.81 – 2.70 (m, 1H), 2.51 – 2.45 (m, 5H), 2.44 (s, 2H), 2.25 – 2.18 (m, 2H), 2.17 (s, 2H), 1.63 (tt, J = 7.9, 4.9 Hz, 2H), 1.53 – 1.45 (m, 2H).

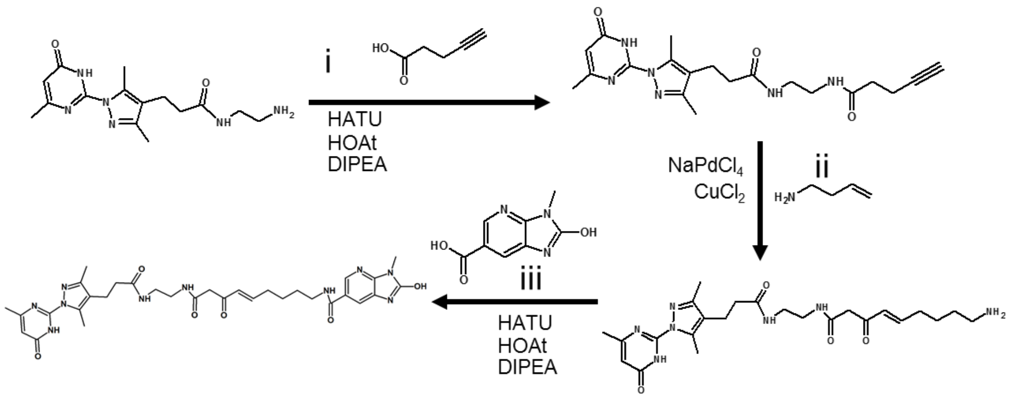

Synthesis of 693_12_826

i) 30 mg of pentynoic acid was dissolved in 0.3 ml of DMF and was activated by 1 eq HATU (116 mg) and 1 eq (41 mg) HOAt at RT for 20 min under argon atmosphere. To the activation

solution, 1 eq 97 mg of 693_1 in 1 ml DMF was added after tuning the pH to 8 by DIPEA. The reaction was monitored by UPLC-ESI-MS and purified by HPLC. HPLC fractions containing the product was subjected to freeze drying. ii) The synthesis of α,ß-unsaturated ketone between alkynamide and alkene was performed according to a previous report by David R. Liu group[2]. 20 mg (1.5 eq) of 1-Amino-3-buten-hydrochloride was dissolved in 1 ml ACN: H2O (5:1) solution. To the solution was added 15 mol% Na_2PdCl_4 and 20 mol% $CuCl_2$. 1 eq alkynamide was dissolved in DMSO and was added to the mixture dropwise over 8 h. The crude was then freeze-dried, dissolved in DMSO for HPLC purification. The purified fractions were injected in UPLC-ESI-MS for validation. Fractions containing reaction product was collected and freeze-dried. iii) To generate **693_12_826**, 2 mg of **826** was dissolved in 0.5 ml DMF and was activated by 1 eq (4 mg) HATU and 1 eq (1.4 mg) HOAt at RT for 20 min under argon atmosphere. To the activated **826** was added 1 eq of the reaction product from step ii, dissolved in 0.5 ml DMF. The reaction was stirred under argon protection for 3 h at RT. A small amount of reaction crude (2 µmol) was injected in UPLC-ESI-MS to evaluate the reaction yield. When the reaction product was detected, a two-fold volume of water was added to the reaction, and the crude was freeze-dried. The dried reaction crude was then dissolved in DMSO and was purified by reverse-phase HPLC to obtain the reaction product. HPLC fractions were injected in UPLC-ESI-MS to identify the best fraction, and the desired fractions were freeze-dried yielding 1.9 mg (0.97%) of **693_12_826**. 1H NMR (600 MHz, DMSO-d6) δ 10.54 (s, 1H), 10.33 (s, 1H), 8.80 (d, J = 1.5 Hz, 1H), 8.38 (t, J = 5.0 Hz, 1H), 8.24 (d, J = 1.5 Hz, 1H), 7.98 (t, J = 5.1 Hz, 1H), 7.65 (t, J = 5.1 Hz, 1H), 6.67 (dt, J = 15.3, 5.9 Hz, 1H), 6.19 (dt, J = 15.2, 0.9 Hz, 1H), 5.82 (q, J = 1.2 Hz, 1H), 3.73 (s, 2H), 3.34 – 3.29 (m, 2H), 3.29 – 3.23 (m, 4H), 2.88 (dt, J = 17.7, 8.0 Hz, 1H), 2.80 – 2.71 (m, 1H), 2.51 – 2.45 (m, 5H), 2.44 (s, 2H), 2.25 – 2.18 (m, 2H), 2.17 (s, 2H), 1.63 (tt, J = 7.9, 4.9 Hz, 2H), 1.53 – 1.44 (m, 2H).

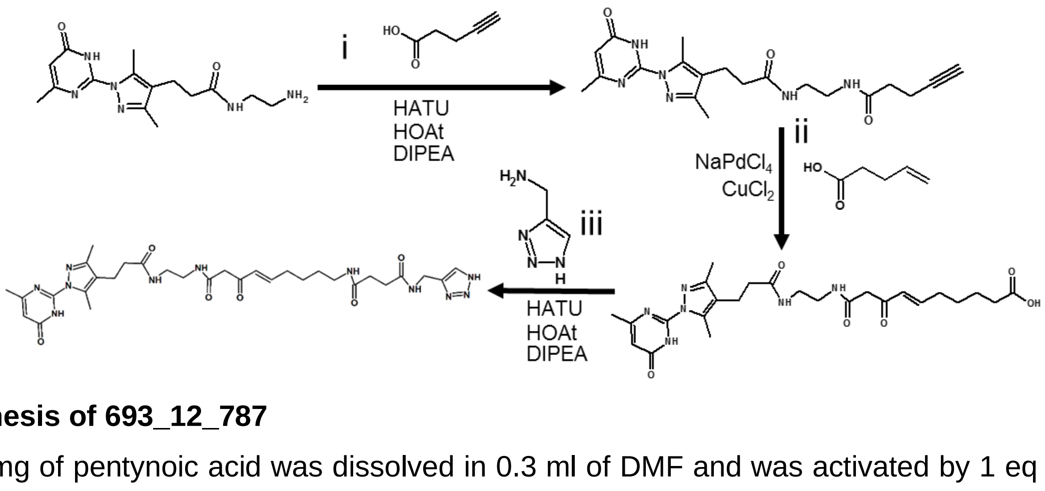

Synthesis of 693_12_787

i) 30 mg of pentynoic acid was dissolved in 0.3 ml of DMF and was activated by 1 eq HATU (116 mg) and 1 eq (41 mg) HOAt at RT for 20 min under argon atmosphere. To the activation solution, 1 eq 90 mg of 182_1 in 1 ml DMF was added after tuning the pH to 8 by DIPEA. The

reaction was monitored by UPLC-ESI-MS and purified by HPLC. HPLC fractions containing the product was subjected to freeze-drying. ii) The synthesis of α,ß-unsaturated ketone between alkynamide and alkene was performed according to a previous report by David R. Liu group[2]. 20 mg (1.5 eq) of 4-pentenoic acid was dissolved in 1 ml ACN: H_2O (5:1) solution. To the solution, 15 mol% Na_2PdCl_4 and 20 mol% $CuCl_2$ was added. 1 eq of alkynamide was dissolved in DMSO and was added to the mixture dropwise over 8 h. The crude was then freeze-dried, dissolved in DMSO for HPLC purification. The purified fractions were injected in UPLC-ESI-MS for validation. Fractions containing the reaction product was collected and freeze-dried. iii) 4 mg of the reaction product from step ii was dissolved in 0.5 mM DMF and was activated by 1 eq HATU and 1 eq HOAt at RT for 20 min under argon atmosphere. After activation, 1.6 mg of **787** in 0.4 ml DMF, whose pH was tuned to 8 by DIPEA, was added to the solution. The reaction was stirred for 3 h at RT. A small amount of reaction crude (2 µmol) was injected in UPLC-ESI-MS to evaluate the reaction yield. When the reaction product was detected, a two-fold volume of water was added to the reaction, and the crude was freeze-dried. The dried reaction crude was then dissolved in DMSO and was purified by reverse-phase HPLC to obtain the reaction product. HPLC fractions were injected in UPLC-ESI-MS to identify the best fraction, and the desired fractions were freeze-dried yielding 1.8 mg (0.9%) of **693_12_787**. 1H NMR (600 MHz, DMSO-d6) δ 12.58 (d, J = 1.5 Hz, 1H), 10.54 (s, 1H), 8.38 (t, J = 6.1 Hz, 1H), 8.07 (d, J = 1.2 Hz, 1H), 7.98 (t, J = 5.1 Hz, 1H), 7.65 (t, J = 5.1 Hz, 1H), 7.61 (t, J = 5.0 Hz, 1H), 6.68 (dt, J = 15.3, 5.9 Hz, 1H), 6.22 – 6.16 (m, 1H), 5.81 (q, J = 1.2 Hz, 1H), 4.33 (d, J = 5.9 Hz, 2H), 3.34 – 3.29 (m, 2H), 3.29 – 3.23 (m, 2H), 3.12 (q, J = 5.1 Hz, 2H), 2.88 (dt, J = 17.7, 8.0 Hz, 1H), 2.80 – 2.71 (m, 1H), 2.51 – 2.43 (m, 6H), 2.43 – 2.33 (m, 4H), 2.25 – 2.18 (m, 2H), 2.17 (s, 2H), 1.58 – 1.44 (m, 4H).

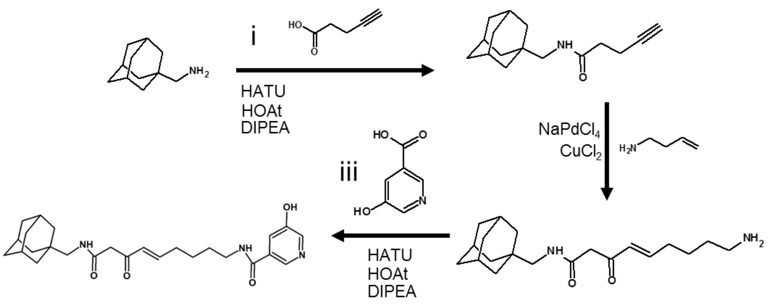

Synthesis of 66_12_828

i) 30 mg of pentynoic acid was dissolved in 0.3 ml of DMF and was activated by 1 eq HATU (116 mg) and 1 eq (41 mg) HOAt at RT for 20 min under argon atmosphere. To the activation solution, 1 eq (77 mg) of 1-adamantane methyl amine in 1 ml DMF was added after tuning the

pH to 8 by DIPEA. The reaction was monitored by UPLC-ESI-MS and purified by HPLC. HPLC fractions containing the product was subjected to freeze-drying. ii) The synthesis of α,ß-unsaturated ketone between alkynamide and alkene was performed according to a previous report by David R. Liu group[2]. 20 mg (1.5 eq) of 1-Amino-3-buten-hydrochloride was dissolved in 1 ml ACN: H_2O (5:1) solution. To the solution, 15 mol% Na_2PdCl_4 and 20 mol% $CuCl_2$ was added. 1 eq alkynamide was dissolved in DMSO and was added to the mixture dropwise over 8 h. The crude was then freeze-dried, dissolved in DMSO for HPLC purification. The purified fractions were injected in UPLC-ESI-MS for validation. Fractions containing reaction product was collected and freeze-dried. iii) To generate **66_12_828**, 3 mg of **828** was dissolved in 0.5 ml DMF and was activated by 1 eq (8.2 mg) HATU and 1 eq (2.9 mg) HOAt at RT for 20 min under argon atmosphere. To the activated **828** was added 1 eq of the reaction product from step ii, dissolved in 0.5 ml DMF. The reaction was stirred under argon protection for 3 h at RT. A small amount of reaction crude (2 μmol) was injected in UPLC-ESI-MS to evaluate the reaction yield. When the reaction product was detected, a two-fold volume of water was added to the reaction, and the crude was freeze-dried. The dried reaction crude was then dissolved in DMSO and was purified by reverse-phase HPLC to obtain the reaction product. HPLC fractions were injected in UPLC-ESI-MS to identify the best fraction, and the desired fractions were freeze-dried yielding 1.7 mg (1.2%) **66_12_828**. 1H NMR (600 MHz, DMSO-d6) δ 10.41 (s, 1H), 8.65 (t, J = 1.7 Hz, 1H), 8.36 (t, J = 5.0 Hz, 1H), 8.33 (t, J = 1.9 Hz, 1H), 7.63 (t, J = 2.0 Hz, 1H), 7.51 (t, J = 6.1 Hz, 1H), 6.69 (dt, J = 15.3, 5.9 Hz, 1H), 6.22 – 6.16 (m, 1H), 3.26 (q, J = 5.0 Hz, 2H), 3.06 (d, J = 5.9 Hz, 2H), 2.25 – 2.18 (m, 2H), 2.03 – 1.95 (m, 3H), 1.67 – 1.60 (m, 8H), 1.53 – 1.44 (m, 8H).

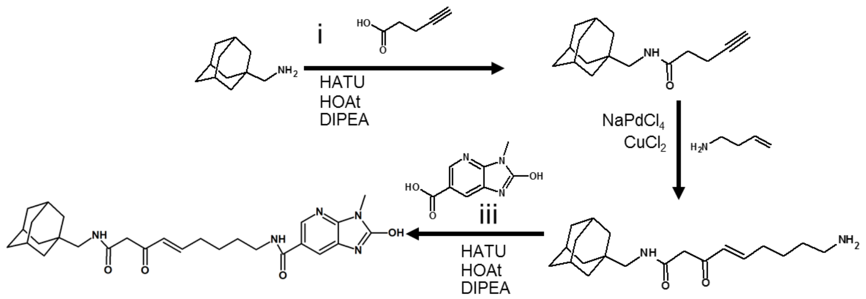

Synthesis of 66_12_826

i) 30 mg of pentynoic acid was dissolved in 0.3 ml of DMF and was activated by 1 eq HATU (116 mg) and 1 eq (41 mg) HOAt at RT for 20 min under argon atmosphere. To the activation solution, 1 eq (77 mg) of 1-adamantane methyl amine in 1 ml DMF was added after tuning the pH to 8 by DIPEA. The reaction was monitored by UPLC-ESI-MS and purified by HPLC. HPLC fractions containing the product was subjected to freeze-drying. ii) The synthesis of α,ß-unsaturated ketone between alkynamide and alkene was performed according to a previous

report by David R. Liu group[2]. 20 mg (1.5 eq) of 1-Amino-3-buten-hydrochloride was dissolved in 1 ml ACN: H_2O (5:1) solution. To the solution, 15 mol% Na_2PdCl_4 and 20 mol% $CuCl_2$ was added. 1 eq alkynamide was dissolved in DMSO and was added to the mixture dropwise over 8 h. The crude was then freeze-dried, dissolved in DMSO for HPLC purification. The purified fractions were injected in UPLC-ESI-MS for validation. Fractions containing reaction product was collected and freeze-dried. iii) To generate **66_12_826**, 2 mg of **826** was dissolved in 0.5 ml DMF and was activated by 1 eq (4 mg) HATU and 1 eq (1.4 mg) HOAt at RT for 20 min under argon atmosphere. To the activated 826 was added 1 eq of the reaction product from step ii, dissolved in 0.5 ml DMF. The reaction was stirred under argon protection for 3 h at RT. A small amount of reaction crude (2 µmol) was injected in UPLC-ESI-MS to evaluate the reaction yield. When the reaction product was detected, a two-fold volume of water was added to the reaction and the crude was freeze-dried. The dried reaction crude was then dissolved in DMSO and was purified by reverse-phase HPLC to obtain the reaction product. HPLC fractions were injected in UPLC-ESI-MS to identify the best fraction, and the desired fractions were freeze-dried yielding 1.7 mg (1.1%) of **66_12_826**. 1H NMR (600 MHz, DMSO-d6) δ 10.33 (s, 1H), 8.80 (d, J = 1.5 Hz, 1H), 8.36 (t, J = 5.0 Hz, 1H), 8.24 (d, J = 1.5 Hz, 1H), 7.50 (t, J = 6.1 Hz, 1H), 6.68 (dt, J = 15.3, 5.9 Hz, 1H), 6.22 – 6.17 (m, 1H), 3.72 (s, 2H), 3.26 (q, J = 5.0 Hz, 2H), 3.06 (d, J = 5.9 Hz, 2H), 2.27 – 2.17 (m, 2H), 2.03 – 1.95 (m, 3H), 1.67 – 1.60 (m, 8H), 1.53 – 1.45 (m, 8H).

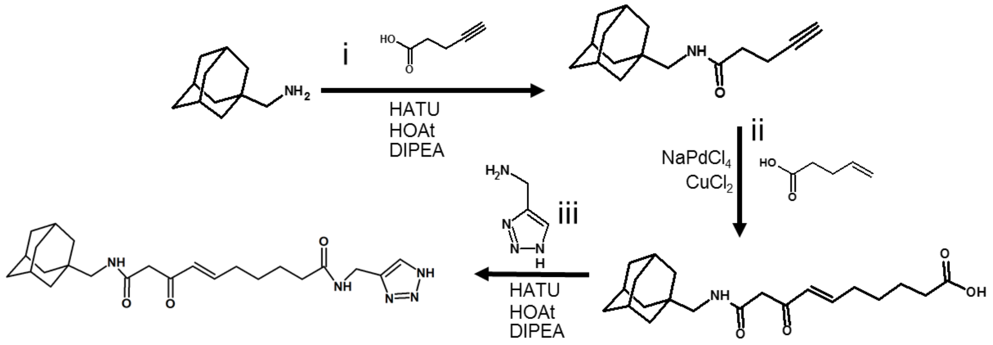

Synthesis of 66_12_787

i) 30 mg of pentynoic acid was dissolved in 0.3 ml of DMF and was activated by 1 eq HATU (116 mg) and 1 eq (41 mg) HOAt at RT for 20 min under argon atmosphere. To the activation solution, 1 eq (77 mg) of 1-adamantane methyl amine in 1 ml DMF was added after tuning the pH to 8 by DIPEA. The reaction was monitored by UPLC-ESI-MS and purified by HPLC. HPLC fractions containing the product was subjected to freeze-drying. ii) The synthesis of α,ß-unsaturated ketone between alkynamide and alkene was performed according to a previous report by David R. Liu group[2]. 20 mg (1.5 eq) of 4-pentenoic acid was dissolved in 1 ml ACN: H2O (5:1) solution. To the solution, 15 mol% Na_2PdCl_4 and 20 mol% $CuCl_2$ was added. 1 eq of alkynamide was dissolved in DMSO and was added to the mixture dropwise over 8 h. The

crude was then freeze-dried dissolved in DMSO for HPLC purification. The purified fractions were injected in UPLC-ESI-MS for validation. Fractions containing the reaction product was collected and freeze-dried. iii) 24 mg of the reaction product from step ii was dissolved in 0.5 mM DMF and was activated by 1 eq HATU and 1 eq HOAt at RT for 20 min under argon atmosphere. After activation, 2 mg of **787** in 0.4 ml DMF whose pH was tuned to 8 by DIPEA was added to the solution. The reaction was stirred for 3 h at RT. A small amount of reaction crude (2 μmol) was injected in UPLC-ESI-MS to evaluate the reaction yield. When the reaction product was detected, a two-fold volume of water was added to the reaction and the crude was freeze-dried. The dried reaction crude was then dissolved in DMSO and was purified by reverse-phase HPLC to obtain reaction product. HPLC fractions were injected in UPLC-ESI-MS to identify the best fraction, and the desired fractions were freeze-dried yielding 2.1 mg (1.1%) of **66_12_787**. 1H NMR (600 MHz, DMSO-d6) δ 12.58 (d, J = 1.5 Hz, 1H), 8.27 (t, J = 6.1 Hz, 1H), 8.06 (d, J = 1.2 Hz, 1H), 7.50 (t, J = 6.1 Hz, 1H), 6.67 (dt, J = 15.3, 5.9 Hz, 1H), 6.22 – 6.16 (m, 1H), 4.33 (d, J = 5.9 Hz, 2H), 3.05 (d, J = 5.9 Hz, 2H), 2.27 – 2.18 (m, 4H), 2.03 – 1.95 (m, 3H), 1.64 (t, J = 5.8 Hz, 5H), 1.62 – 1.54 (m, 2H), 1.53 – 1.43 (m, 8H).

Synthesis of fragment pairs linker by 24.

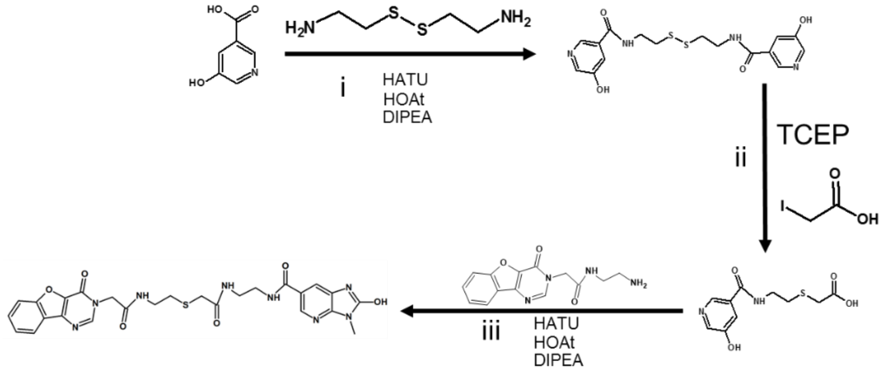

Synthesis of 182_24_828

i) 50 mg of **828** was dissolved in 3 ml DMSO and was activated by 1 eq (136 mg) HATU and 1 eq (49 mg) HOAt at RT for 20 min under argon atmosphere. 0.5 eq (40 mg) of cystamine dihydrochloride was dissolved in 2 ml of DMSO and pH was tuned to 8 by DIPEA and then added to the activated **828** drop by drop. The reaction was stirred for 3 h at RT. Small amount of reaction crude (2 μmol) was injected in UPLC-ESI-MS to evaluate the reaction yield. When the reaction product was detected, two-fold volume of water was added to the reaction and the crude was freeze-dried. The dried reaction crude was then dissolved in DMSO and was purified by reverse-phase HPLC to obtain the reaction product. HPLC fractions were injected in UPLC-ESI-MS to identify the best fraction, and the desired fractions were freeze-dried. ii) 10 mg of the reaction product was dissolved in 0.3 ml DMSO and was mixed with 2 eq of

TCEP buffer (100 mM aqueous buffer, pH 7.5) and 3 eq (28.3 mg) of iodoacetic acid dissolved in 0.5 ml DMSO. The reaction was stirred at 37 ºC for 1h. The crude was freeze-dried and purified by HPLC. Fractions were validated by UPLC-ESI-MS and fractions containing the products were freeze-dried. iii) The resulting carboxylic acid was activated by 1 eq HATU and 1 eq HOAt at RT for 20 min under argon atmosphere. 1 eq (5 mg) of **182_1** was added to the reaction. The reaction was stirred for 3 h at RT. A small amount of reaction crude (2 µmol) was injected in UPLC-ESI-MS to evaluate the reaction yield. When the reaction product was detected, a two-fold volume of water was added to the reaction and the crude was freeze-dried. The dried reaction crude was then dissolved in DMSO and was purified by reverse-phase HPLC to obtain the reaction product yielding 3.2 mg (37%) of **182_24_828**. 1H NMR (600 MHz, DMSO-d6) δ 10.41 (s, 1H), 9.00 (s, 1H), 8.68 (t, J = 1.9 Hz, 1H), 8.45 (t, J = 4.2 Hz, 1H), 8.33 (t, J = 1.9 Hz, 1H), 8.10 (dd, J = 8.6, 1.3 Hz, 1H), 7.98 – 7.93 (m, 1H), 7.82 – 7.77 (m, 1H), 7.67 – 7.61 (m, 2H), 7.55 (ddd, J = 8.7, 6.7, 1.2 Hz, 1H), 7.44 (td, J = 8.7, 1.4 Hz, 1H), 4.53 (s, 1H), 3.35 (q, J = 4.2 Hz, 2H), 3.31 – 3.24 (m, 4H), 2.77 (t, J = 4.1 Hz, 2H).

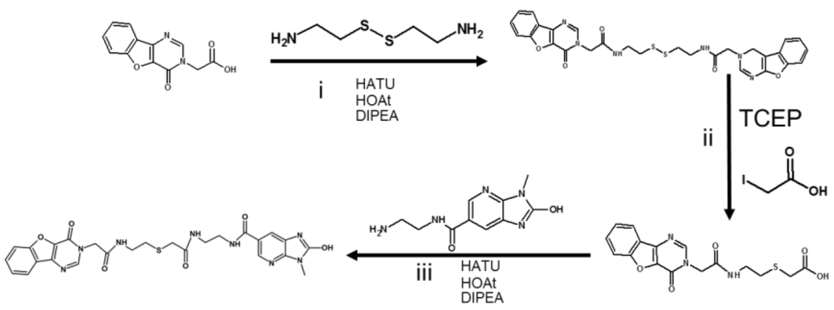

Synthesis of 182_24_826

i) 50 mg of **182** was dissolved in 3 ml DMSO and was activated by 1 eq (136 mg) HATU and 1 eq (49 mg) HOAt at RT for 20 min under argon atmosphere. 0.5 eq (40 mg) of cystamine dihydrochloride was dissolved in 2 ml of DMSO and pH was tuned to 8 by DIPEA and then added to the activated **828** drop by drop. The reaction was stirred for 3 h at RT. Small amount of reaction crude (2 µmol) was injected in UPLC-ESI-MS to evaluate the reaction yield. When the reaction product was detected, two-fold volume of water was added to the reaction and the crude was freeze-dried. The dried reaction crude was then dissolved in DMSO and was purified by reverse-phase HPLC to obtain the reaction product. HPLC fractions were injected in UPLC-ESI-MS to identify the best fraction, and the desired fractions were freeze-dried. ii) 10 mg of the reaction product was dissolved in 0.3 ml DMSO and was mixed with 2 eq of TCEP buffer (100 mM aqueous buffer, pH 7.5) and 3 eq (28.3 mg) of iodoacetic acid dissolved in 0.5 ml DMSO. The reaction was stirred at 37 ºC for 1h. The crude was freeze-dried and

purified by HPLC. Fractions were validated by UPLC-ESI-MS and fractions containing the products were freeze-dried. iii) The resulting carboxylic acid was activated by 1 eq HATU and 1 eq HOAt at RT for 20 min under argon atmosphere. To the solution, 1 eq (4 mg) **826_1** was added. The reaction was stirred for 3 h at RT. A small amount of reaction crude (2 μmol) was injected in UPLC-ESI-MS to evaluate the reaction yield. When the reaction product was detected, a two-fold volume of water was added to the reaction and the crude was freeze-dried. The dried reaction crude was then dissolved in DMSO and was purified by reverse-phase HPLC to obtain the reaction product yielding 2.3 mg (24%) of **182_24_826**. 1H NMR (600 MHz, DMSO-d6) δ 10.33 (s, 1H), 9.00 (s, 1H), 8.80 (d, J = 1.5 Hz, 1H), 8.54 (t, J = 5.2 Hz, 1H), 8.24 (d, J = 1.5 Hz, 1H), 8.10 (dd, J = 8.5, 1.3 Hz, 1H), 8.00 (t, J = 4.0 Hz, 1H), 7.83 (t, J = 4.8 Hz, 1H), 7.64 (dd, J = 6.6, 1.3 Hz, 1H), 7.54 (ddd, J = 8.7, 6.7, 1.2 Hz, 1H), 7.44 (td, J = 8.7, 1.4 Hz, 1H), 4.53 (s, 1H), 3.73 (s, 2H), 3.40 (td, J = 4.8, 3.8 Hz, 2H), 3.35 (q, J = 4.5 Hz, 2H), 3.25 (q, J = 4.1 Hz, 2H), 2.75 (t, J = 4.3 Hz, 2H).

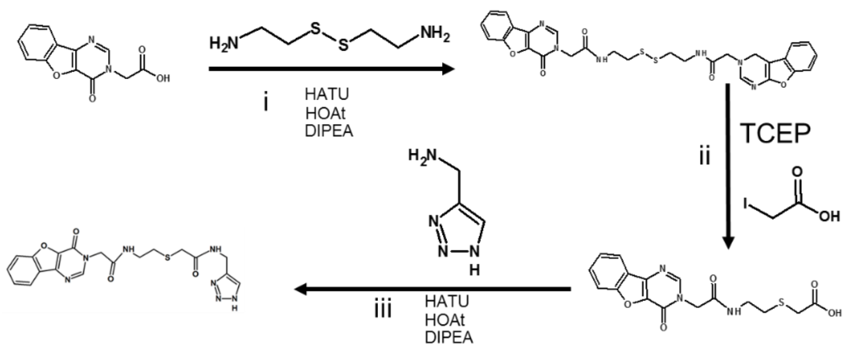

Synthesis of 182_24_787

i) 50 mg of **182** was dissolved in 3 ml DMSO and was activated by 1 eq (136 mg) HATU and 1 eq (49 mg) HOAt at RT for 20 min under argon atmosphere. 0.5 eq (40 mg) of cystamine dihydrochloride was dissolved in 2 ml of DMSO and pH was tuned to 8 by DIPEA and then added to the activated **828** drop by drop. The reaction was stirred for 3 h at RT. Small amount of reaction crude (2 μmol) was injected in UPLC-ESI-MS to evaluate the reaction yield. When the reaction product was detected, two-fold volume of water was added to the reaction and the crude was freeze-dried. The dried reaction crude was then dissolved in DMSO and was purified by reverse-phase HPLC to obtain the reaction product. HPLC fractions were injected in UPLC-ESI-MS to identify the best fraction, and the desired fractions were freeze-dried. ii) 10 mg of the reaction product was dissolved in 0.3 ml DMSO and was mixed with 2 eq of TCEP buffer (100 mM aqueous buffer, pH 7.5) and 3 eq (28.3 mg) of iodoacetic acid dissolved in 0.5 ml DMSO. The reaction was stirred at 37 ºC for 1h. The crude was freeze-dried and

purified by HPLC. Fractions were validated by UPLC-ESI-MS and fractions containing the products were freeze-dried. iii) The resulting carboxylic acid was activated by 1 eq HATU and 1 eq HOAt at RT for 20 min under argon atmosphere. To the solution, 1 eq (2.4 mg) **787** in 0.5 ml DMF, whose pH was tuned to 8 by DIPEA, was added. The reaction was stirred for 3 h at RT. Small amount of reaction crude (2 µmol) was injected in UPLC-ESI-MS to evaluate the reaction yield. When the reaction product was detected, two-fold volume of water was added to the reaction and the crude was freeze-dried. The dried reaction crude was then dissolved in DMSO and was purified by reverse-phase HPLC to obtain 1.4 mg (13%) of **182_24_787**. 1H NMR (600 MHz, DMSO-d6) δ 12.58 (d, J = 1.5 Hz, 1H), 9.00 (s, 1H), 8.59 (t, J = 5.9 Hz, 1H), 8.10 (dd, J = 8.5, 1.3 Hz, 1H), 8.07 (d, J = 1.2 Hz, 1H), 8.01 (t, J = 4.0 Hz, 1H), 7.66 – 7.62 (m, 1H), 7.55 (ddd, J = 8.7, 6.7, 1.2 Hz, 1H), 7.44 (td, J = 8.7, 1.4 Hz, 1H), 4.53 (s, 1H), 4.36 (d, J = 5.6 Hz, 2H), 3.25 (q, J = 4.2 Hz, 2H), 2.77 (t, J = 4.3 Hz, 2H).

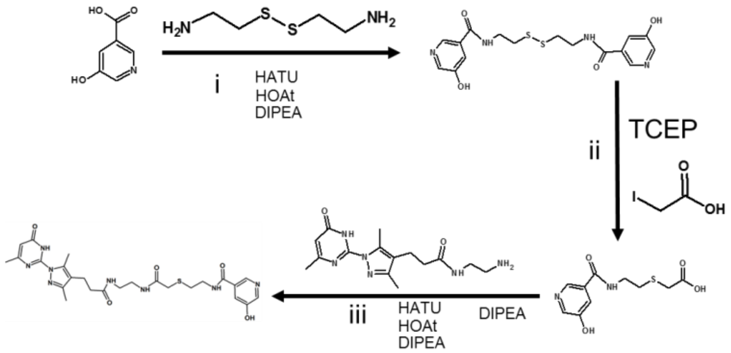

Synthesis of 693_24_828

i) 50 mg of **828** was dissolved in 3 ml DMSO and was activated by 1 eq (137 mg) HATU and 1 eq (49 mg) HOAt at RT for 20 min under argon atmosphere. 0.5 eq (40.5mg) cystamine dihydrochloride was dissolved in 2 ml of DMSO and pH was tuned to 8 by DIPEA and then added to the activated **828** drop by drop. The reaction was stirred for 3 h at RT. A small amount of reaction crude (2 µmol) was injected in UPLC-ESI-MS to evaluate the reaction yield. When the reaction product was detected, a two-fold volume of water was added to the reaction, and the crude was freeze-dried. The dried reaction crude was then dissolved in DMSO and was purified by reverse-phase HPLC to obtain the reaction product. HPLC fractions were injected in UPLC-ESI-MS to identify the best fraction, and the desired fractions were freeze-dried. ii) 10 mg of the reaction product was dissolved in 0.3 ml DMSO and was mixed with 2 eq of TCEP buffer (100 mM aqueous buffer, pH 7.5) and 3 eq (100 mg) of iodoacetic acid dissolved in 0.5 ml DMSO. The reaction was stirred at 37 ºC for 1h. The crude was freeze-dried and purified by HPLC. Fractions were validated by UPLC-ESI-MS and fractions containing the products were free-dried. iii) The resulting carboxylic acid was activated by 1 eq HATU and 1 eq HOAt at RT for 20 min under argon atmosphere. 5 mg of **693_1** was added to the reaction.

The reaction was stirred for 3 h at RT. A small amount of reaction crude (2 µmol) was injected in UPLC-ESI-MS to evaluate the reaction yield. When the reaction product was detected, a two-fold volume of water was added to the reaction and the crude was freeze-dried. The dried reaction crude was then dissolved in DMSO and was purified by reverse-phase HPLC to obtain the reaction product yielding 1.5 mg (17%) of **693_24_828**. 1H NMR (600 MHz, DMSO-d6) δ 10.54 (s, 1H), 10.41 (s, 1H), 8.67 (t, J = 1.9 Hz, 1H), 8.46 (t, J = 4.2 Hz, 1H), 8.33 (t, J = 1.9 Hz, 1H), 7.82 – 7.77 (m, 1H), 7.71 – 7.66 (m, 1H), 7.63 (t, J = 2.0 Hz, 1H), 5.82 (q, J = 1.2 Hz, 1H), 3.35 (q, J = 4.2 Hz, 2H), 3.29 – 3.23 (m, 4H), 2.88 (dt, J = 17.7, 8.0 Hz, 1H), 2.80 – 2.71 (m, 2H), 2.52 – 2.45 (m, 5H), 2.44 (s, 2H), 2.17 (s, 2H).

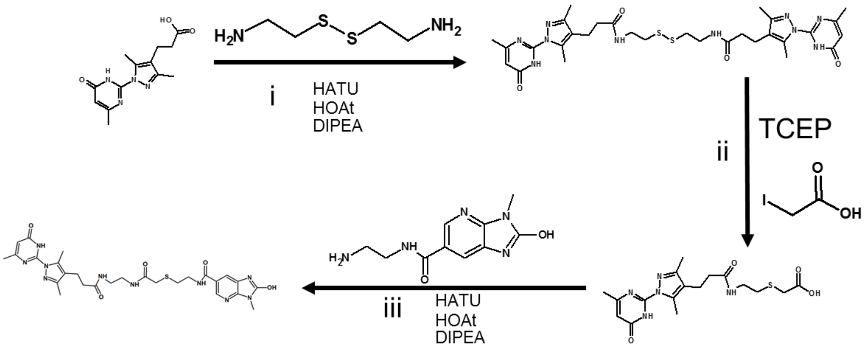

Synthesis of 693_24_826

i) 50 mg of **693** was dissolved in 1 ml DMF and was activated by 1 eq (69 mg) HATU and 1 eq (24 mg) HOAt at RT for 20 min under argon atmosphere. 0.5 eq (23 mg) cystamine dihydrochloride was dissolved in 2 ml of DMSO and pH was tuned to 8 by DIPEA and then added to the activated **828** drop by drop. The reaction was stirred for 3 h at RT. A small amount of reaction crude (2 µmol) was injected in UPLC-ESI-MS to evaluate the reaction yield. When the reaction product was detected, a two-fold volume of water was added to the reaction and the crude was freeze-dried. The dried reaction crude was then dissolved in DMSO and was purified by reverse-phase HPLC to obtain the reaction product. HPLC fractions were injected in UPLC-ESI-MS to identify the best fraction, and the desired fractions were freeze-dried. ii) 10 mg of the reaction product was dissolved in 0.3 ml DMSO and was mixed with 2 eq of TCEP buffer (100 mM aqueous buffer, pH 7.5) and 3 eq (50.5 mg) of iodoacetic acid dissolved in 0.5 ml DMSO. The reaction was stirred at 37 ºC for 1h. The crude was freeze-dried and purified by HPLC. Fractions were validated by UPLC-ESI-MS and fractions containing the products were free-dried. iii) The resulting carboxylic acid was activated by 1 eq HATU and 1 eq HOAt at RT for 20 min under argon atmosphere. To the solution, 4 mg **826_1** was added. The reaction was stirred for 3 h at RT. Small amount of reaction crude (2 µmol) was injected in UPLC-ESI-MS to evaluate the reaction yield. When the reaction product was detected, two-fold volume of water was added to the reaction and the crude was freeze-dried. The dried

reaction crude was then dissolved in DMSO and was purified by reverse-phase HPLC to obtain the reaction product yielding 2.7 mg (26%) of **693_24_826**. 1H NMR (600 MHz, DMSO-d6) δ 10.54 (s, 1H), 10.33 (s, 1H), 8.80 (d, J = 1.5 Hz, 1H), 8.50 (t, J = 4.4 Hz, 1H), 8.24 (d, J = 1.5 Hz, 1H), 7.82 – 7.77 (m, 1H), 7.71 – 7.66 (m, 1H), 5.82 (q, J = 1.3 Hz, 1H), 3.74 (s, 2H), 3.32 (q, J = 4.1 Hz, 2H), 3.29 – 3.23 (m, 4H), 2.88 (dt, J = 17.7, 8.0 Hz, 1H), 2.80 – 2.71 (m, 2H), 2.51 – 2.45 (m, 5H), 2.44 (s, 2H), 2.16 (s, 2H).

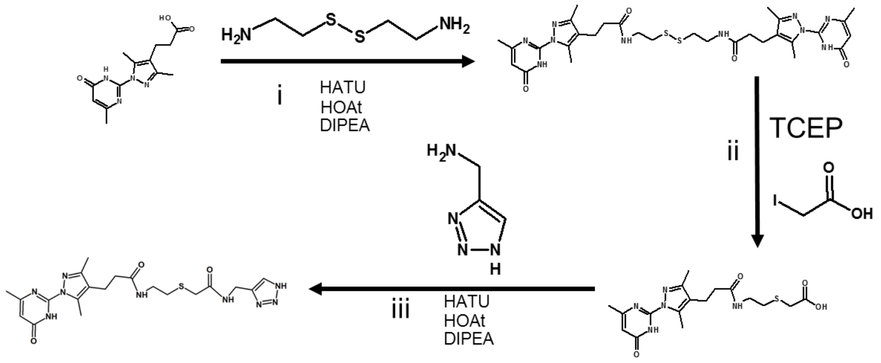

Synthesis of 693_24_787

i) 50 mg of **693** was dissolved in 1 ml DMF and was activated by 1 eq (69 mg) HATU and 1 eq (24 mg) HOAt at RT for 20 min under argon atmosphere. 0.5 eq (23 mg) cystamine dihydrochloride was dissolved in 2 ml of DMSO and pH was tuned to 8 by DIPEA and then added to the activated **828** drop by drop. The reaction was stirred for 3 h at RT. A small amount of reaction crude (2 µmol) was injected in UPLC-ESI-MS to evaluate the reaction yield. When the reaction product was detected, a two-fold volume of water was added to the reaction and the crude was freeze-dried. The dried reaction crude was then dissolved in DMSO and was purified by reverse-phase HPLC to obtain the reaction product. HPLC fractions were injected in UPLC-ESI-MS to identify the best fraction, and the desired fractions were freeze-dried. ii) 10 mg of the reaction product was dissolved in 0.3 ml DMSO and was mixed with 2 eq of TCEP buffer (100 mM aqueous buffer, pH 7.5) and 3 eq (50.5 mg) of iodoacetic acid dissolved in 0.5 ml DMSO. The reaction was stirred at 37 ºC for 1h. The crude was freeze-dried and purified by HPLC. Fractions were validated by UPLC-ESI-MS and fractions containing the products were free-dried. iii) The resulting carboxylic acid was activated by 1 eq HATU and 1 eq HOAt at RT for 20 min under argon atmosphere. To the solution was added 1.8 mg **787** in 0.5 ml DMF, whose pH was tuned to 8 by DIPEA. The reaction was stirred for 3 h at RT. A small amount of reaction crude (2 µmol) was injected in UPLC-ESI-MS to evaluate the reaction yield. When the reaction product was detected, a two-fold volume of water was added to the reaction and the crude was freeze-dried. The dried reaction crude was then dissolved in DMSO and was purified by reverse-phase HPLC to obtain the reaction product yielding 2 mg (23%) of **693_24_787**. 1H NMR (600 MHz, DMSO-d6) δ 12.58 (d, J = 1.5 Hz, 1H), 10.54 (s,

1H), 8.59 (t, J = 5.9 Hz, 1H), 8.07 (d, J = 1.2 Hz, 1H), 7.70 (t, J = 4.2 Hz, 1H), 5.82 (q, J = 1.2 Hz, 1H), 4.36 (d, J = 5.6 Hz, 2H), 3.24 (q, J = 4.3 Hz, 2H), 2.88 (dt, J = 17.8, 8.0 Hz, 1H), 2.77 – 2.71 (m, 3H), 2.51 – 2.45 (m, 5H), 2.44 (s, 2H), 2.17 (s, 2H).

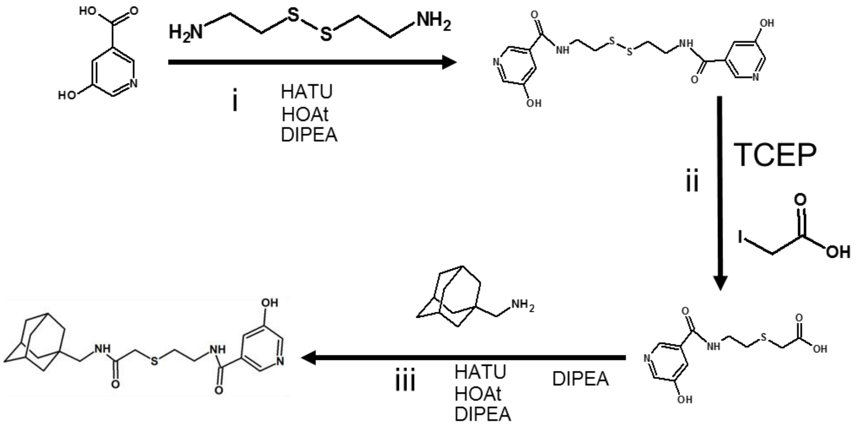

Synthesis of 66_24_828

i) 50 mg of **828** was dissolved in 3 ml DMSO and was activated by 1 eq (137 mg) HATU and 1 eq (49 mg) HOAt at RT for 20 min under argon atmosphere. 0.5 eq (40.5mg) cystamine dihydrochloride was dissolved in 2 ml of DMSO and pH was tuned to 8 by DIPEA and then added to the activated 828 drop by drop. The reaction was stirred for 3 h at RT. A small amount of reaction crude (2 µmol) was injected in UPLC-ESI-MS to evaluate the reaction yield. When the reaction product was detected, a two-fold volume of water was added to the reaction, and the crude was freeze-dried. The dried reaction crude was then dissolved in DMSO and was purified by reverse-phase HPLC to obtain the reaction product. HPLC fractions were injected in UPLC-ESI-MS to identify the best fraction, and the desired fractions were freeze-dried. ii) 10 mg of the reaction product was dissolved in 0.3 ml DMSO and was mixed with 2 eq of TCEP buffer (100 mM aqueous buffer, pH 7.5) and 3 eq (100 mg) of iodoacetic acid dissolved in 0.5 ml DMSO. The reaction was stirred at 37 ºC for 1h. The crude was freeze-dried and purified by HPLC. Fractions were validated by UPLC-ESI-MS and fractions containing the products were free-dried. iii) The resulting carboxylic acid was activated by 1 eq HATU and 1 eq HOAt at RT for 20 min under argon atmosphere. 3 mg of 1-adamantane methyl amine was added to the reaction. The reaction was stirred for 3 h at RT. A small amount of reaction crude (2 µmol) was injected in UPLC-ESI-MS to evaluate the reaction yield. When the reaction product was detected, a two-fold volume of water was added to the reaction and the crude was freeze-dried. The dried reaction crude was then dissolved in DMSO and was purified by reverse-phase HPLC to obtain the reaction product yielding 2.2 mg (30%) of **66_24_828**. 1H NMR (600 MHz, DMSO-d6) δ 10.41 (s, 1H), 8.68 (t, J = 1.9 Hz, 1H), 8.46 (t, J = 4.2 Hz, 1H), 8.33 (t, J = 1.9 Hz, 1H), 7.62 (t, J = 2.0 Hz, 1H), 7.49 (t, J = 5.8 Hz, 1H), 3.35 (q, J = 4.2 Hz,

2H), 3.07 (d, J = 5.9 Hz, 2H), 2.76 (t, J = 4.1 Hz, 2H), 2.03 – 1.94 (m, 3H), 1.64 (t, J = 5.8 Hz, 5H), 1.49 (d, J = 5.0 Hz, 6H).

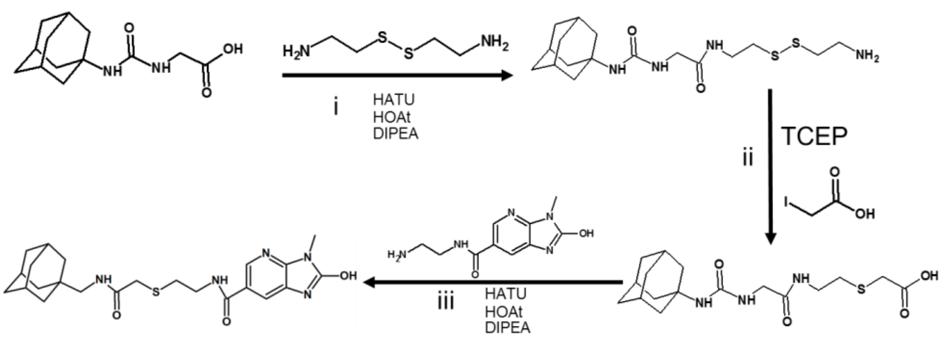

Synthesis of 66_24_826

i) 50 mg of **66** was dissolved in 1 ml DMF and was activated by 1 eq (75 mg) HATU and 1 eq (27 mg) HOAt at RT for 20 min under argon atmosphere. 5 eq (250 mg) cystamine dihydrochloride was dissolved in 5 ml DMSO and pH was tuned to 8 by DIPEA. Activated **66** was added to cystamine solution drop by drop. The reaction was stirred for 3 h at RT. A small amount of reaction crude (2 µmol) was injected in UPLC-ESI-MS to evaluate the reaction yield. When the reaction product was detected, a two-fold volume of water was added to the reaction and the crude was freeze-dried. The dried reaction crude was then dissolved in DMSO and was purified by reverse-phase HPLC to obtain the reaction product. HPLC fractions were injected in UPLC-ESI-MS to identify the best fraction, and the desired fractions were freeze-dried. ii) 10 mg of the reaction product was dissolved in 0.3 ml DMSO and was mixed with 2 eq of TCEP buffer (100 mM aqueous buffer, pH 7.5) and 3 eq of iodoacetic acid dissolved in 0.5 ml DMSO. The reaction was stirred at 37 ºC for 1h. The crude was freeze-dried and purified by HPLC. Fractions were validated by UPLC-ESI-MS and fractions containing the products were free-dried. iii) The resulting carboxylic acid was activated by 1 eq HATU and 1 eq HOAt at RT for 20 min under argon atmosphere. To the solution, 1 eq (4 mg) **826_1** was added. The reaction was stirred for 3 h at RT. A small amount of reaction crude (2 µmol) was injected in UPLC-ESI-MS to evaluate the reaction yield. When the reaction product was detected, a two-fold volume of water was added to the reaction and the crude was freeze-dried. The dried reaction crude was then dissolved in DMSO and was purified by reverse-phase HPLC to obtain the reaction product yielding 2.7 mg (29%) of **66_24_826**. 1H NMR (600 MHz, DMSO-d6) δ 10.33 (s, 1H), 8.80 (d, J = 1.5 Hz, 1H), 8.50 (t, J = 4.4 Hz, 1H), 8.24 (d, J = 1.5 Hz, 1H), 7.49 (t, J = 5.8 Hz, 1H), 3.73 (s, 2H), 3.32 (q, J = 4.1 Hz, 2H), 3.06 (d, J = 5.9 Hz, 2H), 2.77 (t, J = 4.1 Hz, 2H), 2.03 – 1.95 (m, 3H), 1.64 (t, J = 5.8 Hz, 5H), 1.48 (d, J = 5.0 Hz, 6H).

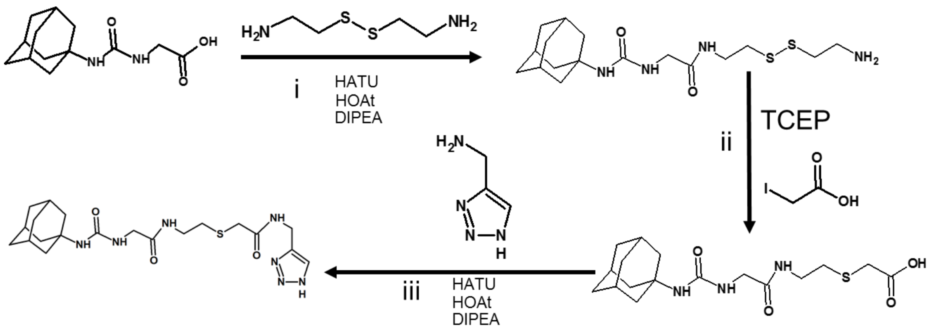

Synthesis of 66_24_787

i) 50 mg of **66** was dissolved in 1 ml DMF and was activated by 1 eq (75 mg) HATU and 1 eq (27 mg) HOAt at RT for 20 min under argon atmosphere. 5 eq (250 mg) cystamine dihydrochloride was dissolved in 5 ml DMSO and pH was tuned to 8 by DIPEA. Activated **66** was added to cystamine solution drop by drop. The reaction was stirred for 3 h at RT. A small amount of reaction crude (2 µmol) was injected in UPLC-ESI-MS to evaluate the reaction yield. When the reaction product was detected, a two-fold volume of water was added to the reaction and the crude was freeze-dried. The dried reaction crude was then dissolved in DMSO and was purified by reverse-phase HPLC to obtain the reaction product. HPLC fractions were injected in UPLC-ESI-MS to identify the best fraction, and the desired fractions were freeze-dried. ii) 10 mg of the reaction product was dissolved in 0.3 ml DMSO and was mixed with 2 eq of TCEP buffer (100 mM aqueous buffer, pH 7.5) and 3 eq of iodoacetic acid dissolved in 0.5 ml DMSO. The reaction was stirred at 37 ^{o}C for 1h. The crude was freeze-dried and purified by HPLC. Fractions were validated by UPLC-ESI-MS and fractions containing the products were free-dried. iii) The resulting carboxylic acid was activated by 1 eq HATU and 1 eq HOAt at RT for 20 min under argon atmosphere. To the solution, 2.3 mg **787** dissolved 1 ml DMSO was added. The reaction was stirred for 3 h at RT. A small amount of reaction crude (2 µmol) was injected in UPLC-ESI-MS to evaluate the reaction yield. When the reaction product was detected, a two-fold volume of water was added to the reaction, and the crude was freeze-dried. The dried reaction crude was then dissolved in DMSO and was purified by reverse-phase HPLC to obtain the reaction product yielding 2.1 mg (20%) of **66_24_787**. 1H NMR (600 MHz, DMSO-d6) δ 12.58 (d, J = 1.5 Hz, 2H), 8.59 (t, J = 5.9 Hz, 2H), 8.07 (d, J = 1.2 Hz, 2H), 7.86 (s, 1H), 7.86 (d, J = 7.8 Hz, 1H), 6.10 (t, J = 5.3 Hz, 2H), 5.47 (s, 1H), 4.36 (d, J = 5.6 Hz, 4H), 3.73 (d, J = 5.3 Hz, 4H), 3.25 (q, J = 4.1 Hz, 4H), 2.75 (s, 2H), 2.75 (d, J = 8.4 Hz, 2H), 2.05 (hept, J = 5.3 Hz, 6H), 1.94 (d, J = 5.2 Hz, 12H), 1.65 (t, J = 5.4 Hz, 11H).

Supplementary figures

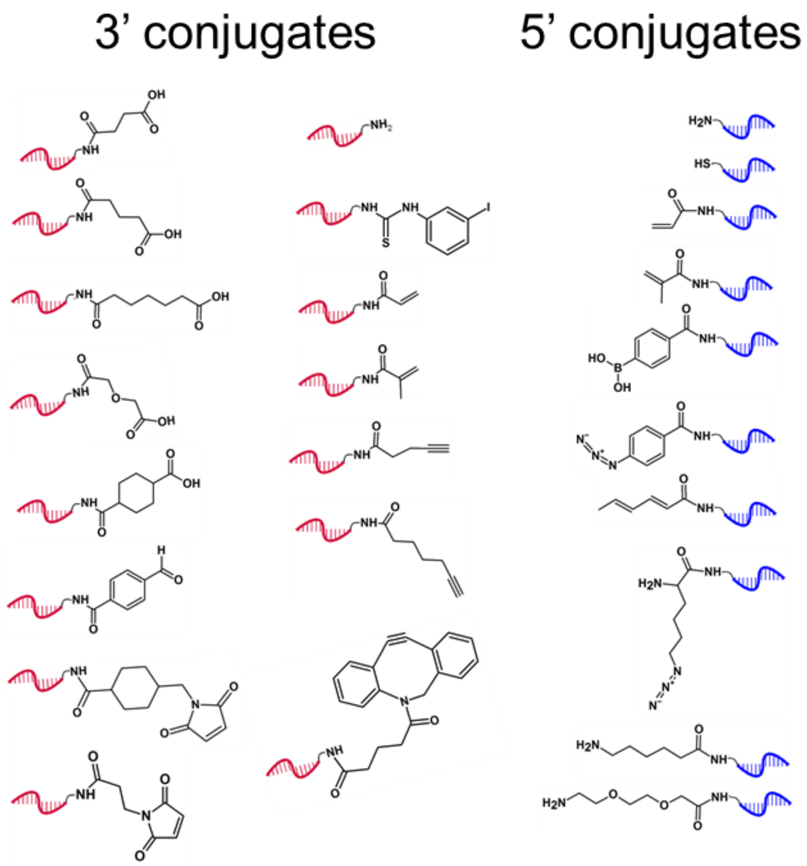

Supplementary Figure 1: Single side DNA-compound conjugates. Bi-functional building blocks were conjugated to amine-functionalized oligonucleotides to generate the conjugates displaying a functional group at the terminal.

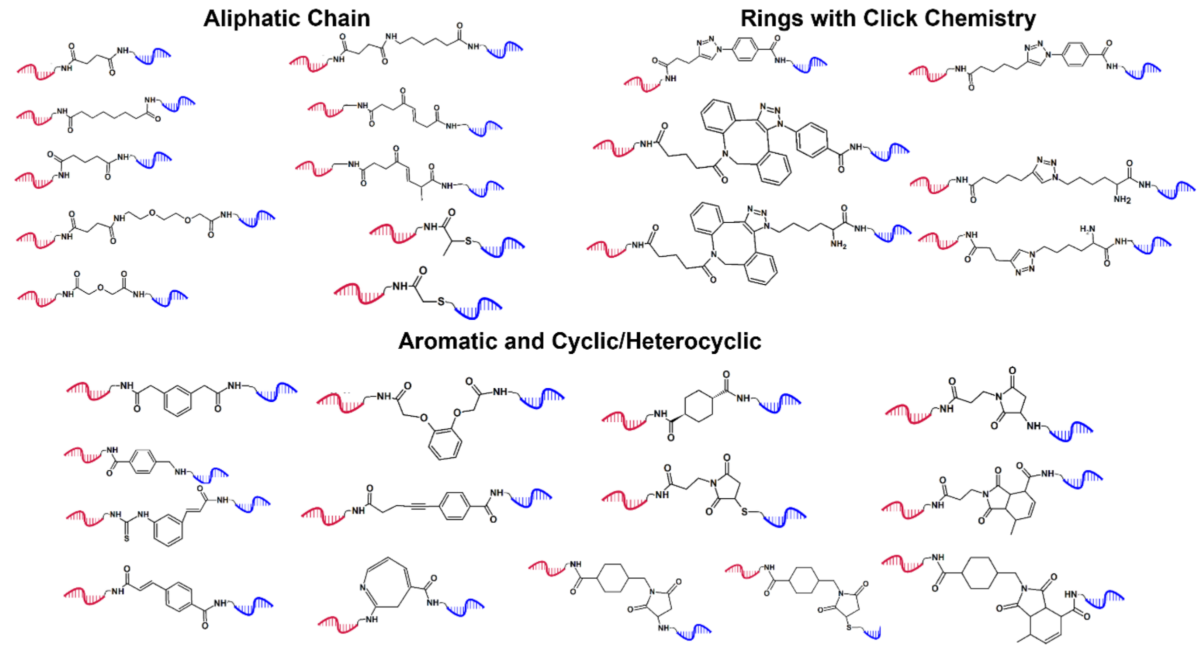

Supplementary Figure 2. 30-member DNA-encoded sub-library B. The compounds covered three structural categories: Aliphatic chain, rings with click chemistry, and aromatic and cyclic/heterocyclic rings.

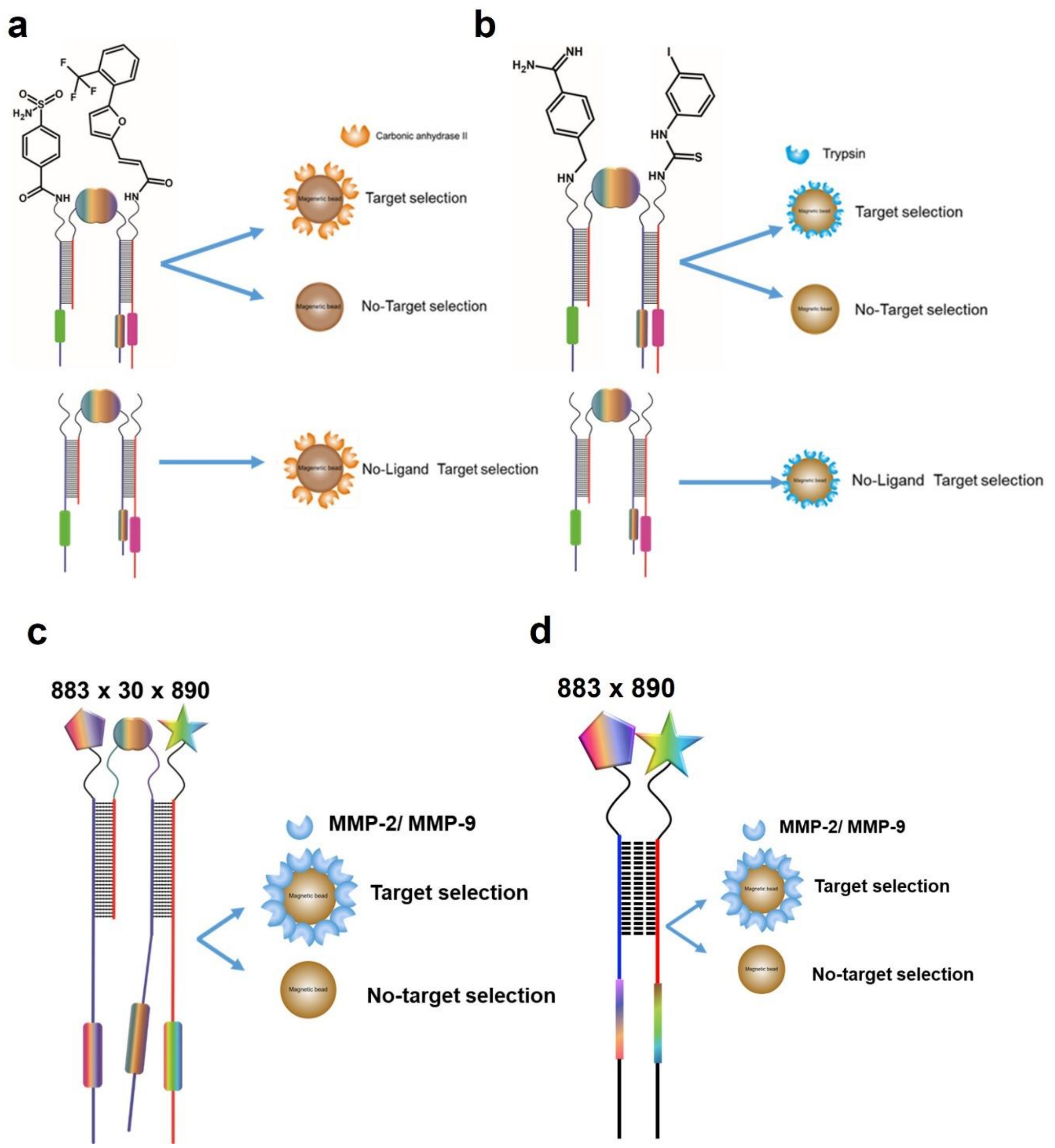

Supplementary Figure 3. Selection schemes of linker optimization for CAII, trypsin and de novo ligand selection against MMP-2 and MMP-9. (a) Scheme of affinity maturation of ligand pair binding to bovine CAII. CBS and compound A were conjugated to amine-functionalized oligonucleotides and further encoded with different barcode DNAs to serve as the single-member SL-A and SL-C, respectively. After assembling with sublibrary B, the 30-member library was subjected to the selection against CAII. Selection against blank solid support was served as the no-target control. Sub-libraries A and C without the ligand pair were assembled with sublibrary B and used to perform no-ligand target selection. (b) Scheme of affinity maturation of ligand pair binding to bovine trypsin. 4-aminomethyl benzamidine and compound B were conjugated to amine-functionalized oligonucleotides and further encoded with different barcode DNAs to serve as the single-member SL-A and SL-C, respectively. After assembling with sublibrary B, the 30-member library was subjected to the selection against bovine trypsin. Selection against blank solid support was served as the no-target control. Sub-libraries A and

C without the ligand pair were assembled with sublibrary B and used to perform no-ligand target selection. (c) The real trio-pharmacophore library was selected against MMP-2, MMP-9 and blank solid support. (d) The dual pharmacophore DEL with the same fragment composition as in T-DEL was selected against MMP-2, MMP-9 and blank solid support.

Supplementary Discussion 1

Analysis on docking studies with CAII and its ligands

To compare the docking poses of all compounds, we mapped the distances between the terminal -CF_3 group of the small molecules and the surrounding residues. Additionally, the fragment pair sulfanilamide and compound A were docked against the target simultaneously to identify their binding sites (Supplementary Fig. 4c). As depicted in the heatmap (Supplementary Fig. 5a), C-2, C-5 and the fragment pair belonged to the same group, and C-1, C-3 and C-4 5B fell into one group. The position of -CF_3 group is indicated by the red dashed circle in Supplementary Fig. 5. The binding pose of C-0 was distinct from other compounds, and its binding pose was mimicking the ligand in the reported crystal structure (Supplementary Fig.5a). Interestingly, the binding of individual fragments was recapitulated in compounds C-2, and C-5, as can be observed in Supplementary Fig. 4c and 5c.

a

	Docking Score	Hydrophobic Contacts	H-bonds	Asn_67	Thr_198	Thr_199	Asn_62	Gln_92	Pro_200
Sulfanilamide	-8.31	38	3	0	2	0	0	0	1
Compound A	-5.44	60	2	0	0	0	2	0	0
Sulfanilamide +Compound A	-14.49	84	3	0	2	0	0	1	0
C-0	-10.58	71	2	0	2	0	0	0	0
C-1	-13.02	114	4	1	2	1	0	0	0
C-2	-12.37	110	5	1	2	1	0	1	0
C-3	-10.8	86	2	0	2	0	0	0	0
C-4	-9.565	92	5	1	3	0	0	1	0
C-5	-11.27	84	5	2	2	0	1	0	0

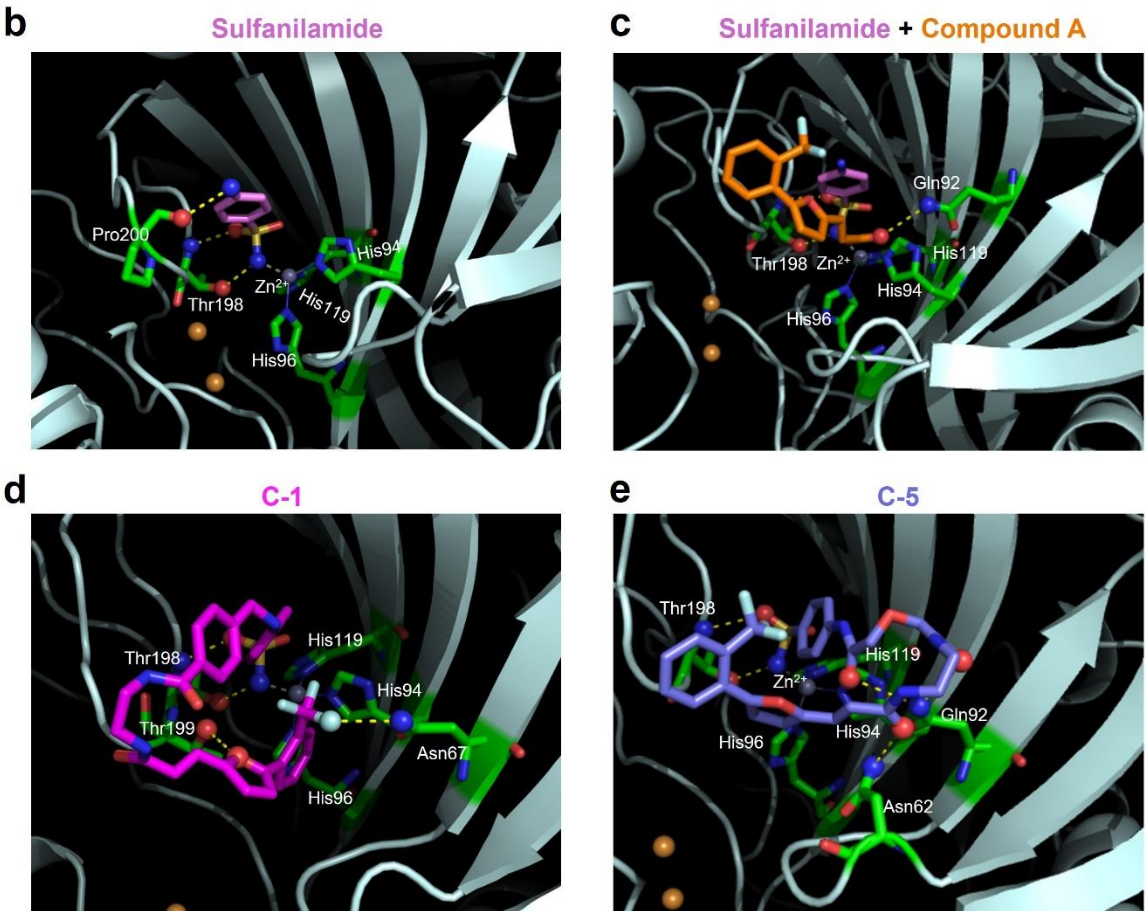

Supplementary Figure 4. (a) Summary of interactions between small molecule compounds and bovine carbonic anhydrase II (CAII) (PDB: "6SKV [https://www.rcsb.org/structure/6skv]"). (b) Docking pose of sulfanilamide in complex with CAII. (c) Simultaneous docking of the fragment pair: sulfanilamide and compound A in complex with CAII. (d) Docking pose of C-1 in complex with CAII. (e) Docking pose of C-5 in complex with CAII Proteins are in cartoon style, certain residues and the compounds are in stick representation, and the hydrogen bond-forming atoms are in ball representation. Yellow dashed lines indicate hydrogen bonds and grey dashed lines stand for the coordination with Zn^{2+}. The grey sphere represents Zn^{2+}, and the orange spheres represent Cu^{2+}.

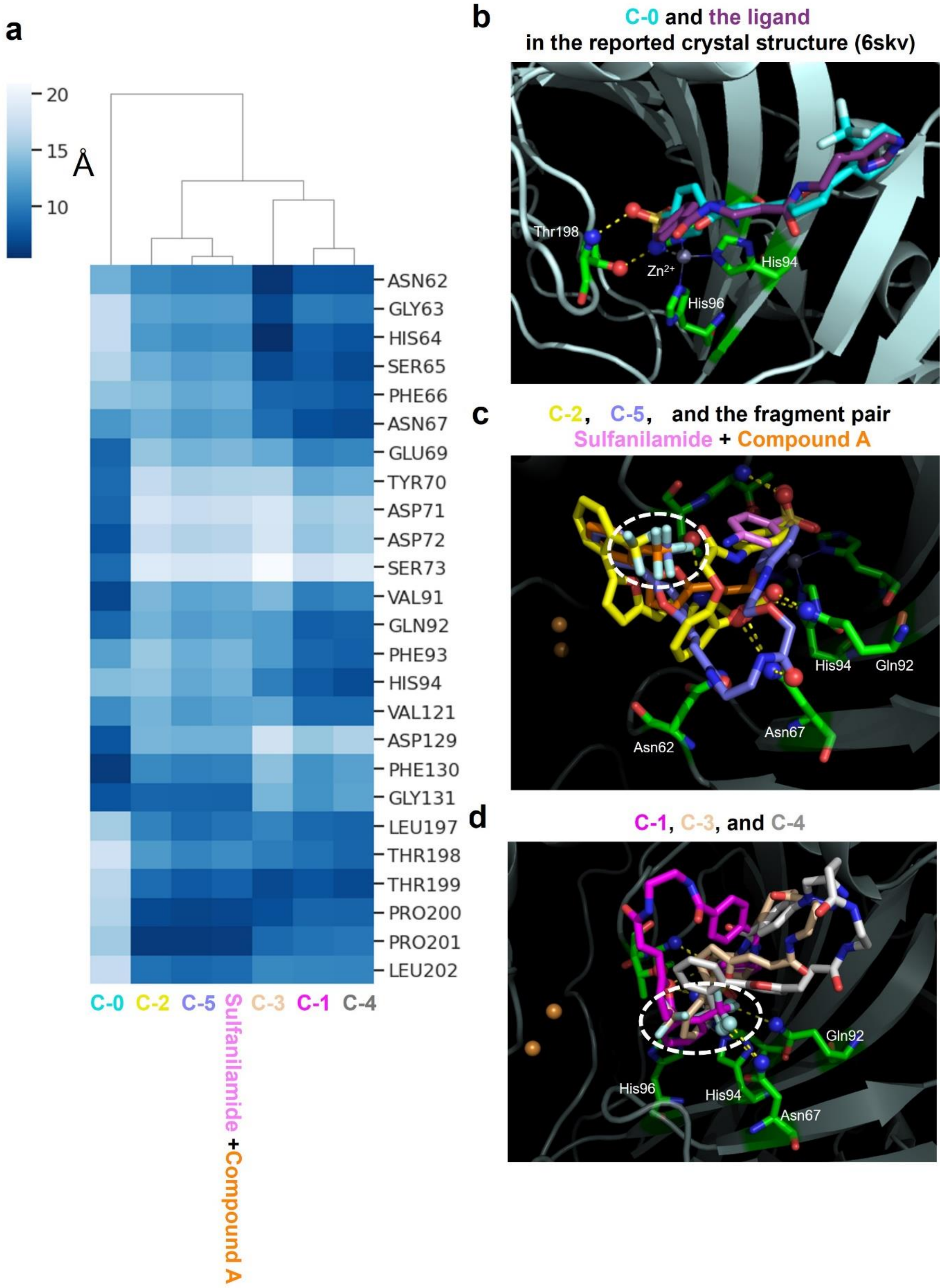

Supplementary Figure 5. (a) Heatmap of the distances between the terminal $-CF_3$ group of the compounds and the neighboring residues in the active site of CAII (PDB: "6SKV [https://www.rcsb.org/structure/6skv]"). (b) Superimposed docking poses of C-0 and the reported ligand in the crystal structure. (c) Superimposed docking poses of C-2, C-5, and the

fragment pair: sulfanilamide and compound A in complex with CAII. (d) Superimposed docking poses of C-1, C-3, and C-4 in complex with CAII. Dashed white circles in (c) and (d) indicate the position of $-CF_3$ groups. Proteins are in cartoon style, certain residues and the compounds are in stick representation, and the hydrogen bond-forming atoms are in ball representation. The grey sphere represents Zn^{2+}, and the orange spheres represent Cu^{2+}. Yellow dashed lines indicate hydrogen bonds.

Supplementary Discussion 2

Analysis on docking studies with bovine trypsin and its ligands

To compare the docking poses of all compounds, we mapped the distances between the terminal iodine atom of the small molecules and the surrounding residues (Supplementary Fig. 7a). Additionally, the fragment pair 4-aminometyl benzamidine and compound B were docked against the target simultaneously to identify their binding sites (Supplementary Fig. 6d). According to the heatmap, T-0 and T-1 fell in one group, and T-2 and T-4 formed a second group, while T-5 did not exhibit similarity with other compounds in the position of the iodine.

a

	Docking Score	Hydrophobic Contacts	H-bonds	Asp_189	Ser_190	Gly_219	Ser_195	Gln_192	Ser_214	Trp_215	Asn_97	Ser_217	Gly_216	Gln_175
4-amino methyl benzamidine	-5.929	33	5	2	1	1	1	0	0	0	0	0	0	0
Compound B	-4.256	24	0	0	0	0	0	0	0	0	0	0	0	0
4-amino methyl benzamidine + Compound B	-9.062	73	5	2	1	1	1	0	0	0	0	0	0	0
T-0	-7.323	52	5	2	1	1	0	0	1	0	0	0	0	0
T-1	-7.056	74	6	2	1	1	0	0	0	0	0	1	0	1
T-2	-7.385	80	6	2	1	1	0	0	0	1	0	0	1	0
T-3	-7.007	70	5	2	1	1	0	0	0	0	1	0	0	0
T-4	-7.259	73	5	2	1	1	0	1	0	0	0	0	0	0
T-5	-6.474	72	6	2	1	1	0	0	0	0	0	0	2	0

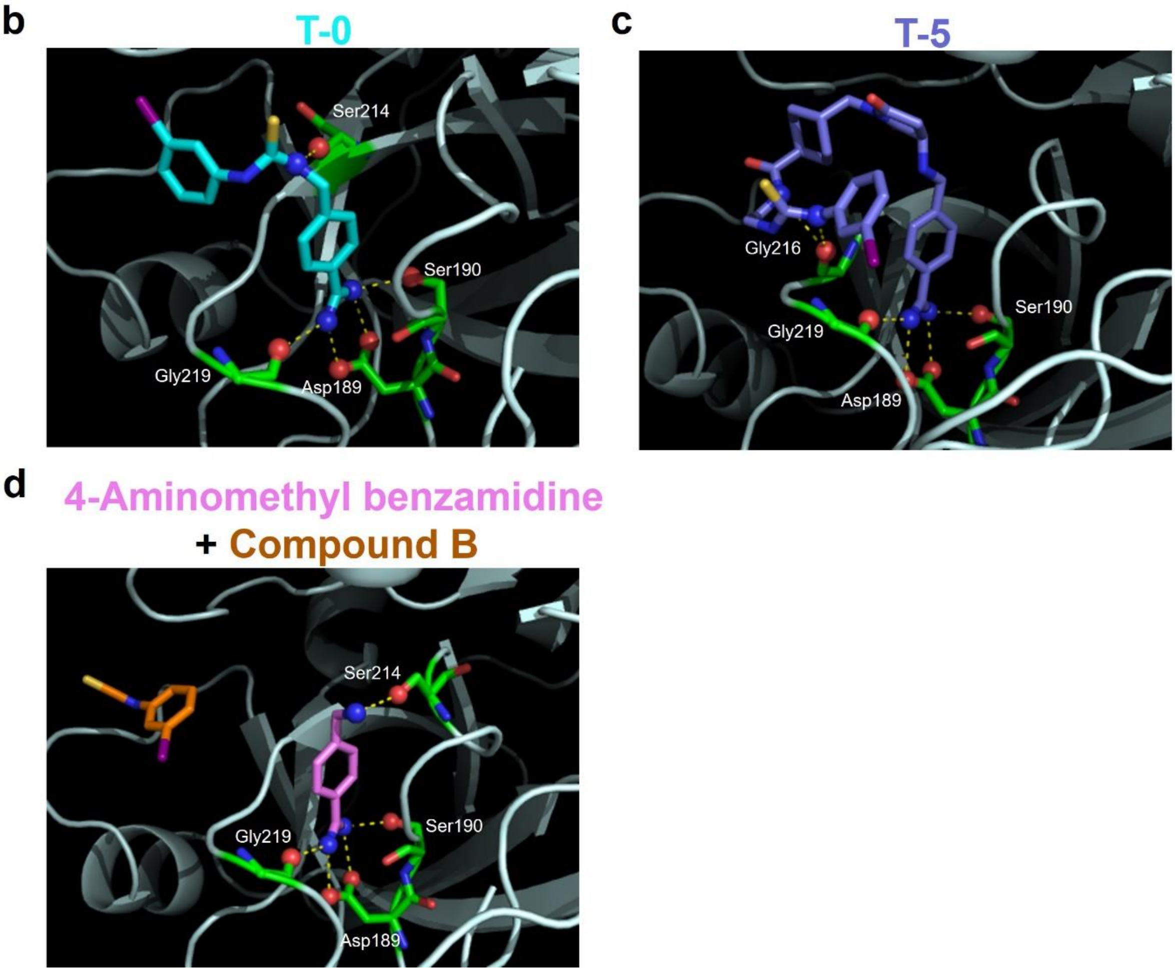

Supplementary Figure 6. (a) Summary of interactions between small molecule compounds and bovine trypsin in terms of docking score, hydrophobic contact, and hydrogen bonds (H-bonds). (b) Docking pose of T-0 in complex with bovine trypsin (PDB: "1BTY [https://www.rcsb.org/structure/1BTY]"). (c) Docking pose of T-5 in complex with bovine trypsin. (d) Simultaneous docking of the fragment pair: 4-aminomethyl benzamidine and compound B. Proteins are in cartoon style, certain residues and the compounds are in stick representation, and the hydrogen bond-forming atoms are in ball representation. Yellow dashed lines indicate hydrogen bonding.

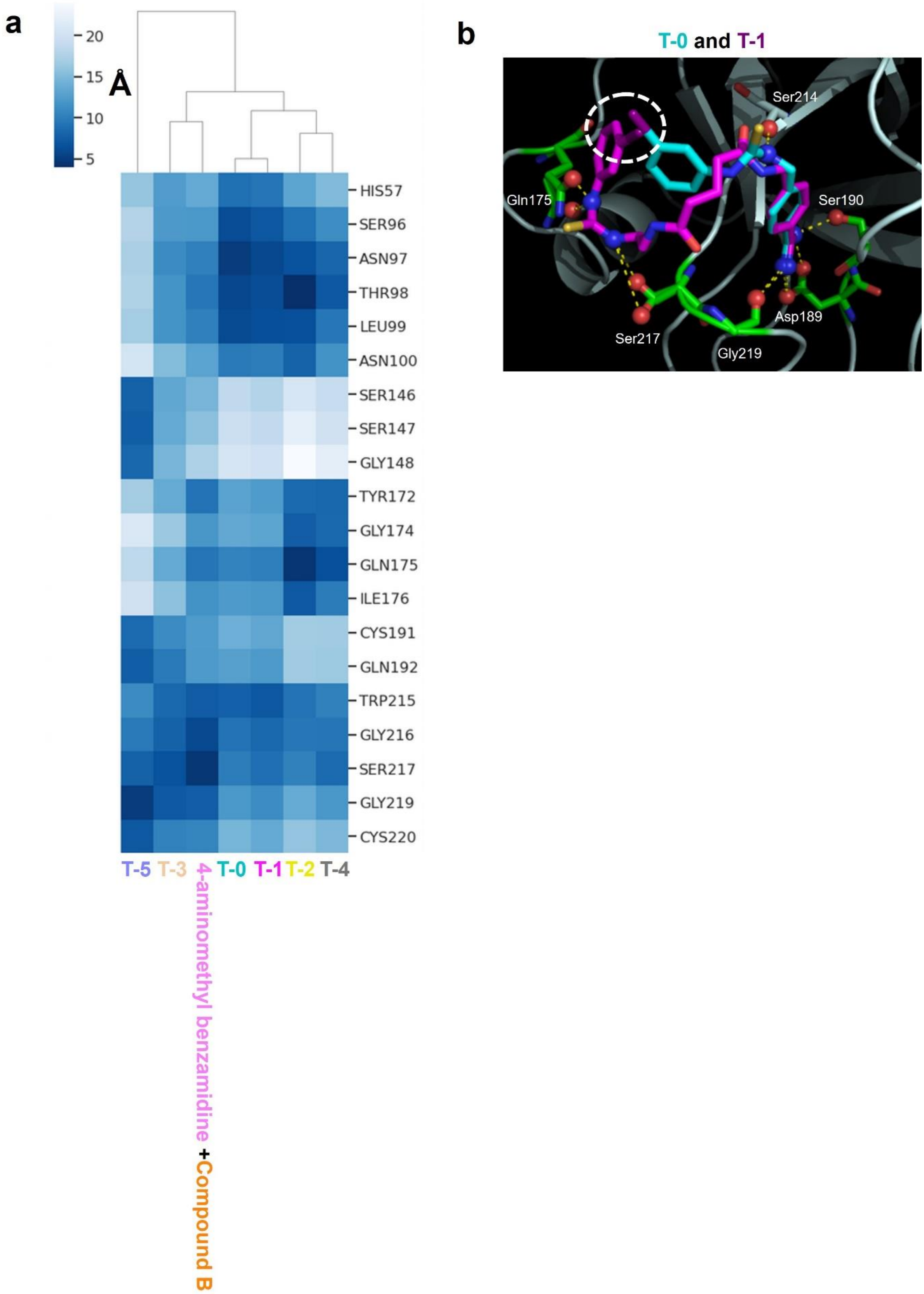

Supplementary Figure 7. (a) Heatmap of the distances between terminal iodine atom and the residues in bovine trypsin (PDB: "1BTY [https://www.rcsb.org/structure/1BTY]"). (b)

Superimposed docking poses of T-0 and T-1 in complex with bovine trypsin. The white dashed circle shows the position of the iodine in both compounds. Proteins are in cartoon style, certain residues and the compounds are in stick representation, and the hydrogen bond-forming atoms are in ball representation.

Supplementary Discussion 3

Comparison of T-DEL format to the dual-pharmacophore DEL in the recovery of the potent ligands from selections.

We explored the enrichment of the two fragments in dual and trio formats to evaluate the performance of T-DEL. As demonstrated in Supplementary Fig. 8, we measured the recovery of the ligand pair from selections against CAII and trypsin with the respective ligand pairs using qPCR. Each sub-library was added at the final concentration of 1 nM in 100 µl volume in each selection. The eluted amount of each sub-library was measured by qPCR. The assays were performed in dual- and trio-pharmacophore formats in parallel (Supplementary Fig. 8a), and each selection was done in triplicates. As observed in Supplementary Fig. 8cc and 8d, the ligand pair of CAII (CBS and compound A) displayed higher recovery than that of trypsin in target selection agreeing with the measured affinity (Fig. 2c and 3c). Both dual- and trio-pharmacophore formats delivered comparable recovery of the ligand pair of CAII (Supplementary Fig. 8c). However, the weaker ligand pair 4-aminomethyl benzamidine and compound B enriched less in trio-pharmacophore format (Supplementary Fig. 8d). A similar pattern was also observed in another target alpha-1-acid glycoprotein (Supplementary Fig.11).

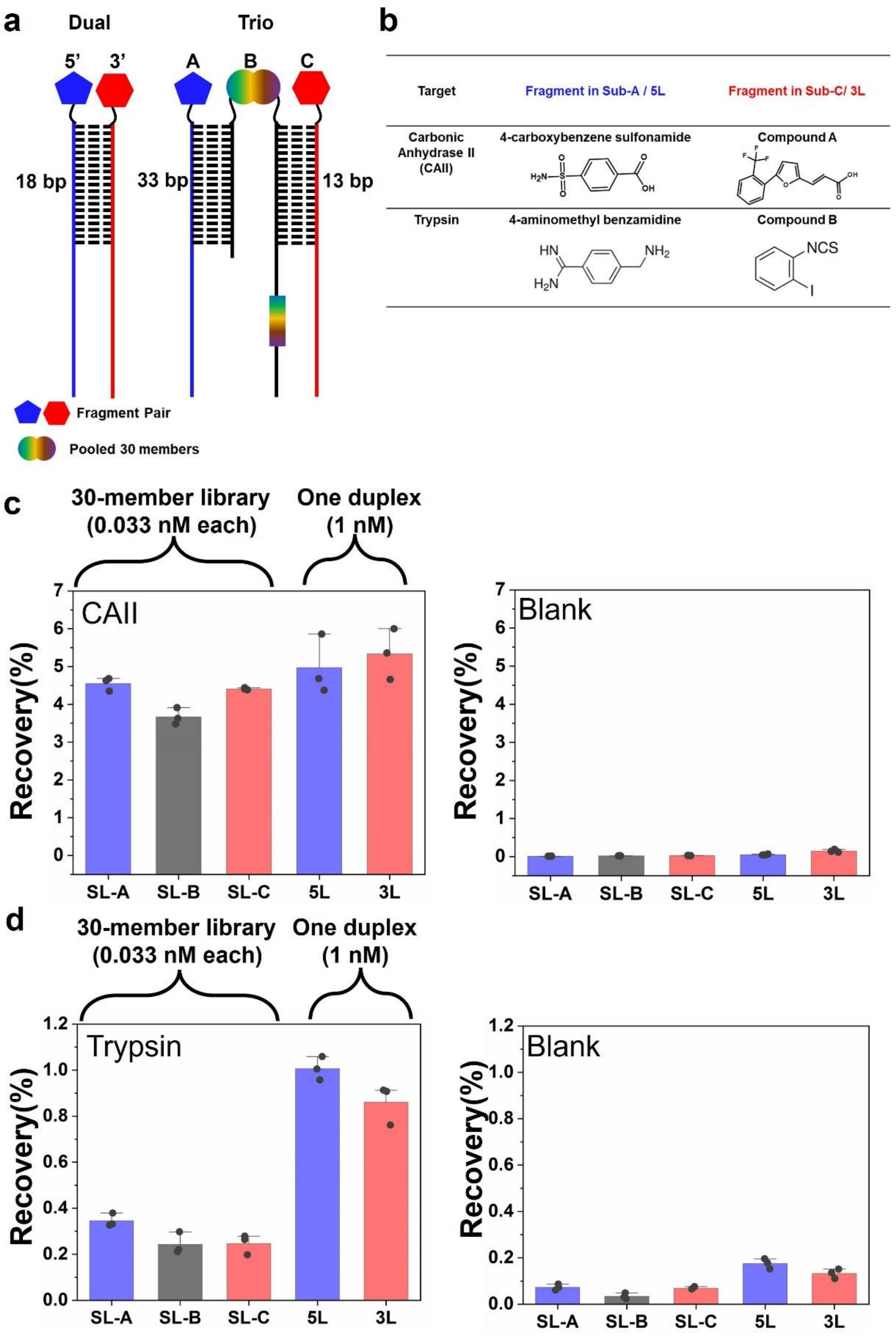

Supplementary Figure 8. Comparison of T-DEL format of 30 different combinations to the dual-pharmacophore format of one pair of ligands with model targets. (a) Schematic illustration of libraries in dual- and trio-pharmacophore formats. The ligand pairs were displayed as single-member sub-libraries, respectively. In T-DEL format, the two sub-libraries A and C were assembled with the 30-member sub-library B. (b) The ligand pairs of CAII and trypsin is shown in the table. (c) Recovery of each sub-library from the affinity selection against CAII (left) and

blank solid support (right). Data are presented as mean values +/- SD from three independent selection experiments. (n=3, three biological replicates) (d) Recovery of each sub-library from the affinity selection against trypsin (left) and blank solid support (right). SL-A, SL-B, and SL-C are from T-DEL format and 5L and 3L are from dual-pharmacophore format. Data are presented as mean values +/- SD from three independent measurements. Data are presented as mean values +/- SD from three independent selection experiments. n=3 biological replicates. Source data are provided as a Source Data file.

Further, we utilized Alpha-1-acid glycoprotein (AGP) and its reported two ligand pairs[3,4]: biphenyl-2-yl-(toluene-4-sulfonyl)-amino)-acetic acid (Compound C) paired with 3-[5-(2-(Trifluoromethyl)phenyl)furan-2-yl]-acrylic acid (Compound A) and Compound C paired with 3-[5-(2-(Trifluoromethyl)phenyl)furan-propanoic acid (Compound D) whose affinity differed by one order of magnitude in an on-DNA binding assay by Octet biolayer interferometry (Supplementary Fig. 9).

a

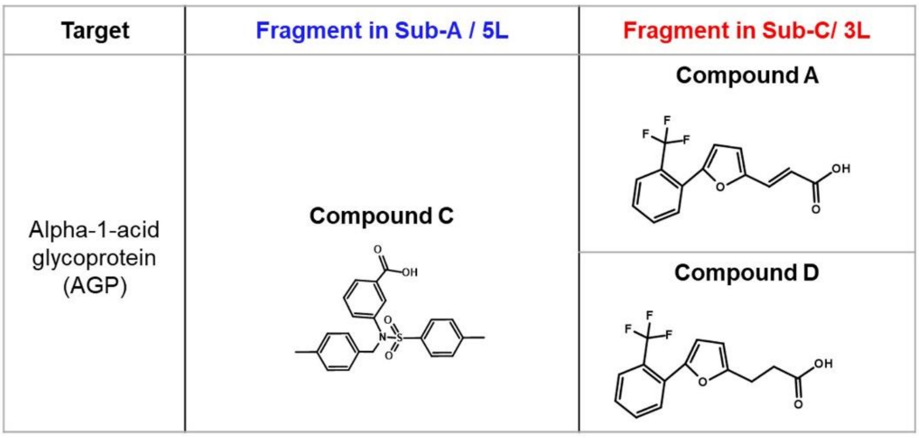

b

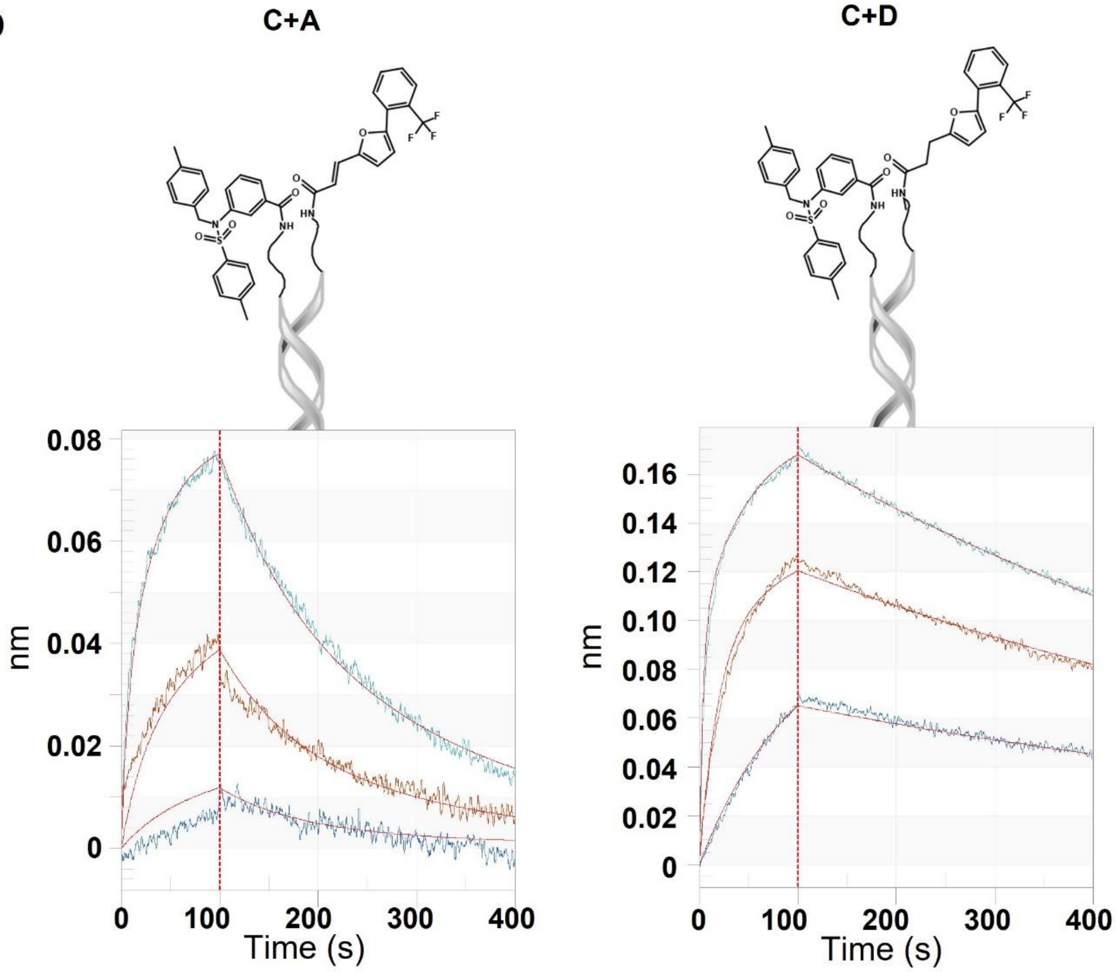

	Compound C + Compound A	Compound C + Compound D
K_d	8.733 ± 3.291 μM	0.230 ± 0.001 μM
K_{on}	$(4.625 \pm 0.285) \times 10^3\ M^{-1}\ s^{-1}$	$(5.044 \pm 0.060) \times 10^3\ M^{-1}\ s^{-1}$
K_{off}	$(4.039 \pm 0.174) \times 10^{-3}\ s^{-1}$	$(1.161 \pm 0.018) \times 10^{-3}\ s^{-1}$

Supplementary Figure 9. Alpha-1-acid-glycoprotein (AGP) as a model for evaluating DELs in different formats. (a) The reported two ligand pairs binding to AGP. (b) On-DNA affinity

measurements by Octet Biolayer Interferometry. The two compounds of each ligand pair were displayed at the 5’ and 3’ termini of a dsDNA, respectively. The construct was immobilized on the Octet Biolayer Interferometry sensor, and the binding of AGO to the sensor surface was measured, which led to the determination of K_{on} and K_{off} values.

Molecular docking studies revealed that compound C was binding to the same site as the reported ligand (PDB: 3KQ0). When the two compounds of the ligand pairs were docked together to AGP, A or D occupied a sub-site adjacent to that of C. Both ligand pairs displayed the same binding mode, while C+D exhibited a higher docking score, presumably due to the rotatable single bond of D compared to A (Supplementary Fig. 10 and 12).

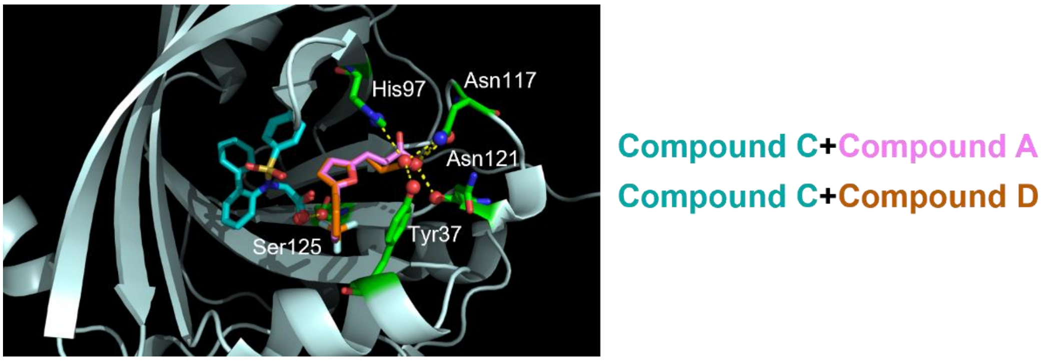

Supplementary Figure 10. Simultaneous docking of the ligand pairs (C+A and C+D) in complex with AGP (PDB: "3KQ0 [https://www.rcsb.org/structure/3KQ0]"). Yellow dashed lines indicate hydrogen bonds.

With the two ligand pairs binding to the same target with different affinities, we again measured the recoveries from the selections in dual- and trio-formats (Supplementary Fig. 11). As shown in Supplementary Fig. 9 and 11, compounds C and D exhibited approx. 40 times higher affinity than the other pair in the binding assay and recovered higher in the selection in both formats. The dual pharmacophore format led to a higher recovery of the ligand pair compounds C and A, while both formats showed comparable enrichment with the high-affinity pair compounds C and D. As expected, we have observed that with the increase of binding affinity of ligands in SL-A and SL-C, the overall contributions from SL-B on binding can be augmented, as shown by the ligand-dependent enhancement of recovery in the T-DEL format. The signals from T-DEL selections are the averages of 30 different combinations, while the concentration of each is 0.033 nM, in contrast to the 1 nM of the dual pharmacophore format. Therefore, we further measured the enrichment of

each sub-library B member and found that some members were selectively enriched in target selection but not in blank control selection. Further, in the T-DEL selections with either C+A or C+D we observed overlapping hits from SL-B (16, 19, and 20) (Supplementary Fig. 11c and 11d).

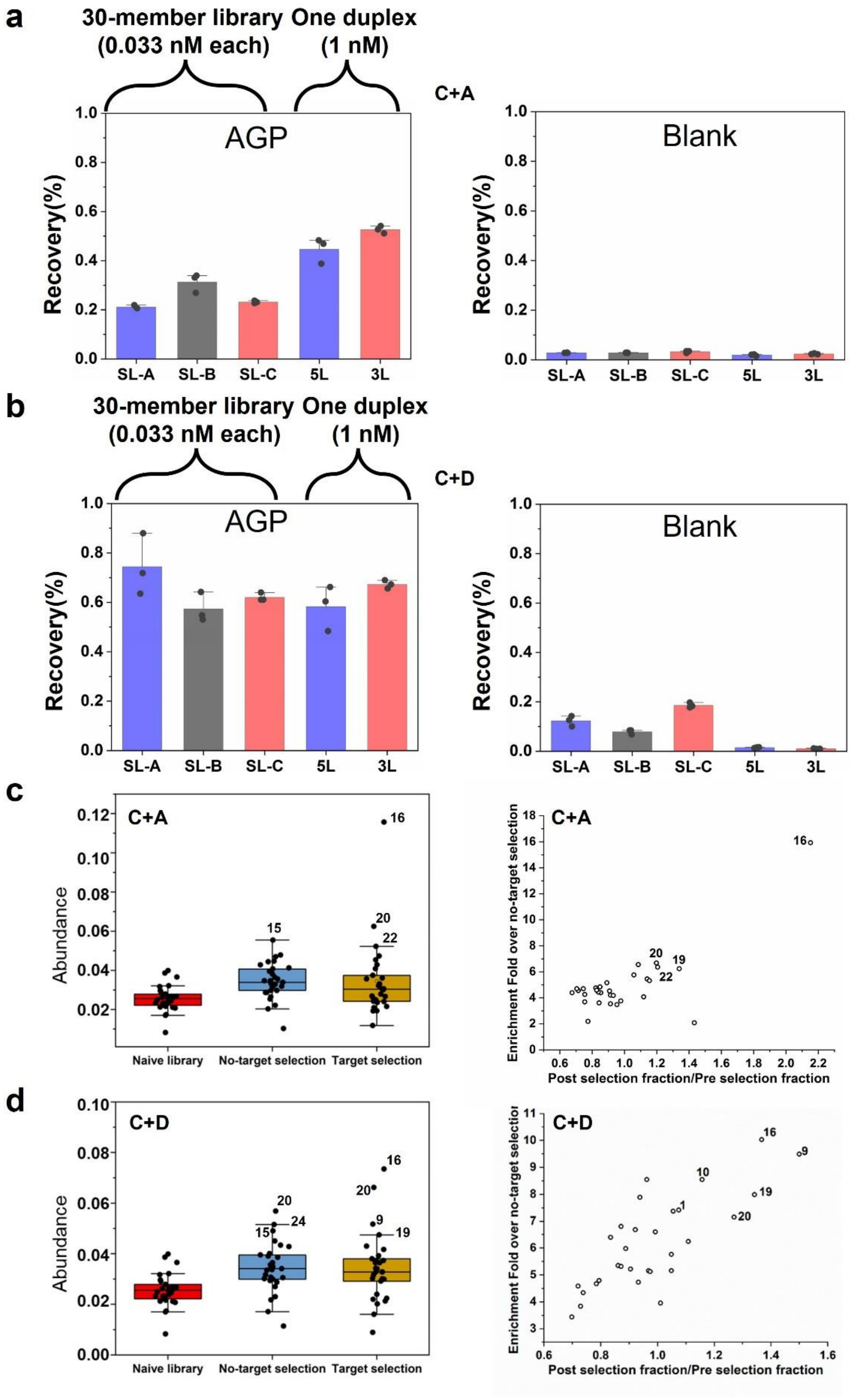

Supplementary Figure 11. Comparison of T-DEL format to the dual-pharmacophore format with alpha-1-acid glycoprotein (AGP). (a) The ligand pair Compound C and Compound A served as SL-A/5L and SL-C/3L, respectively. SL-A and SL-C were assembled with 30-member SL-B to form the T-DEL. Data are presented as mean values +/- SD from three

independent selection measurements. n=3 biological replicates. (b) The ligand pair Compound C and Compound D served as SL-A/5L and SL-C/3L, respectively. SL-A and SL-C were assembled with 30-member SL-B to form the T-DEL. Data are presented as mean values +/- SD from three independent selection measurements. n=3 biological replicates (c and d) The abundance of each member in SL-B in naïve pre-selection library, no-target selection, and target selection (left). Members of pronouncing abundance or enrichment are labeled with their IDs. Enrichment profile of each member in SL-B by normalizing to naïve library and no-target selection (right). Abundance was calculated by dividing the recovered amount of each member by the total recovered amount of SL-B. The Enrichment Fold was calculated in two steps. First, the amount of total SL-B from target selection was divided by the amount of total SL-B from no-target selection to calculate the enrichment factor (F). Second, the abundance of each member from target selection was divided by the abundance of each member from no-target selection, and the obtained value was multiplied by F to deliver the Enrichment Fold. The experiments were performed three times independently (n=3). The boxes range from the 25th to 75th percentile. The whiskers range from 10th to 90th percentile. Horizontal lines inside the boxes indicate the median. Source data are provided as a Source Data file.

With the selected hits 16, 19, and 20, we virtually synthesized six linked compounds (C-16-A, C-19-A, C-20-A, C-16-D, C-19-D, and C-20-D) and performed molecular docking. All compounds were binding to the same binding site as C+A and C+D. In particular, C-16-A and C-16-D displayed higher docking scores and larger hydrophobic contacts than compounds linked by 19 and 20 (Supplementary Fig. 12).

a

	Docking Score	Hydrophobic Contacts	H-bonds	TYR_37	THR_47	GLU_64	ARG_90	GLY_93	HIS_97	ASN_117	ASN_121	SER_125	TYR_127
C+A	-13.43	164	4	0	0	0	0	0	0	2	1	1	0
C+D	-13.77	168	6	1	0	0	0	0	1	2	1	1	0
C-16-A	-11.29	159	2	0	0	0	1	0	0	0	0	0	1
C-19-A	-9.729	87	2	0	0	0	0	1	0	0	0	0	1
C-20-A	-9.57	138	2	0	0	0	1	1	0	0	0	0	0
C-16-D	-10.97	204	1	0	0	0	0	0	0	0	0	1	0
C-19-D	-9.074	98	2	0	0	0	0	1	0	0	0	0	1
C-20-D	-10.01	156	1	0	1	0	0	0	0	0	0	0	0

b

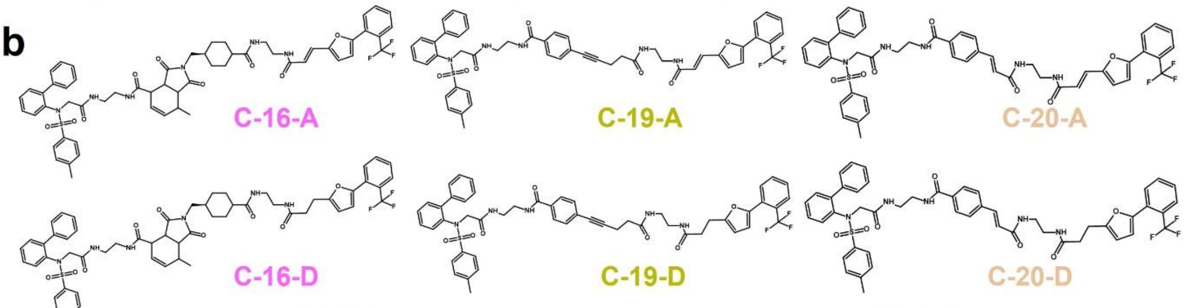

c

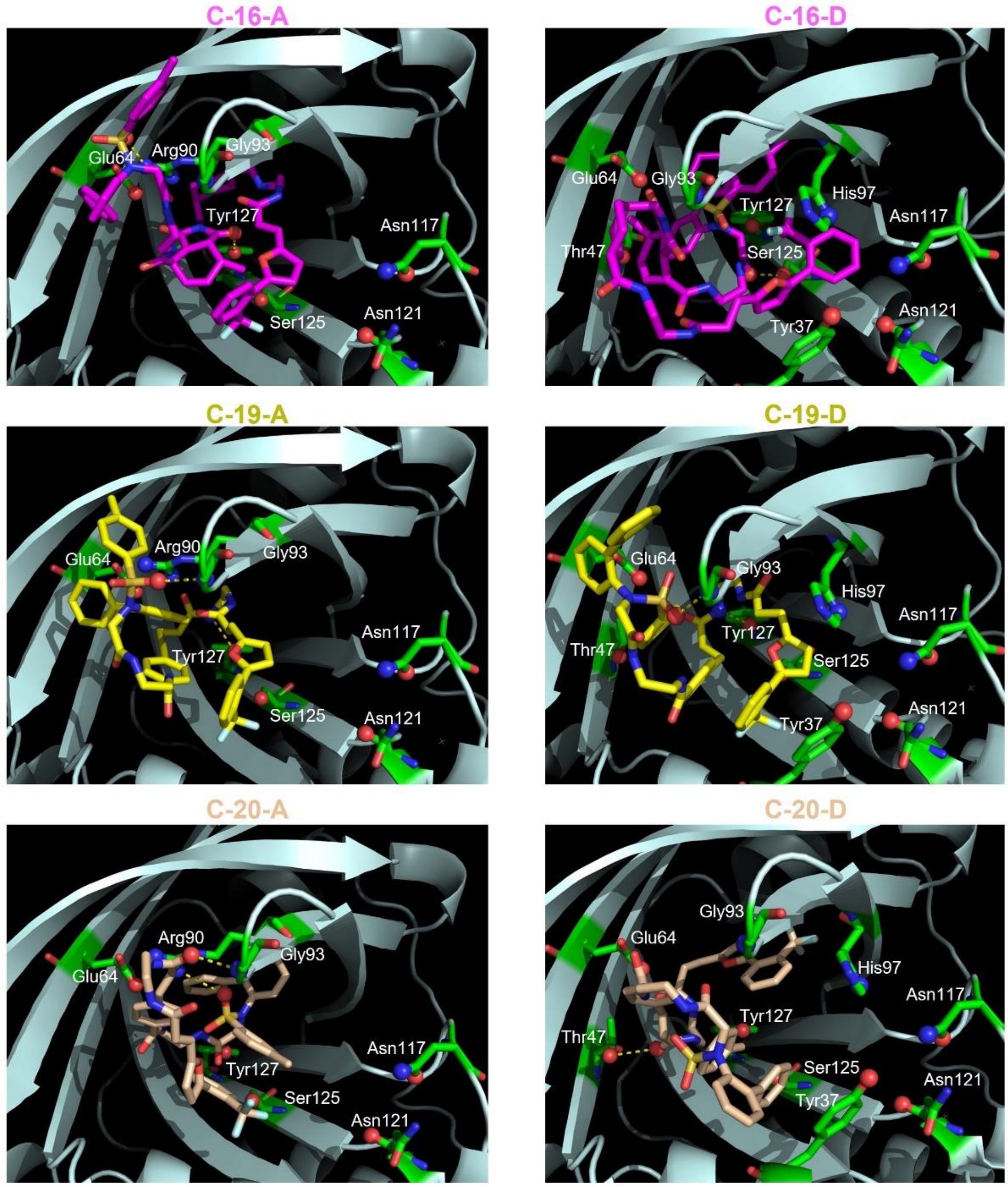

Supplementary Figure 12. (a) Summary of interactions between small molecule compounds and Alpha-1-acid glycoprotein (AGP) (PDB: "3KQ0

[https://www.rcsb.org/structure/3KQ0]"). (b) Structures of the virtually synthesized compounds linked by SL-B members 16, 19, and 20. (c) Docking poses of the compounds in complex with AGP. Yellow dashed lines indicate hydrogen bonds.

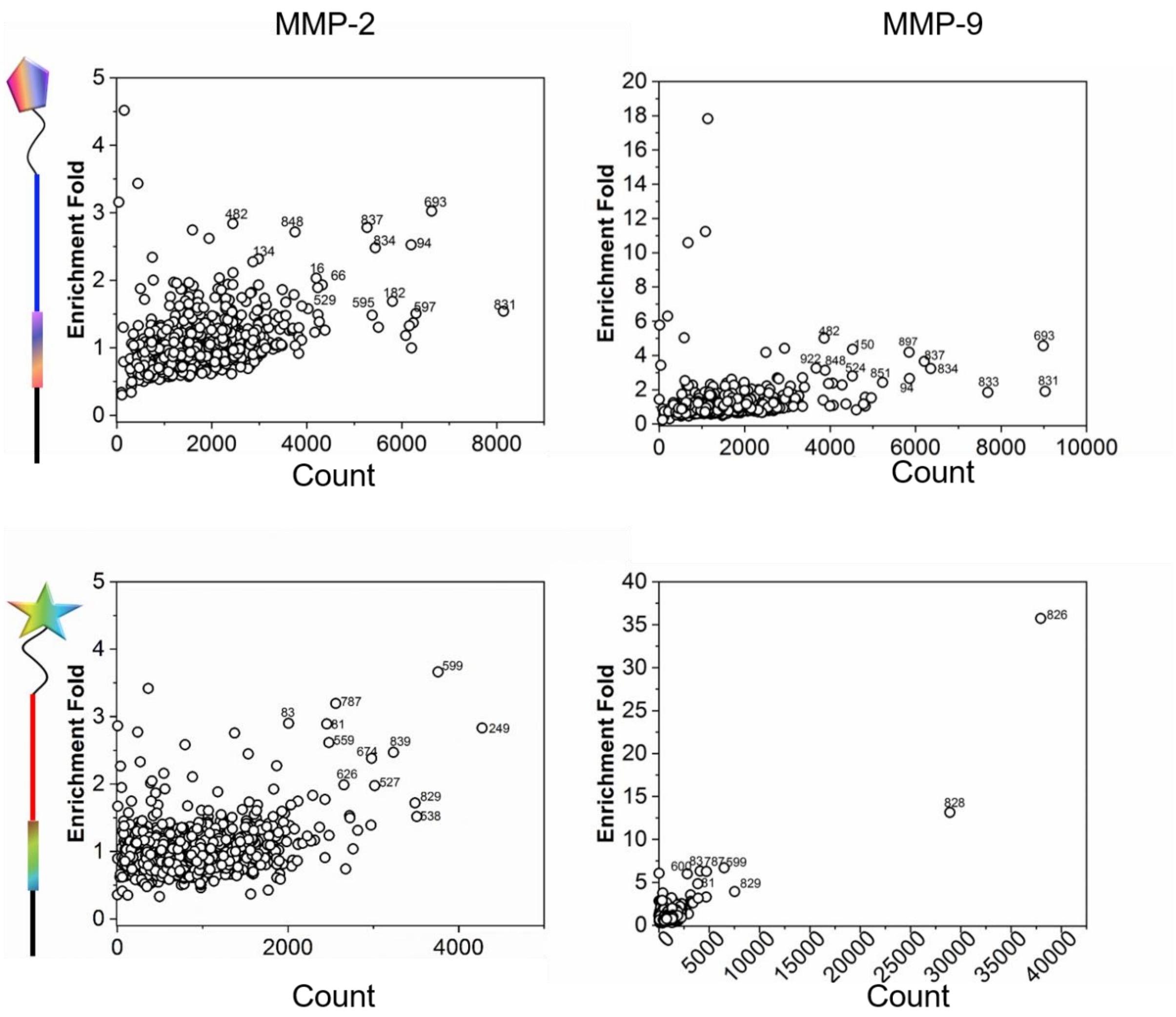

Supplementary Figure 13. Scatter plots of de novo selection against MMP-2 and MMP-9 using the 883 x 890-member dual pharmacophore DEL. Hits enriched from trio-pharmacophore format were also identified from the dual-pharmacophore format. Source data are provided as a Source Data file.

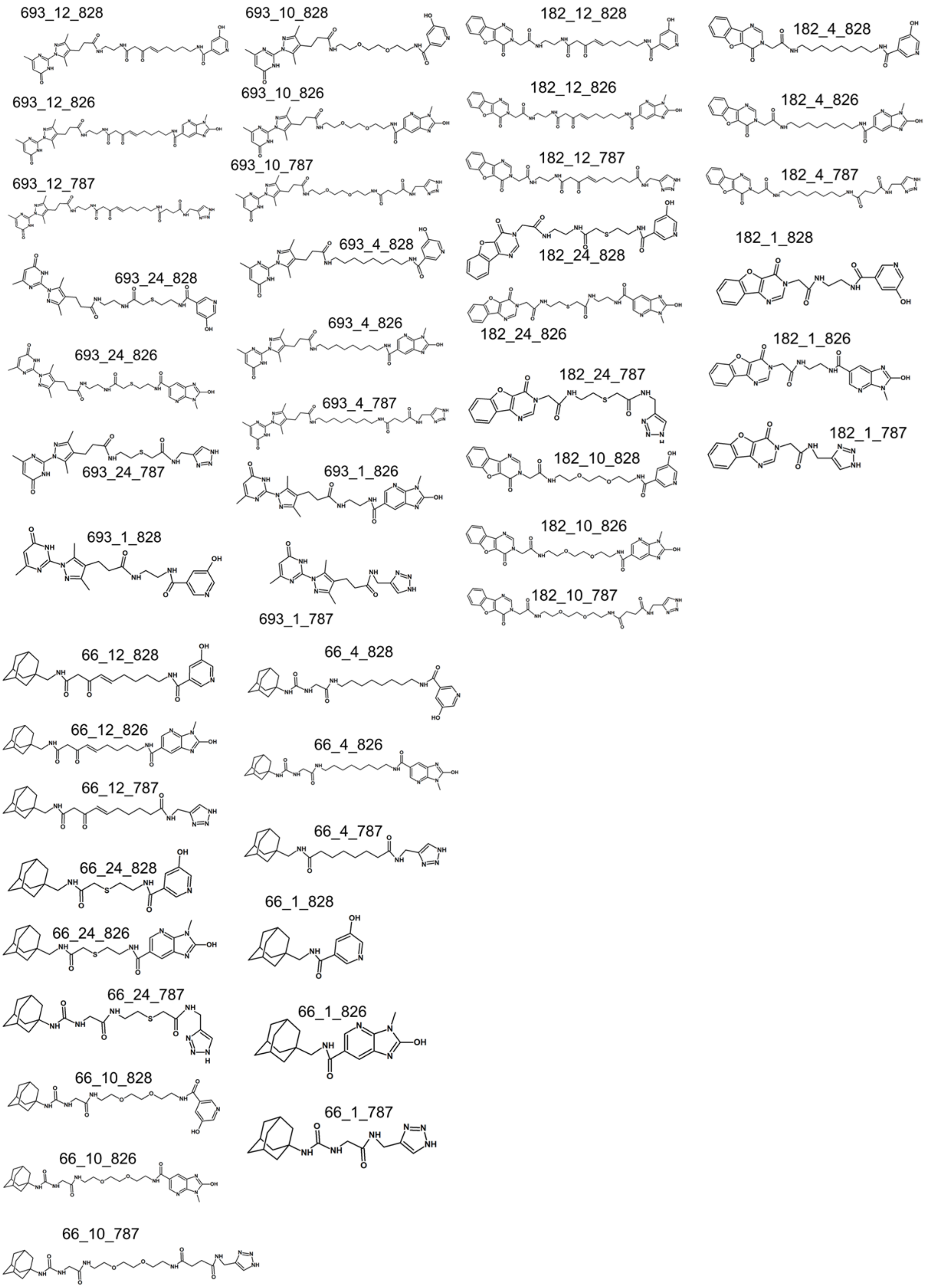

Supplementary Figure 14. Structure of 45 re-synthesized small molecule compounds.

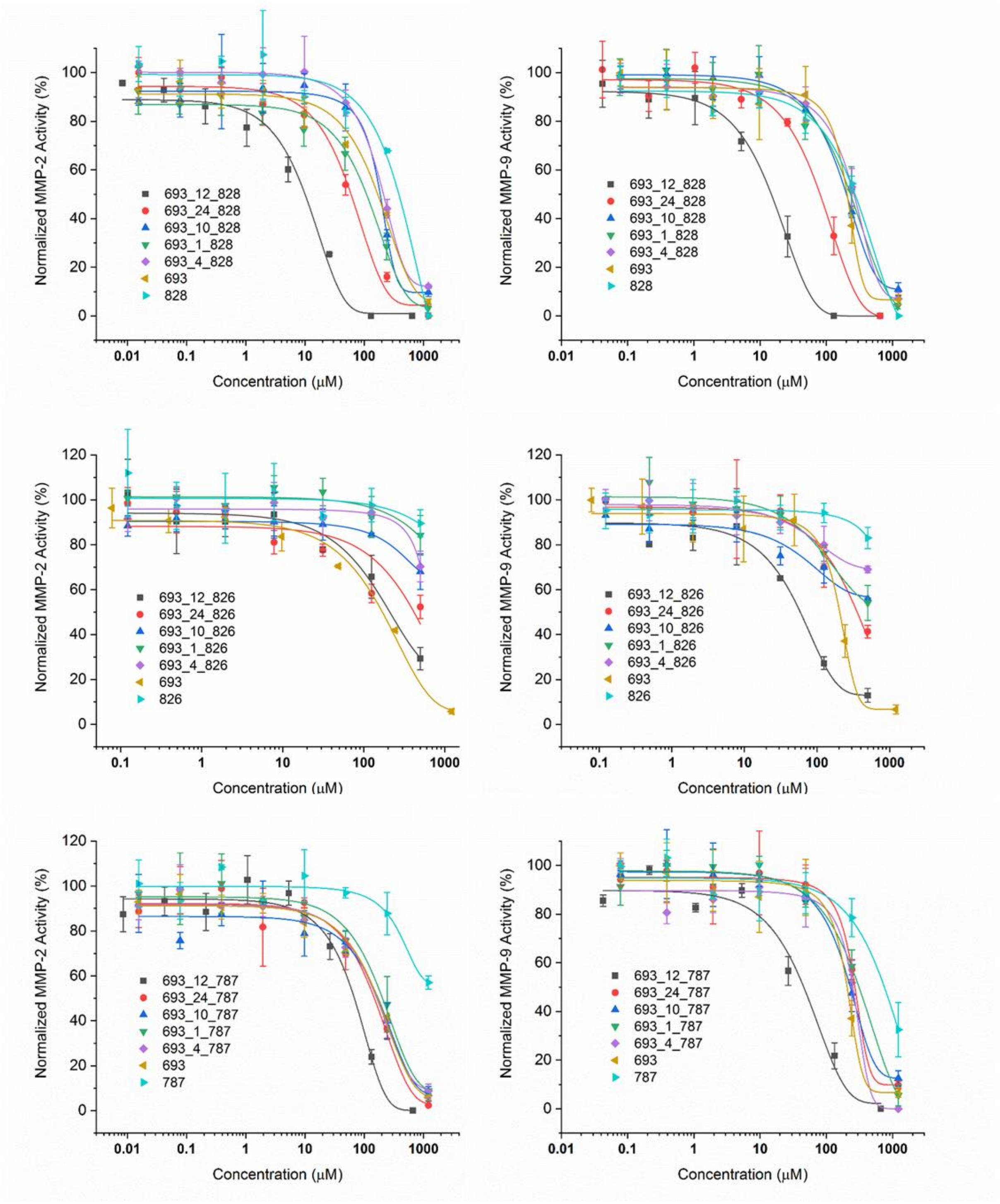

Supplementary Figure 15. Dose dependent inhibition of MMP-2 (left) and MMP-9 (right) by compound combinations 693+828, 693+826, 693+787, and single fragments. Data are presented as mean values +/- SD from three independent measurements (n=3 biological replicates). Source data are provided as a Source Data file.

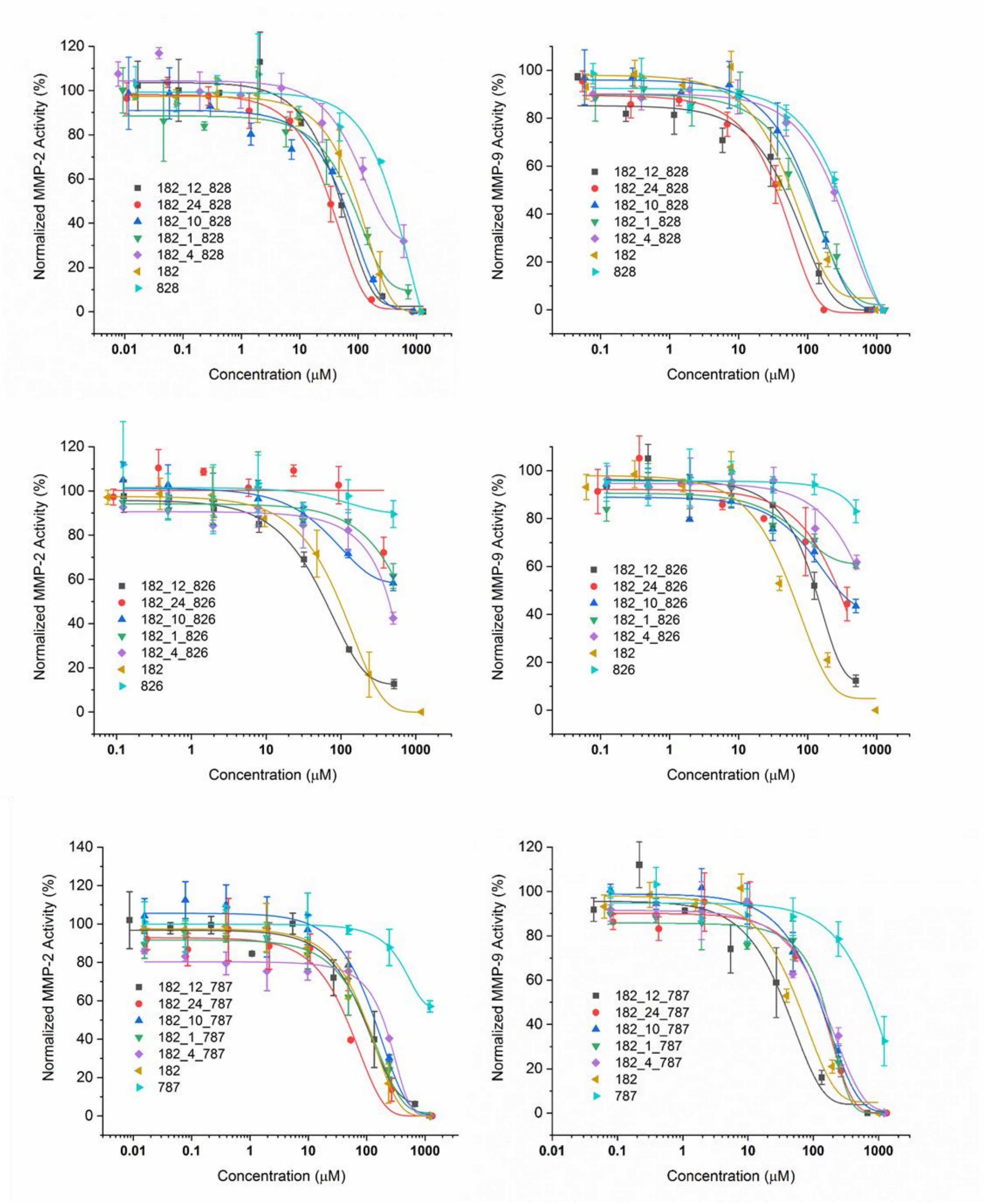

Supplementary Figure 16. Dose dependent inhibition of MMP-2 (left) and MMP-9 (right) by compound combinations 182+828, 182+826, 182+787, and single fragments. Data are presented as mean values +/- SD from three independent measurements (n=3 biological replicates). Source data are provided as a Source Data file.

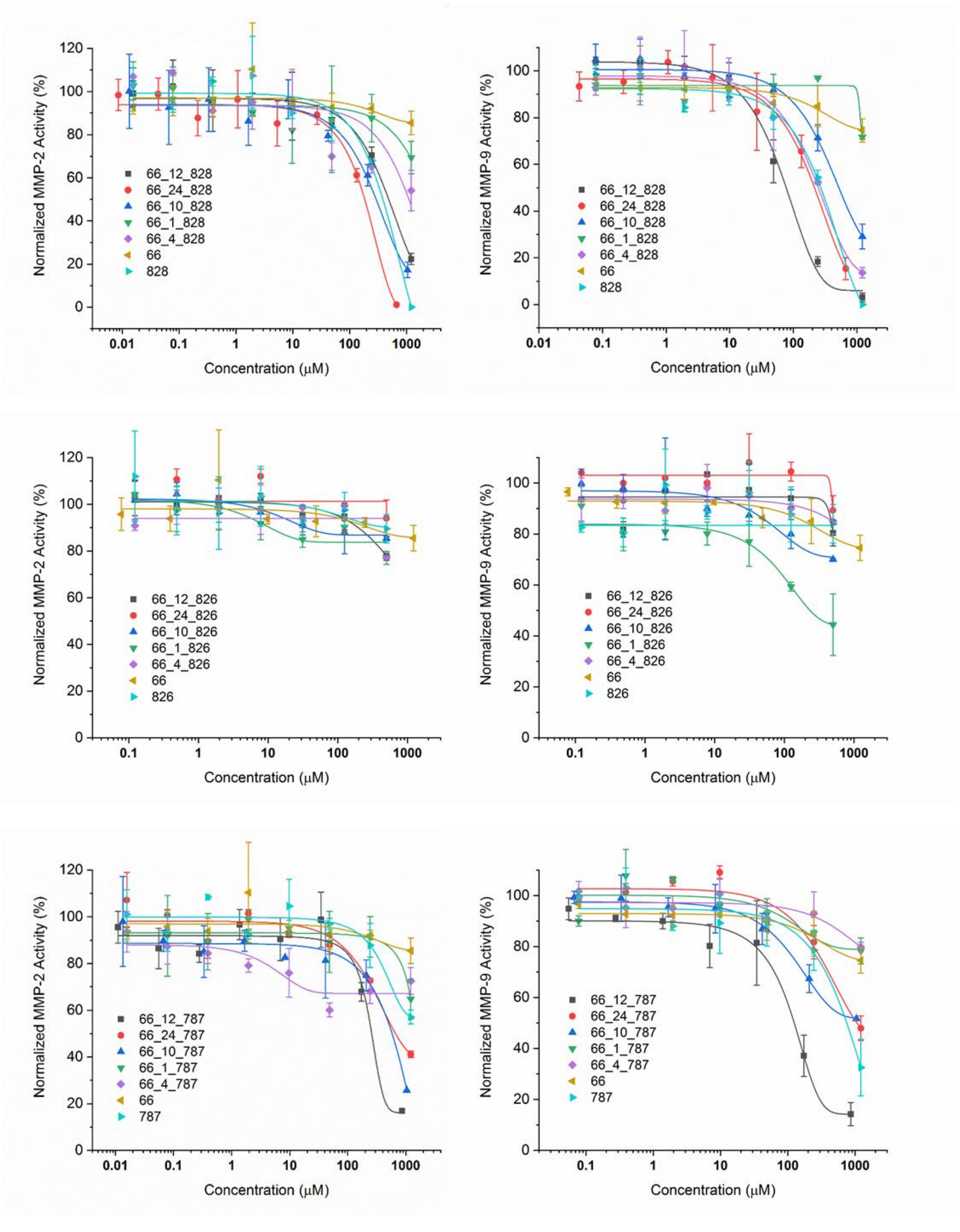

Supplementary Figure 17. Dose dependent inhibition of MMP-2 (left) and MMP-9 (right) by compound combinations 66+828, 66+826, 66+787, and single fragments. Data are presented as mean values +/- SD from three independent measurements (n=3 biological replicates). Source data are provided as a Source Data file.

Supplementary Discussion 4

Analysis on docking studies with MMP-2/-9 and the ligands

The binding mode of compounds in the groups of 182+828 and 693+828 were analyzed by molecular docking. All compounds displayed binding to the S1' subsite of the substrate binding cleft of the catalytic domain by inserting the 182 or 693 moiety, and the 828 moiety was interacting with the catalytic Zn^{2+} ion. The binding mode of the compound to MMP-2 and MMP-9 showed high similarity, presumably due to their high structural similarity in the catalytic domains, agreeing with the measured inhibitory effects against both enzymes (Supplementary Fig. 18-22).

S1' subsite is also known as the specificity pocket in MMPs because the S1' loops among MMP isozymes share little sequence and length similarity but maintain hydrophobic features[5]. S1' subsite in MMP-2 or MMP-9 is described as a tunnel-like cavity, while it is found to be smaller in collagenases and matrilysins[5-7]. Therefore, targeting S1' subsite represents a promising strategy for developing selective MMP inhibitors[5]. The hydrophobic nature of the S1' pocket is suited to accommodate aromatic groups and ring systems[9], as was also demonstrated by our docking studies.

a

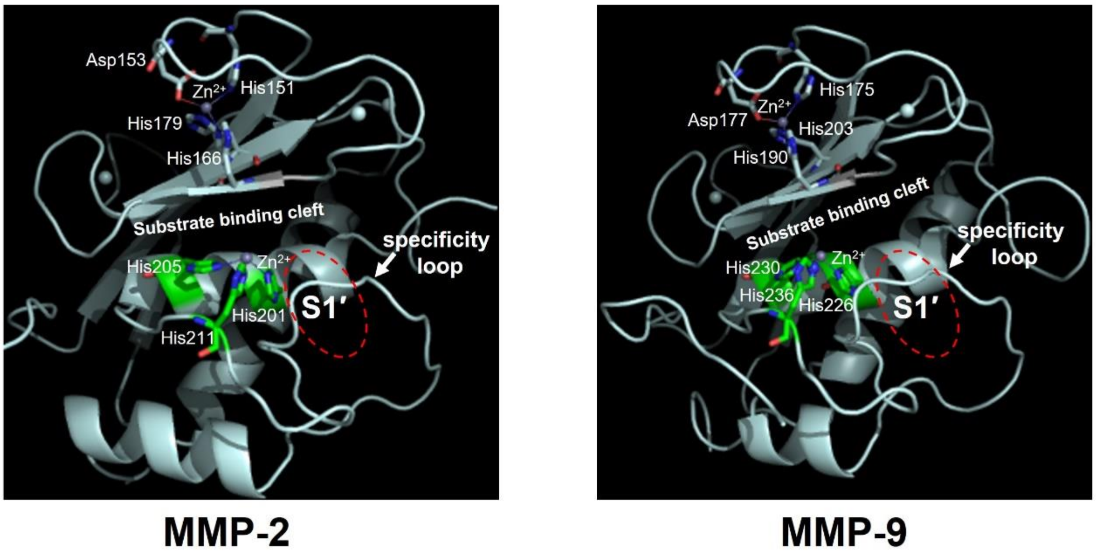

b

MMP-2	Docking Score	Hydrophobic Contacts	H-bonds	Pro221	Ala165	Ala167	Leu164	Ala220	Gly162	Tyr223
182+828	-15.85	90	5	0	2	2	1	0	0	0
182_1_828	-13.04	81	5	1	2	0	2	0	0	0
182_4_828	-12.84	112	1	0	0	0	1	0	0	0
182_10_828	-12.67	92	4	0	1	1	2	0	0	0
182_12_828	-12.57	99	3	0	1	1	0	0	0	0
182_24_828	-12.62	98	3	0	1	1	1	0	0	0
693+828	-17.13	89	0	2	1	0	1	0	0	0
693_1_828	-13.51	90	0	1	0	1	0	0	0	0
693_4_828	-13.62	106	0	1	1	0	1	0	0	0
693_10_828	-13.08	96	0	0	1	1	1	0	0	0
693_12_828	-13.52	112	0	0	1	1	1	0	0	1
693_24_828	-13.74	97	1	0	1	0	1	1	0	0

c

MMP-9	Docking Score	Hydrophobic Contacts	H-bonds	Tyr245	Ala189	Glu227	Ala191	Leu188	Pro246
182+828	-16.16	89	5	0	2	0	2	1	0
182_1_828	-13.35	87	2	0	1	0	0	1	0
182_4_828	-13.17	97	2	0	0	0	1	1	0
182_10_828	-12.91	90	3	0	1	0	1	1	0
182_12_828	-13.33	93	3	0	0	0	1	1	1
182_24_828	-12.99	91	2	0	0	0	1	1	0
693+828	-17.83	90	6	1	2	0	2	1	0
693_1_828	-14.18	100	3	1	1	0	0	1	0
693_4_828	-14.19	108	3	1	0	0	1	1	0
693_10_828	-13.43	91	4	1	1	0	1	1	0
693_12_828	-13.89	100	2	1	0	0	0	1	0
693_24_828	-13.8	100	3	1	0	0	1	1	0

Supplementary Figure 18. Molecular Docking of compounds in complex with MMP-2 (PDB: "1QIB [https://www.rcsb.org/structure/1QIB]") and MMP-9 (PDB: "4H3X [https://www.rcsb.org/structure/4H3X]"). (a) Structure of the catalytic domain of MMP-2 (left) and MMP-9 (right). The shallow substrate-binding cleft runs across the catalytic domain of MMP-2 and MMP-9, dividing the domain into the N-terminal upper subdomain and the lower C-terminal subdomain. Catalytic Zn^{2+} ion (dark grey sphere) lies on the surface of the catalytic cleft coordinated with three histidine residues in green, and the structural Zn^{2+} (dark grey

sphere) is in the upper subdomain chelating three histidine residues. Ca^{2+} ions are shown as light grey spheres. The proteins are in cartoon representation and some residues are highlighted in stick representation. The red dashed circle indicates the S1' subsite. (b) Summary of the interactions between compounds and MMP-2, in terms of docking score, hydrophobic contact, and hydrogen bonds (H-bonds). (c) Summary of the interactions between compounds and MMP-9 in terms of docking score, hydrophobic contact, and hydrogen bonds (H-bonds).

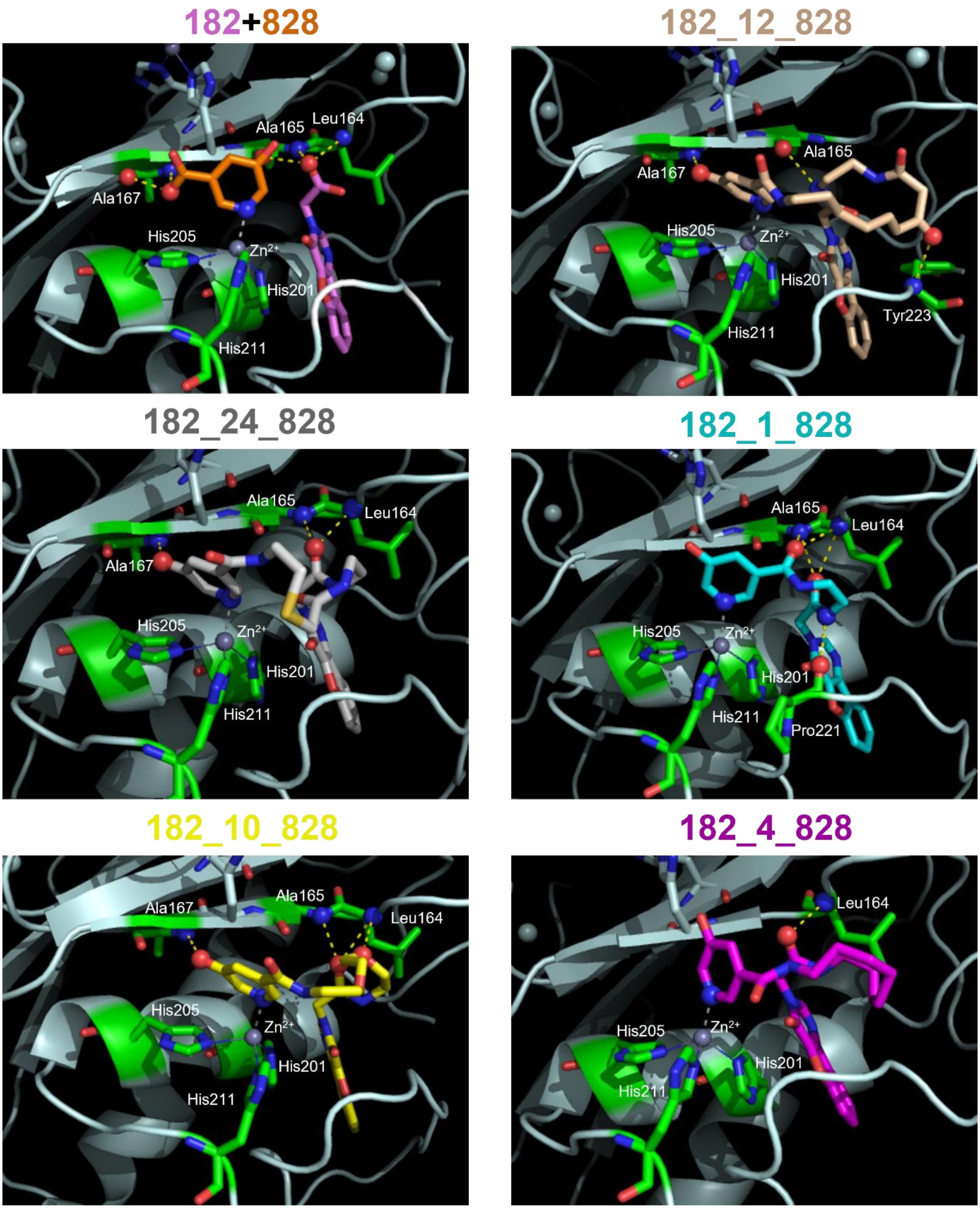

Supplementary Figure 19. Binding of compounds (**182+828**, **182_12_828**, **182_24_828**, **182_1_828**, **182_10_828**, and **182_4_828**) to the catalytic domain of MMP-2 (PDB: "1QIB [https://www.rcsb.org/structure/1QIB]"). Compounds are in stick model, the protein is in cartoon representation, and hydrogen bonding atoms are in ball representation. Zn^{2+} is shown as a sphere in dark grey and Ca^{2+} is shown as a sphere in light grey. The coordination of Zn^{2+} and **828** is shown as a grey dashed line. Yellow dashed lines indicate hydrogen bonds.

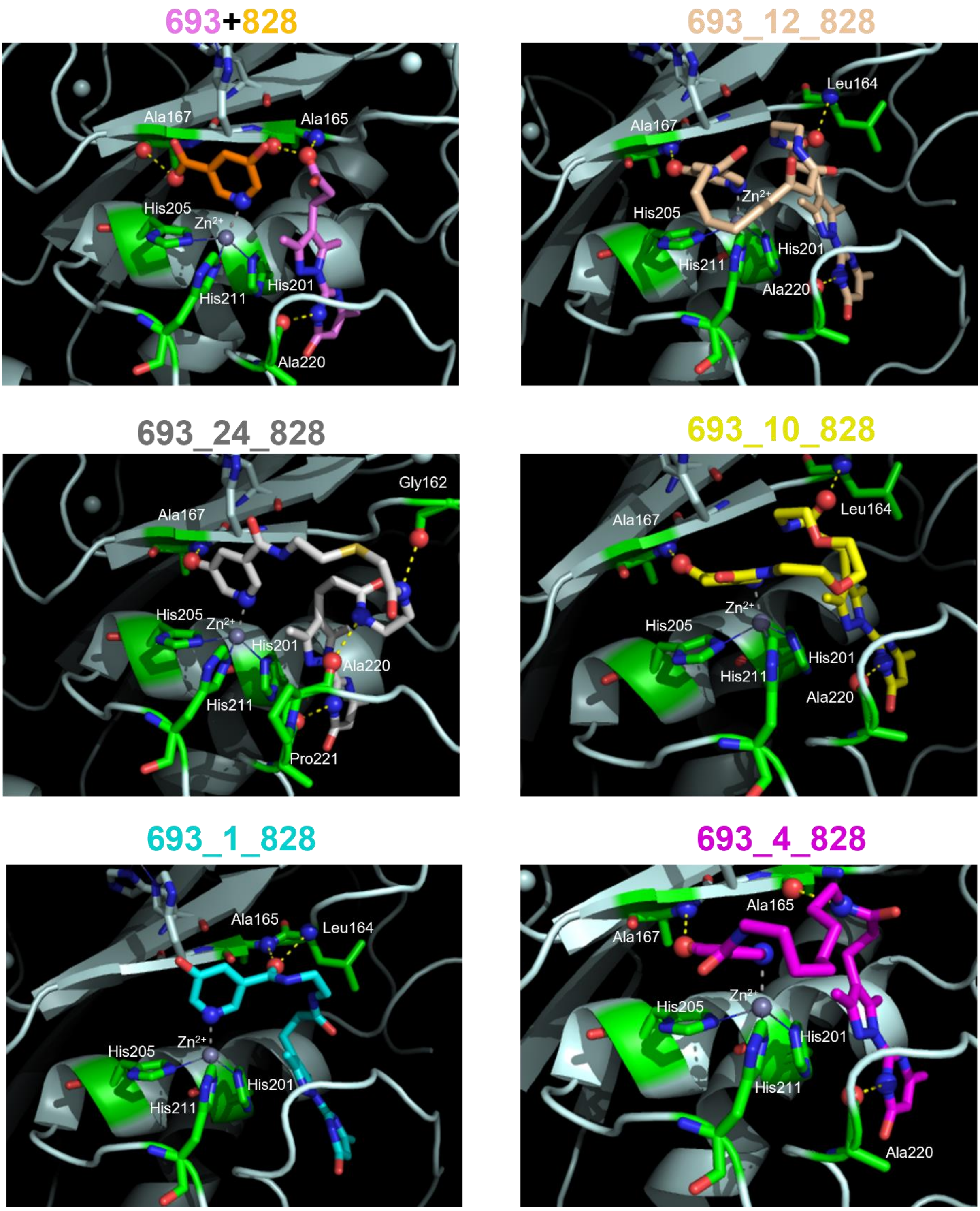

Supplementary Figure 20. Binding of compounds (**693+828**, **693_12_828**, **693_24_828**, **693_1_828**, **693_10_828**, and **693_4_828**) to the catalytic domain of MMP-2 (PDB: "1QIB [https://www.rcsb.org/structure/1QIB]"). Compounds are in stick model, the protein is in cartoon representation, and the hydrogen bonding atoms are in ball representation. Zn^{2+} is shown as a sphere in dark grey and Ca^{2+} is shown as a sphere in light grey. The coordination of Zn^{2+} and **828** is shown as a grey dashed line. Yellow dashed lines indicate hydrogen bonds.

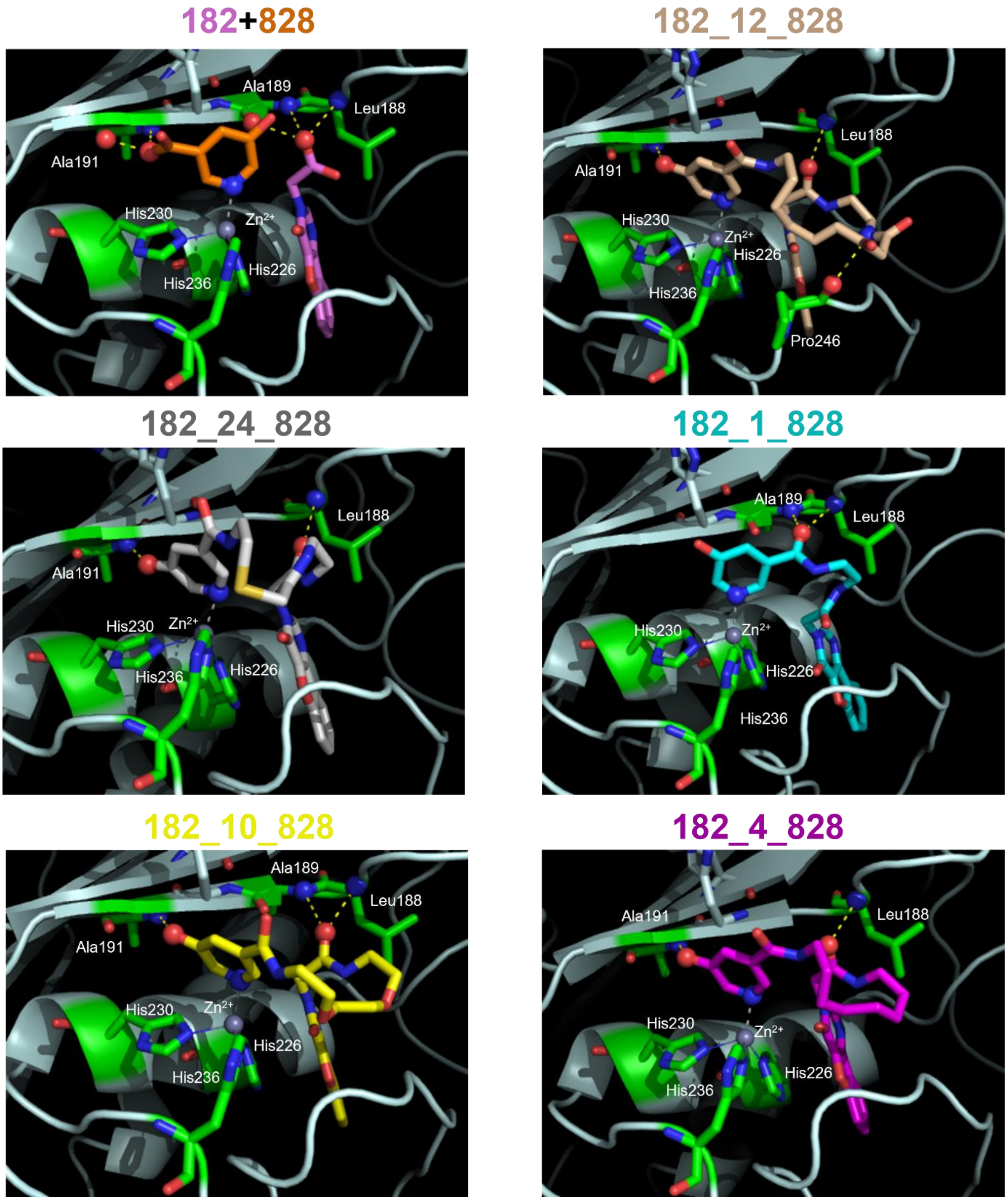

Supplementary Figure 21. Binding of compounds (**182+828**, **182_12_828**, **182_24_828**, **182_1_828**, **182_10_828**, and **182_4_828**) to the catalytic domain of MMP-9 (PDB: "4H3X [https://www.rcsb.org/structure/4H3X]"). Compounds are in stick model, the protein is in cartoon representation, and the hydrogen bonding atoms are in ball representation. Zn^{2+} is shown as a sphere in dark grey and Ca^{2+} is shown as a sphere in light grey. The coordination of Zn^{2+} and **828** is shown as a grey dashed line. Yellow dashed lines indicate hydrogen bonds.

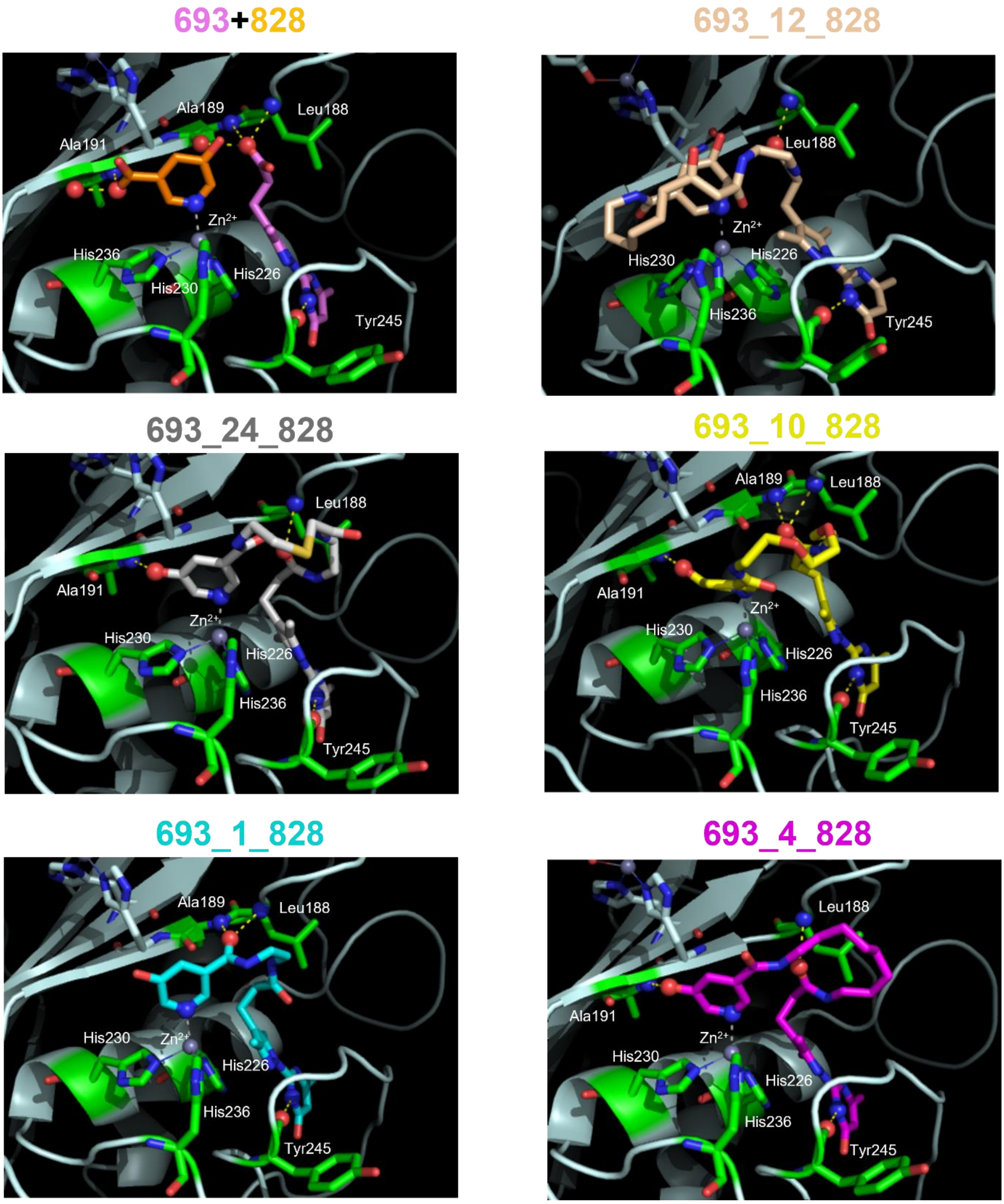

Supplementary Figure 22. Binding of compounds (**693+828**, **693_12_828**, **693_24_828**, **693_1_828**, **693_10_828**, and **693_4_828**) to the catalytic domain of MMP-9 (PDB: "4H3X [https://www.rcsb.org/structure/4H3X]"). Compounds are in stick model, the protein is in cartoon representation, and the hydrogen bonding atoms are in ball representation. Zn^{2+} is shown as a sphere in dark grey and Ca^{2+} is shown as a sphere in light grey. The coordination of Zn^{2+} and **828** is shown as a grey dashed line. Yellow dashed lines indicate hydrogen bonds.
.

Supplementary Note 1

Characterization of SL-B members by UPLC-ESI-MS

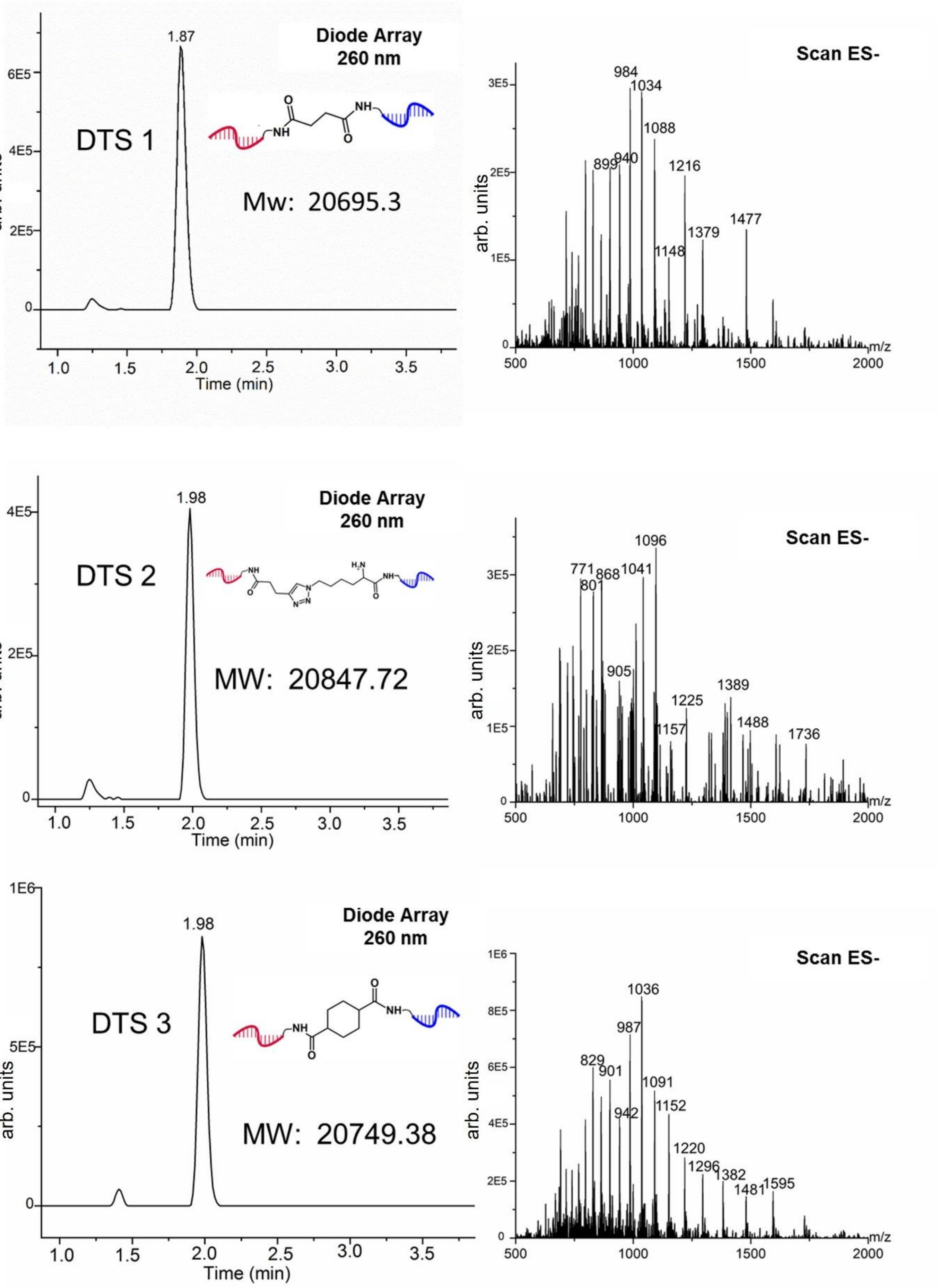

Supplementary Figure 23. UPLC chromatogram and ESI-MS validation of Sub-library members 1-3. ES: Electrospray Ionization Mode, arb. units: Arbitrary Units

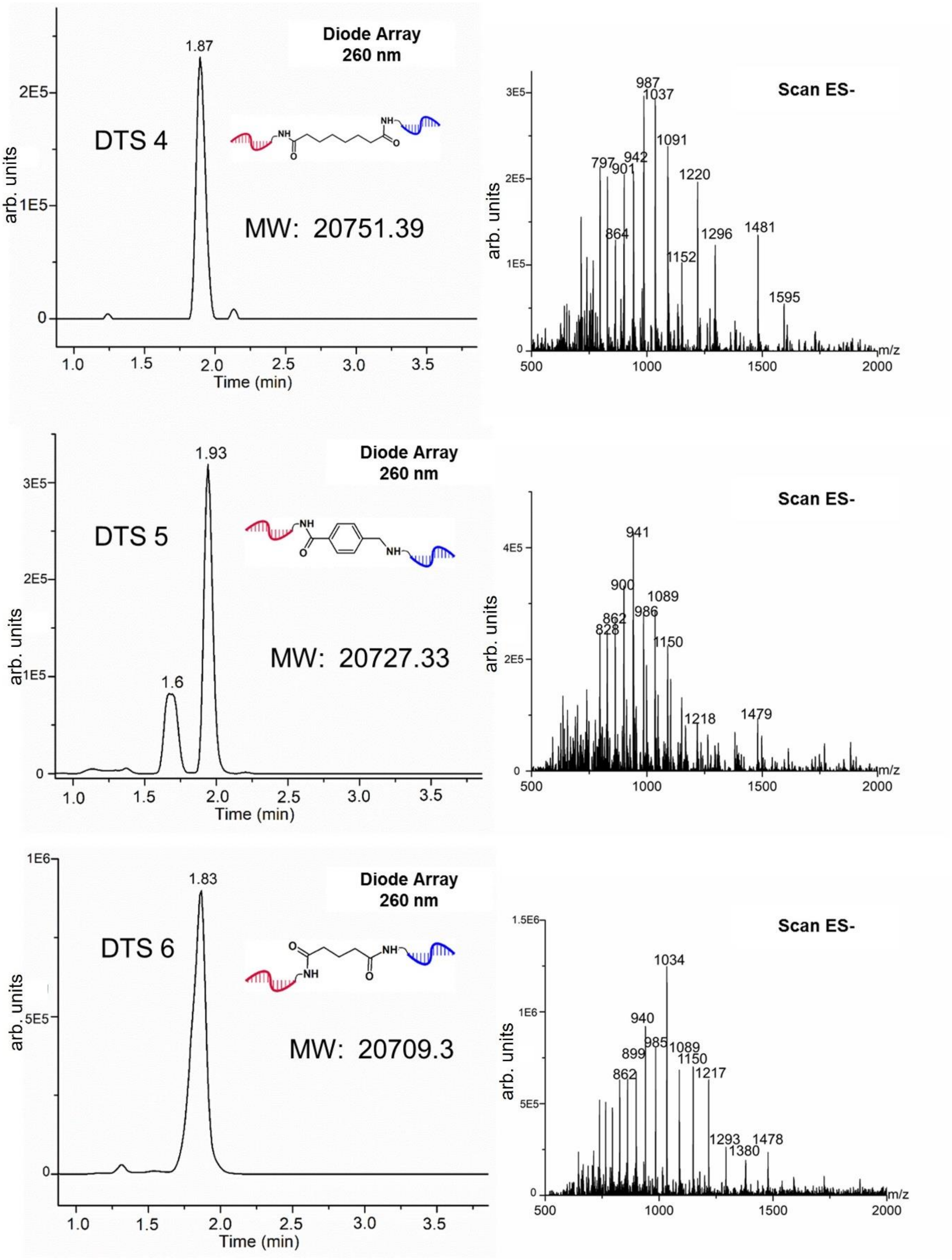

Supplementary Figure 24. UPLC chromatogram and ESI-MS validation of Sub-library members 4-6. ES: Electrospray Ionization Mode, arb units: Arbitrary Units.

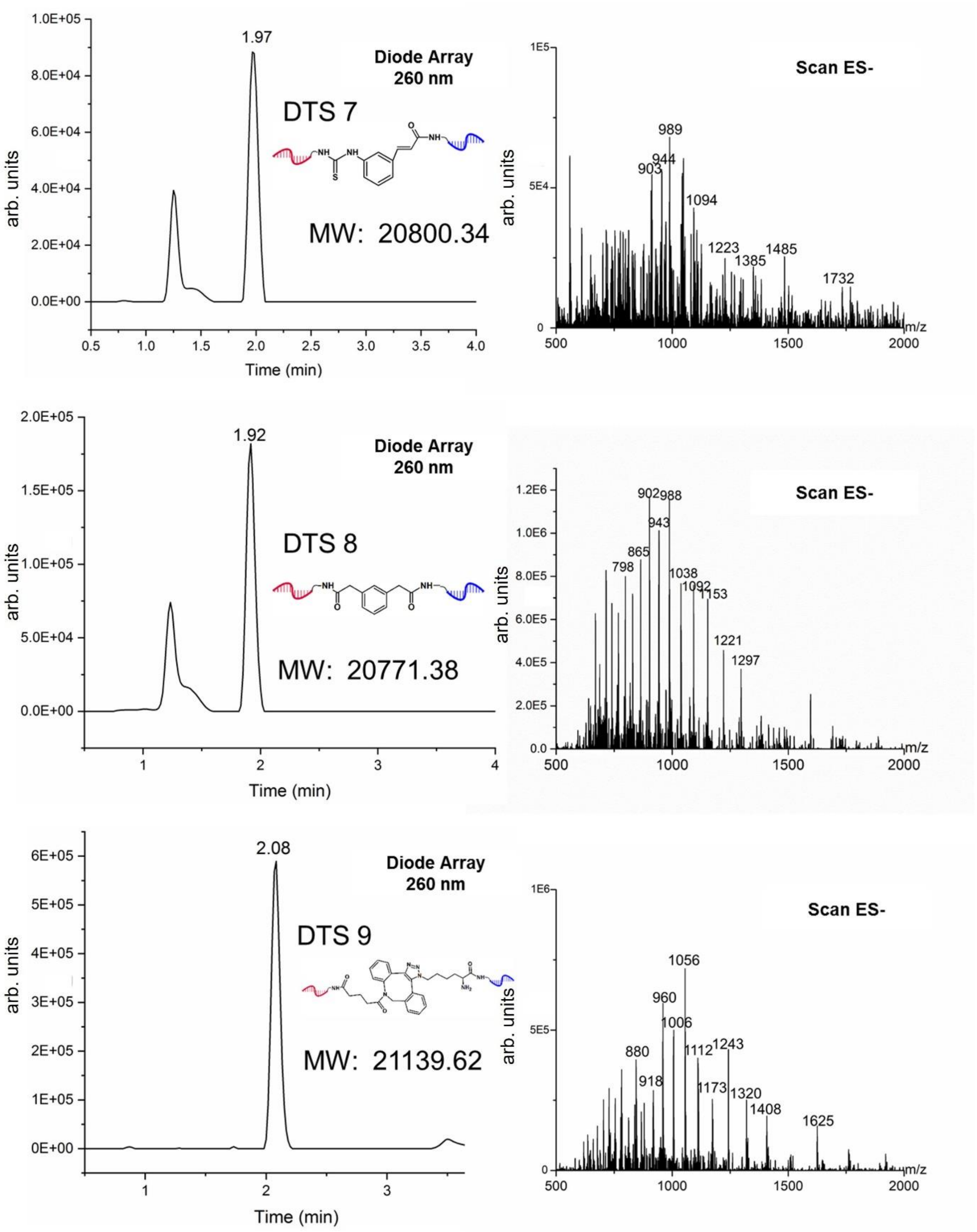

Supplementary Figure 25. UPLC chromatogram and ESI-MS validation of Sub-library members 7-9. ES: Electrospray Ionization Mode, arb.units: Arbitrary Units.

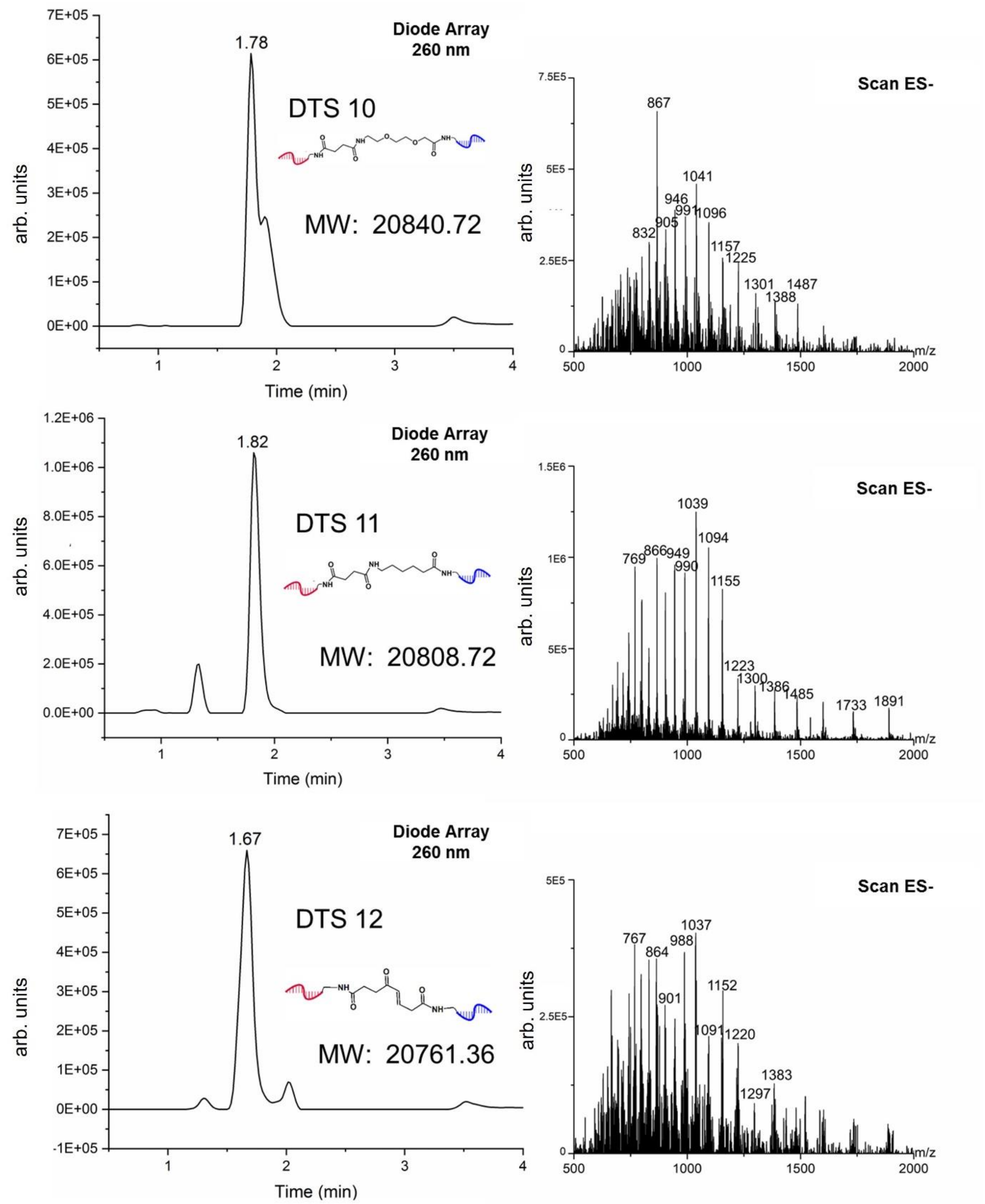

Supplementary Figure 26. UPLC chromatogram and ESI-MS validation of Sub-library members 10-12. ES: Electrospray Ionization Mode, arb. units: Arbitrary Units.

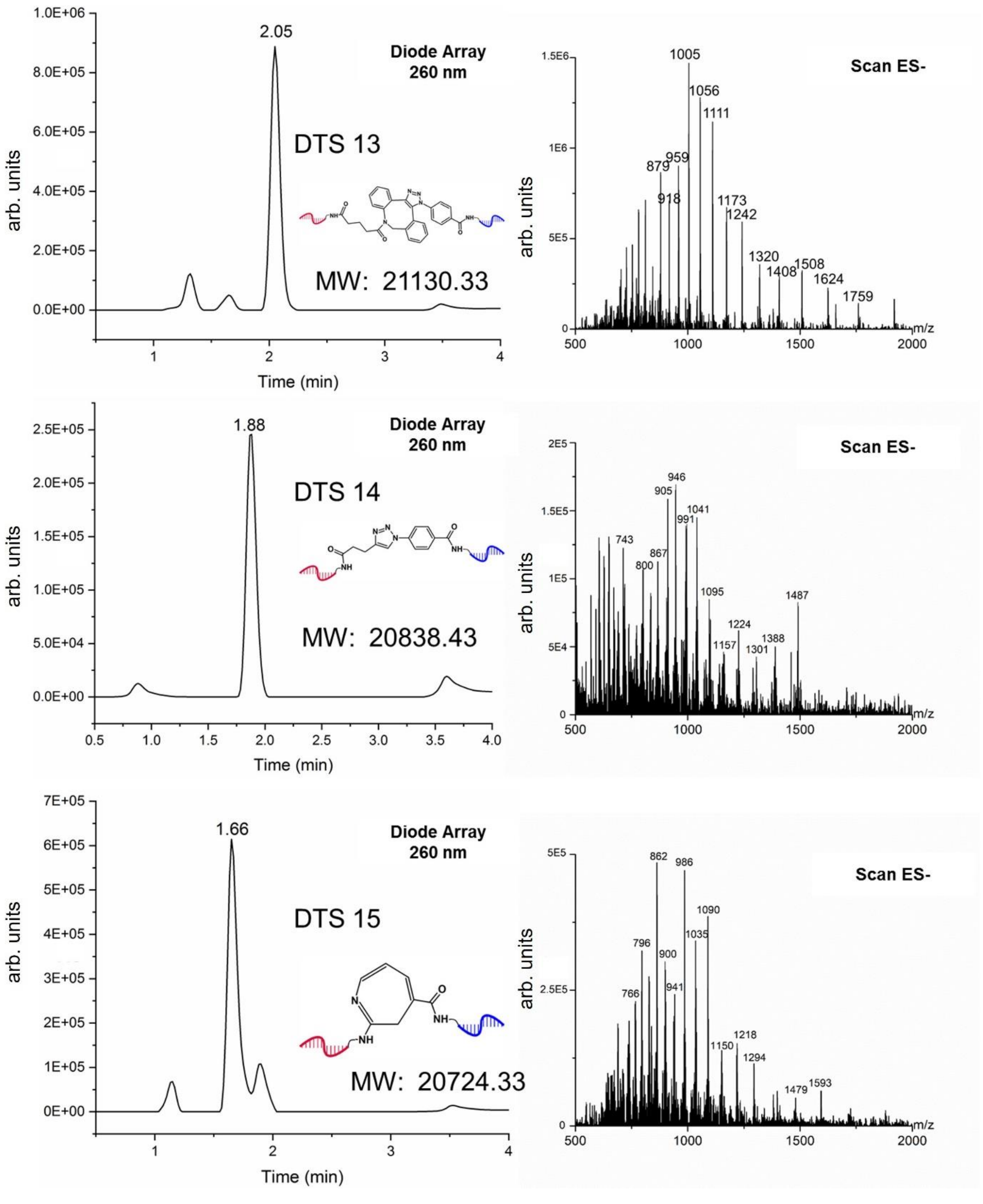

Supplementary Figure 27. UPLC chromatogram and ESI-MS validation of Sub-library members 13-15. ES: Electrospray Ionization Mode, arb.units: Arbitrary Units.

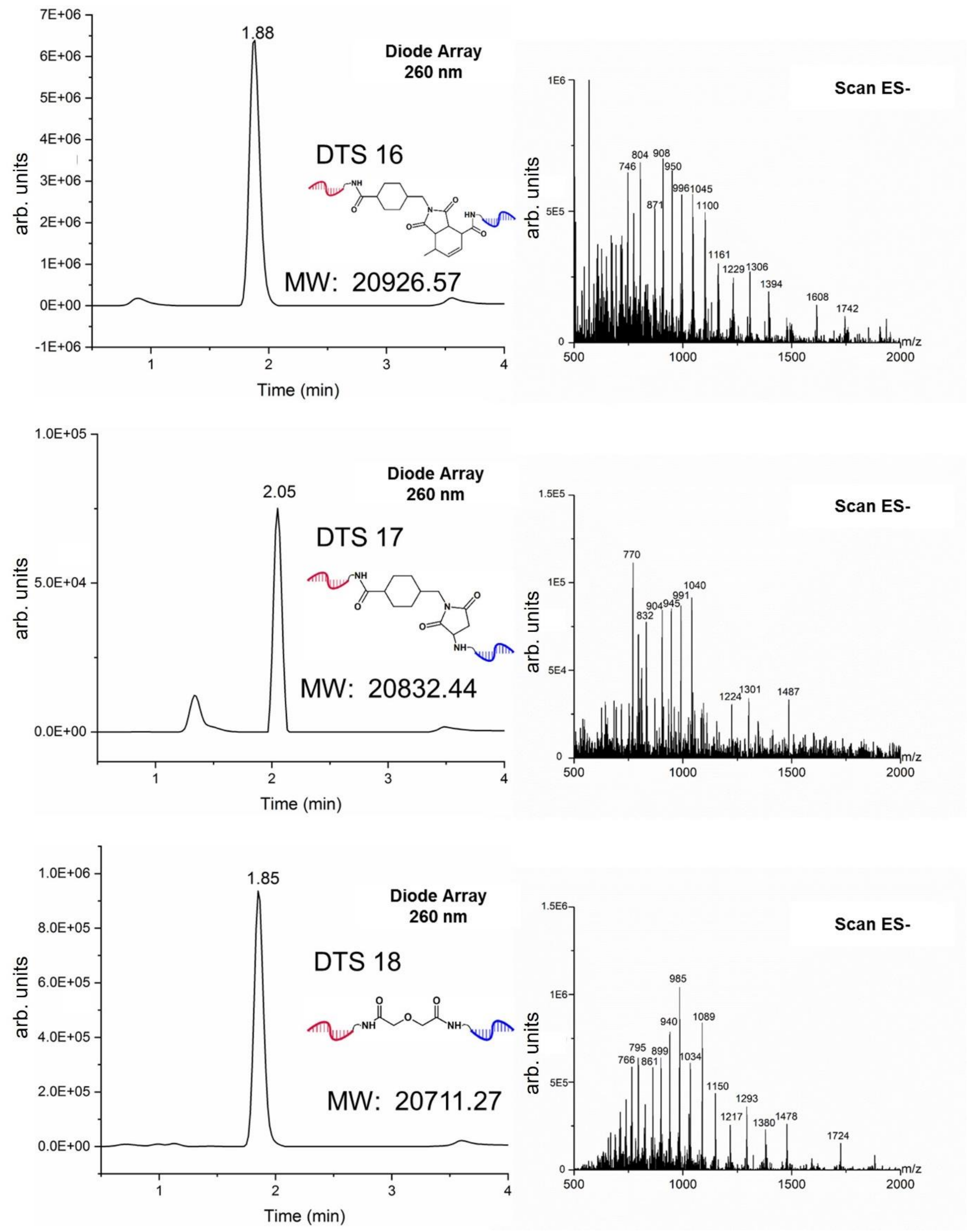

Supplementary Figure 28. UPLC chromatogram and ESI-MS validation of Sub-library members 16-18. ES: Electrospray Ionization Mode, arb.units: Arbitrary Units.

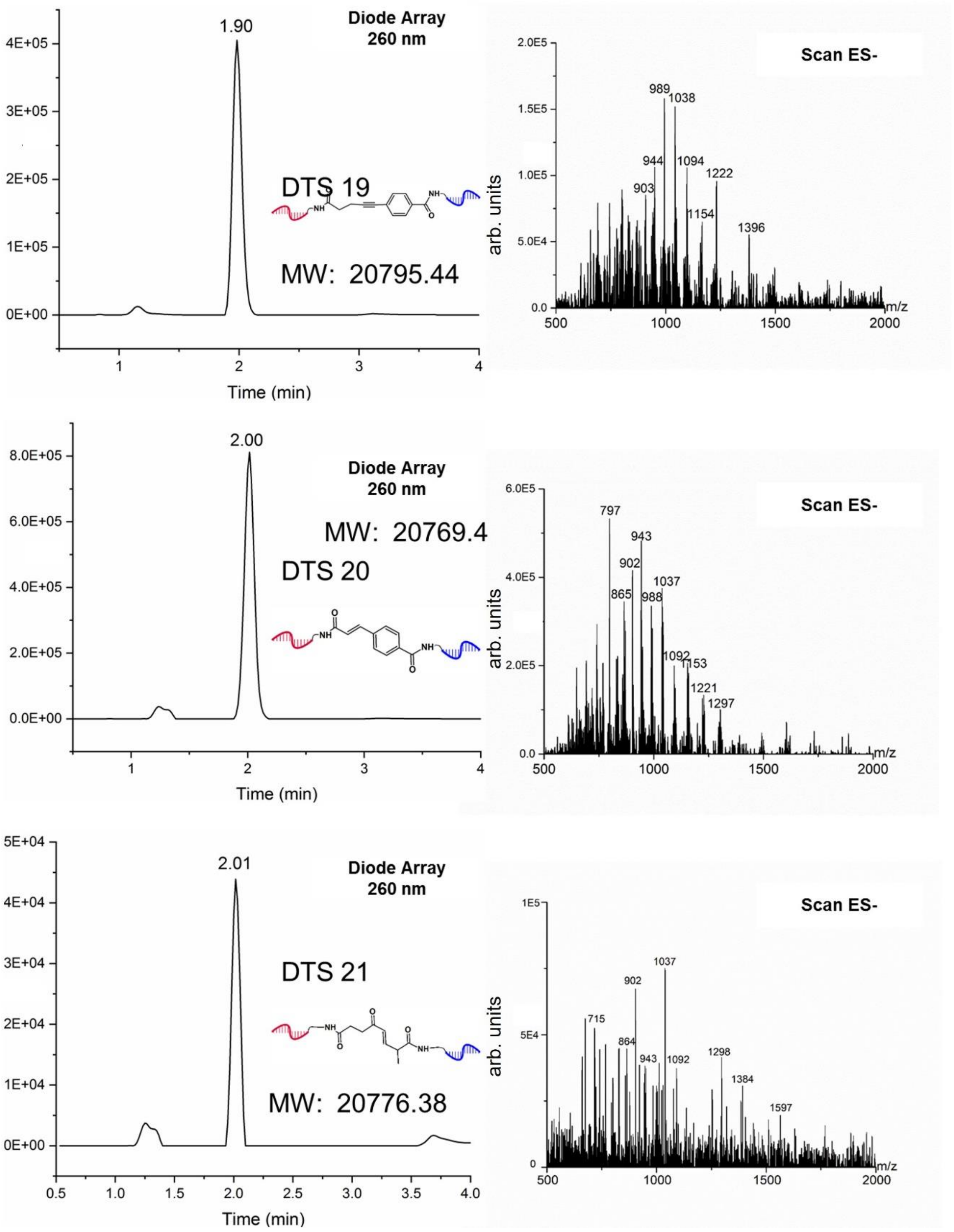

Supplementary Figure 29. UPLC chromatogram and ESI-MS validation of Sub-library members 19-21. ES: Electrospray Ionization Mode, arb.units: Arbitrary Units.

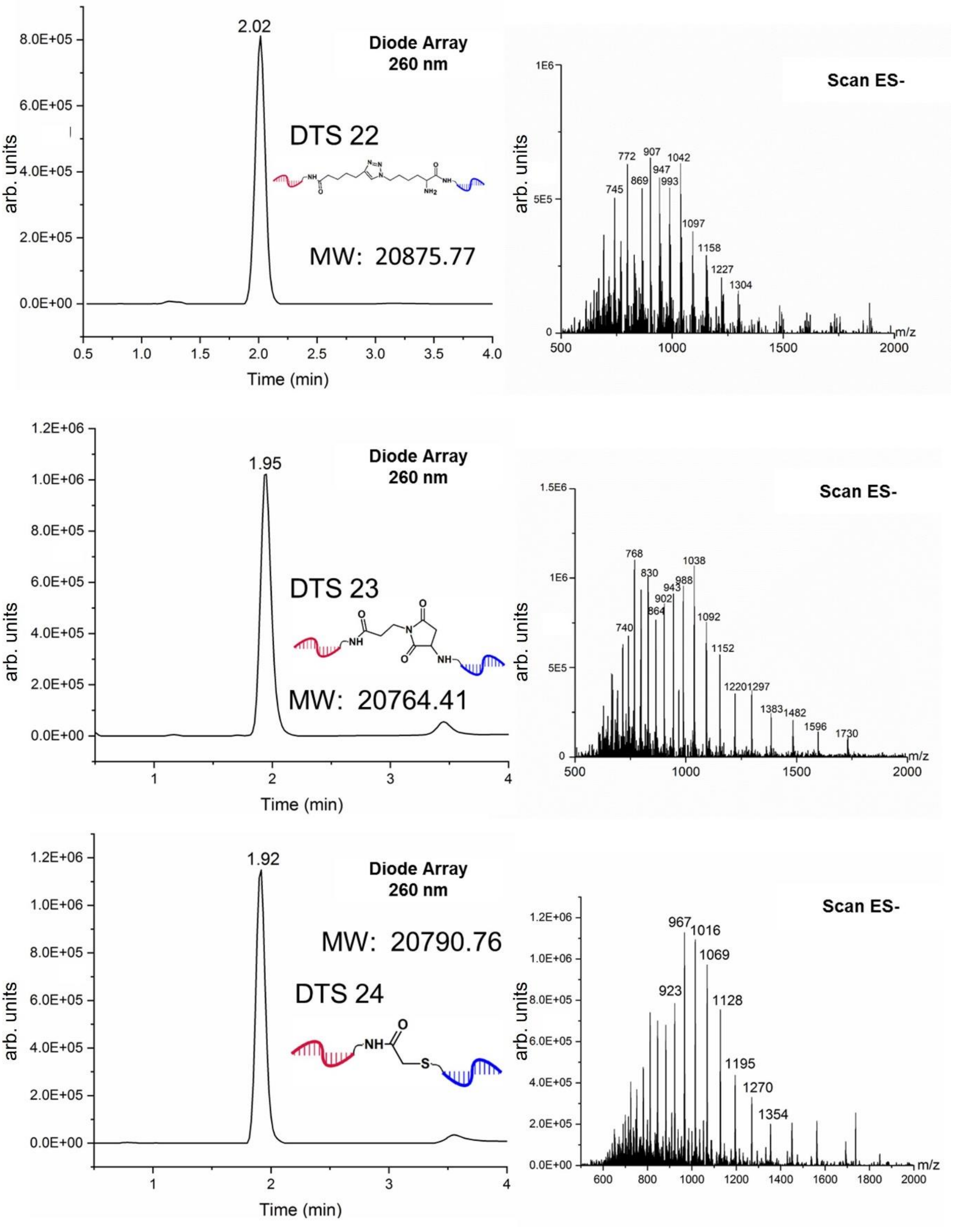

Supplementary Figure 30. UPLC chromatogram and ESI-MS validation of Sub-library members 22-24. ES: Electrospray Ionization Mode, arb.units: Arbitrary Units.

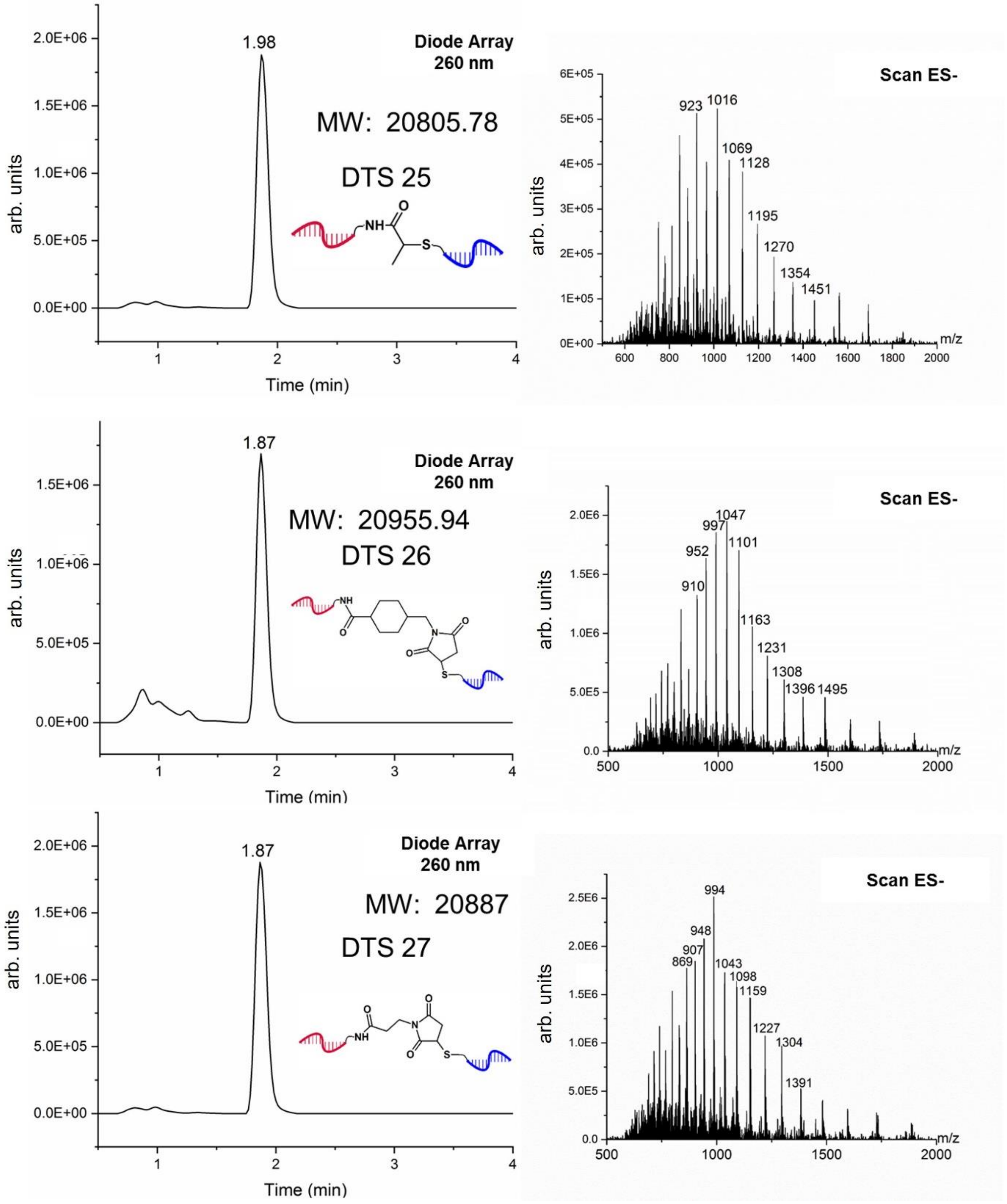

Supplementary Figure 31. UPLC chromatogram and ESI-MS validation of Sub-library members 25-27. ES: Electrospray Ionization Mode, arb.units: Arbitrary Units.

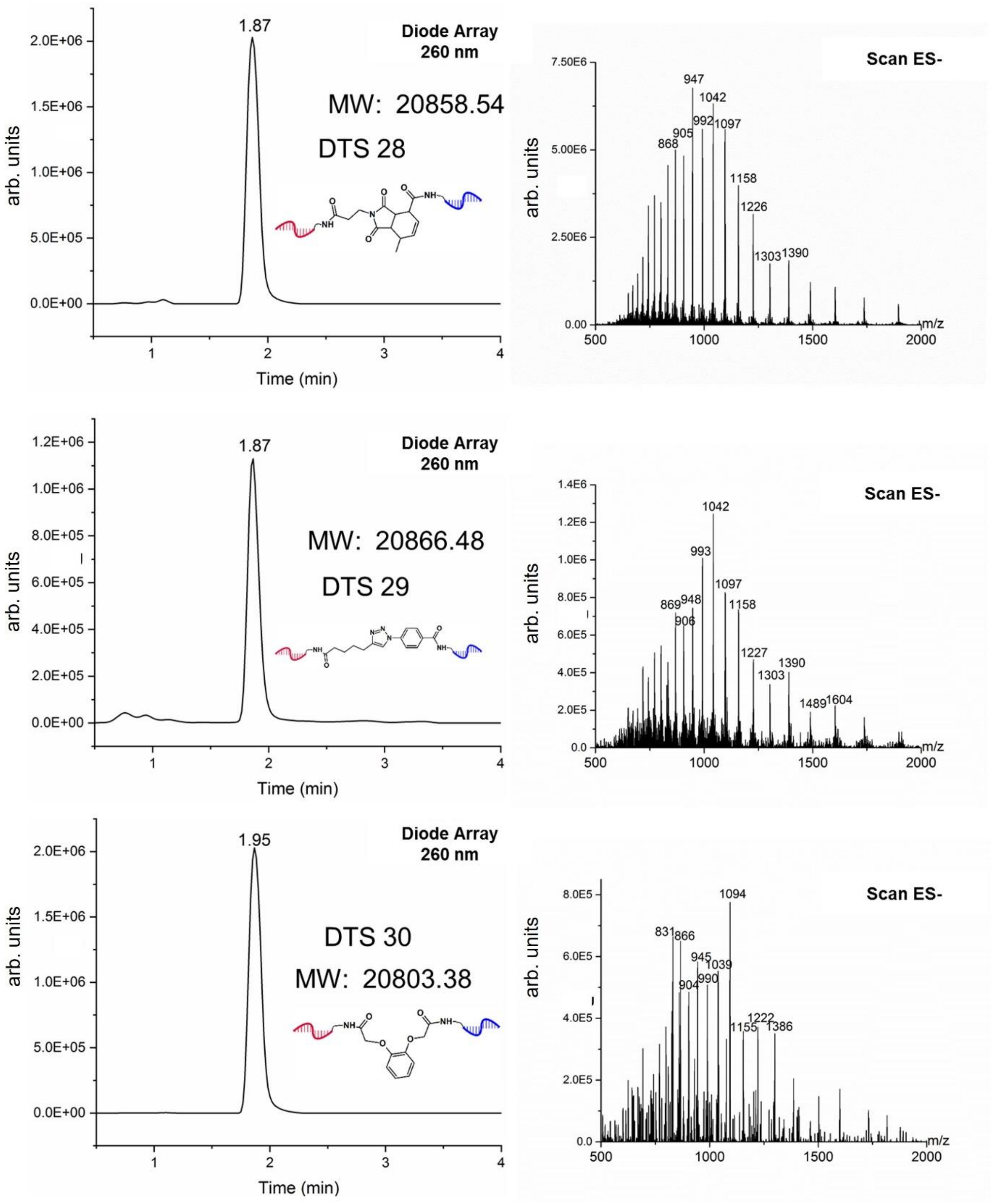

Supplementary Figure 32. UPLC chromatogram and ESI-MS validation of Sub-library members 28-30. arb.units: Arbitrary Units.

4- carboxybenzene sulfonamide –DNA conjugate

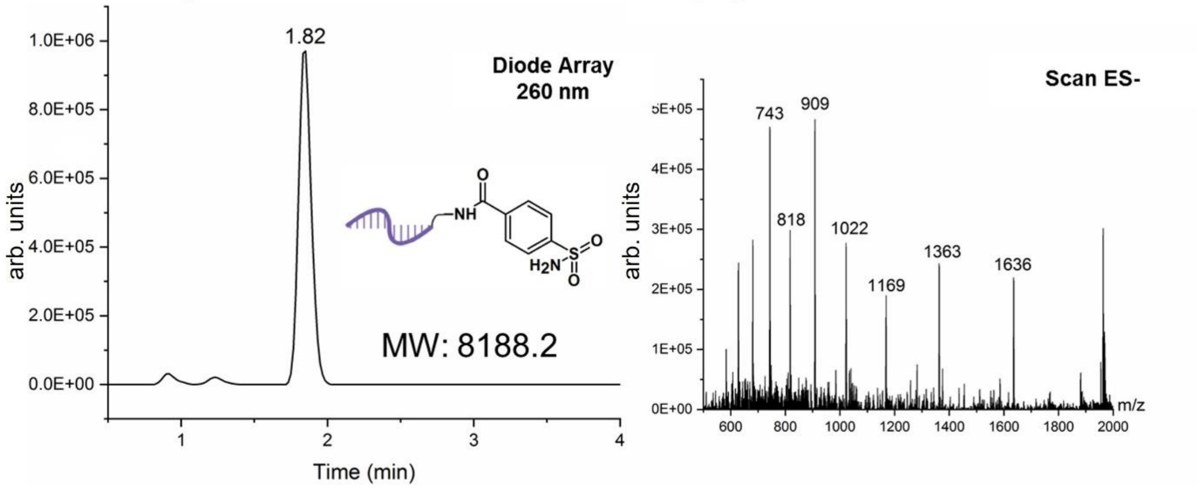

Compound A–DNA conjugate

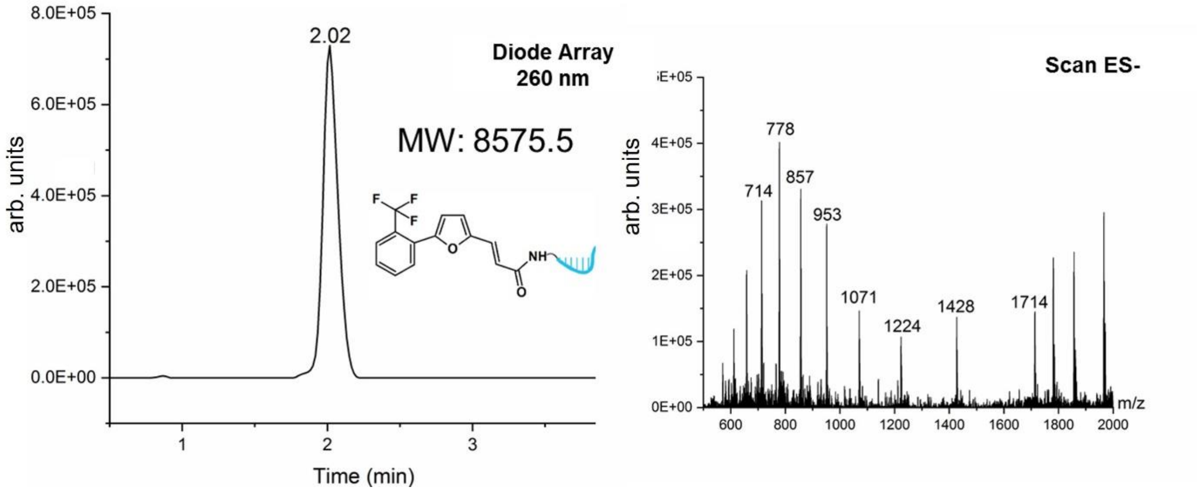

Supplementary Figure 33. UPLC chromatogram and ESI-MS validation of sub-library A (upper) and sub-library C (lower) for affinity maturation of carbonic anhydrase II. arb.units:Arbitrary units.

4-aminomethyl benzamidine–DNA conjugate

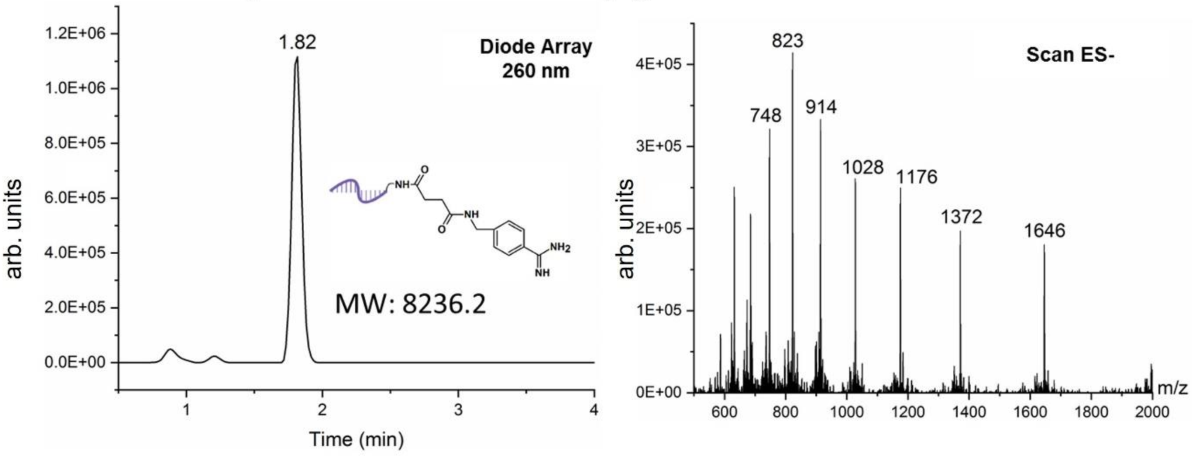

Compound B –DNA conjugate

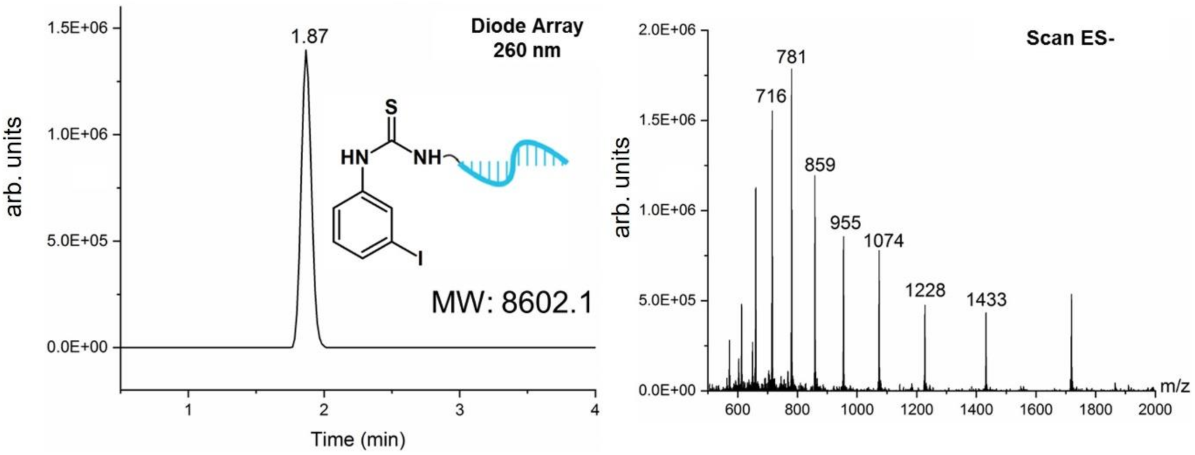

Supplementary Figure 34. UPLC chromatogram and ESI-MS validation of sub-library A (upper) and sub-library C (lower) for affinity maturation of bovine trypsin. ES: Electrospray Ionization Mode, AU: Arbitrary Units.

Supplementary Note 2

Characterization of off-DNA compounds by UPLC-ESI-MS

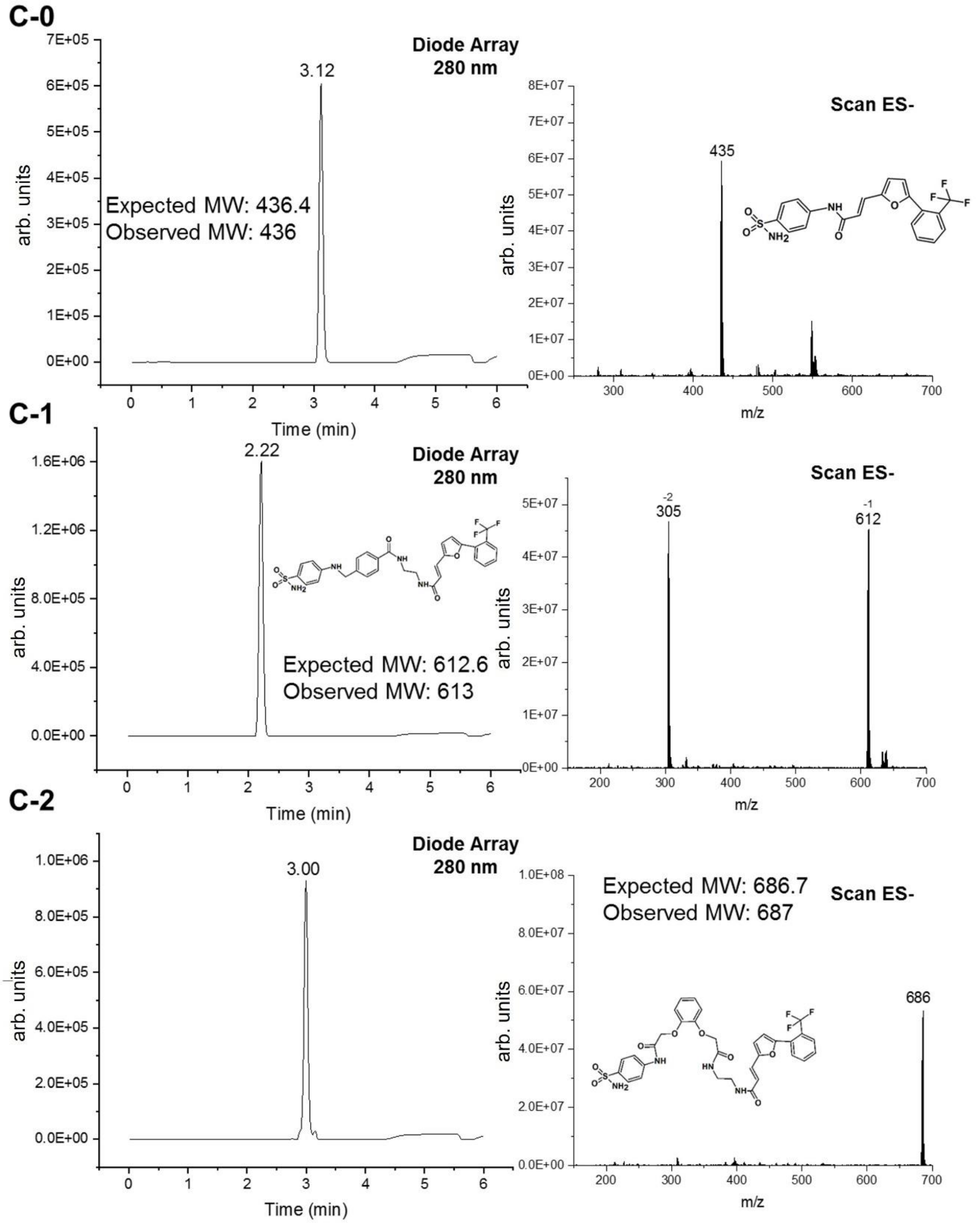

Supplementary Figure 35. UPLC and ESI-MS validation of compounds C-0, C-1, and C-2. ES: Electrospray Ionization Mode, arb.units: Arbitrary Units.

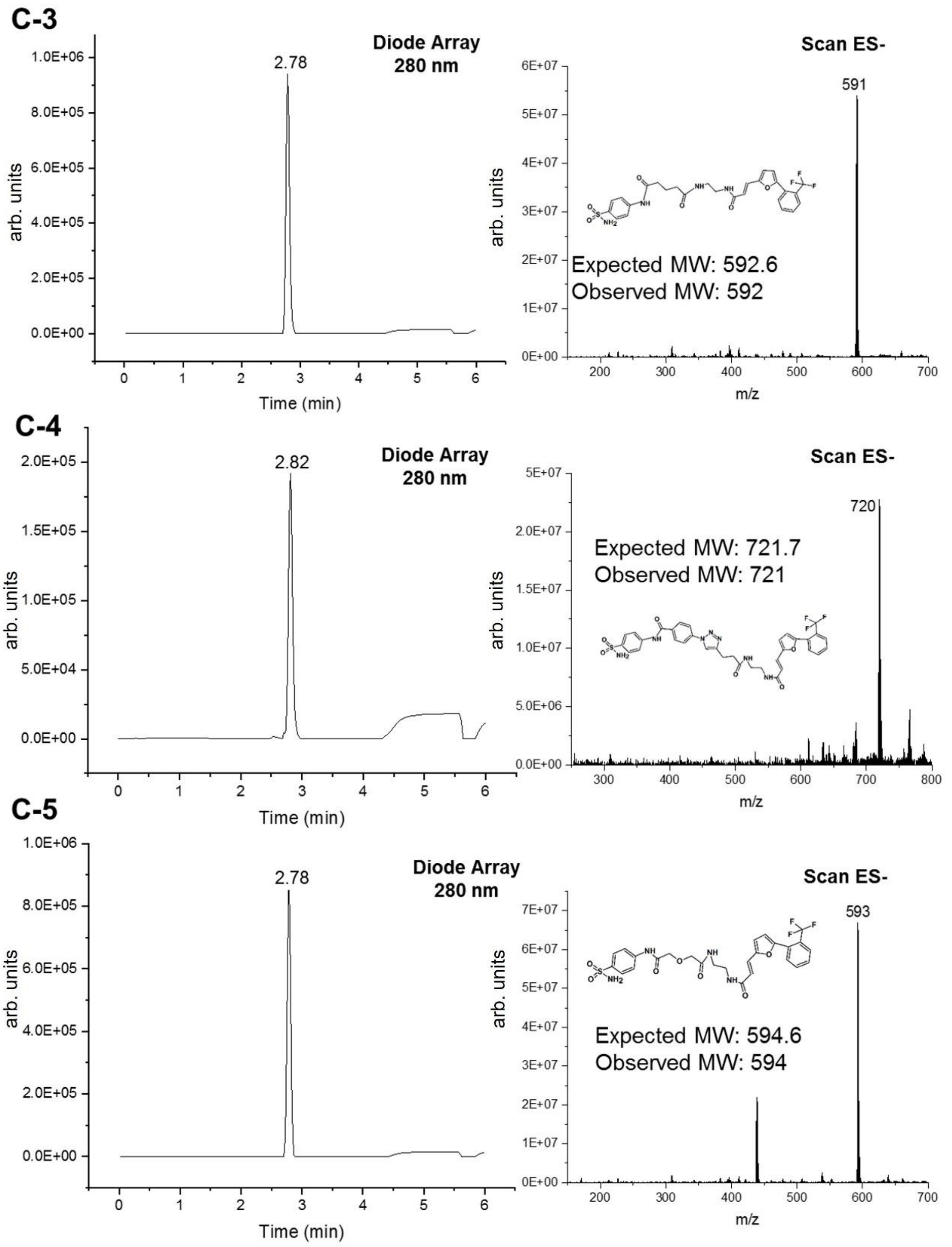

Supplementary Figure 36. UPLC and ESI-MS validation of compounds C-3, C-4, and C-5. ES: Electrospray Ionization Mode, arb. units: Arbitrary Units.

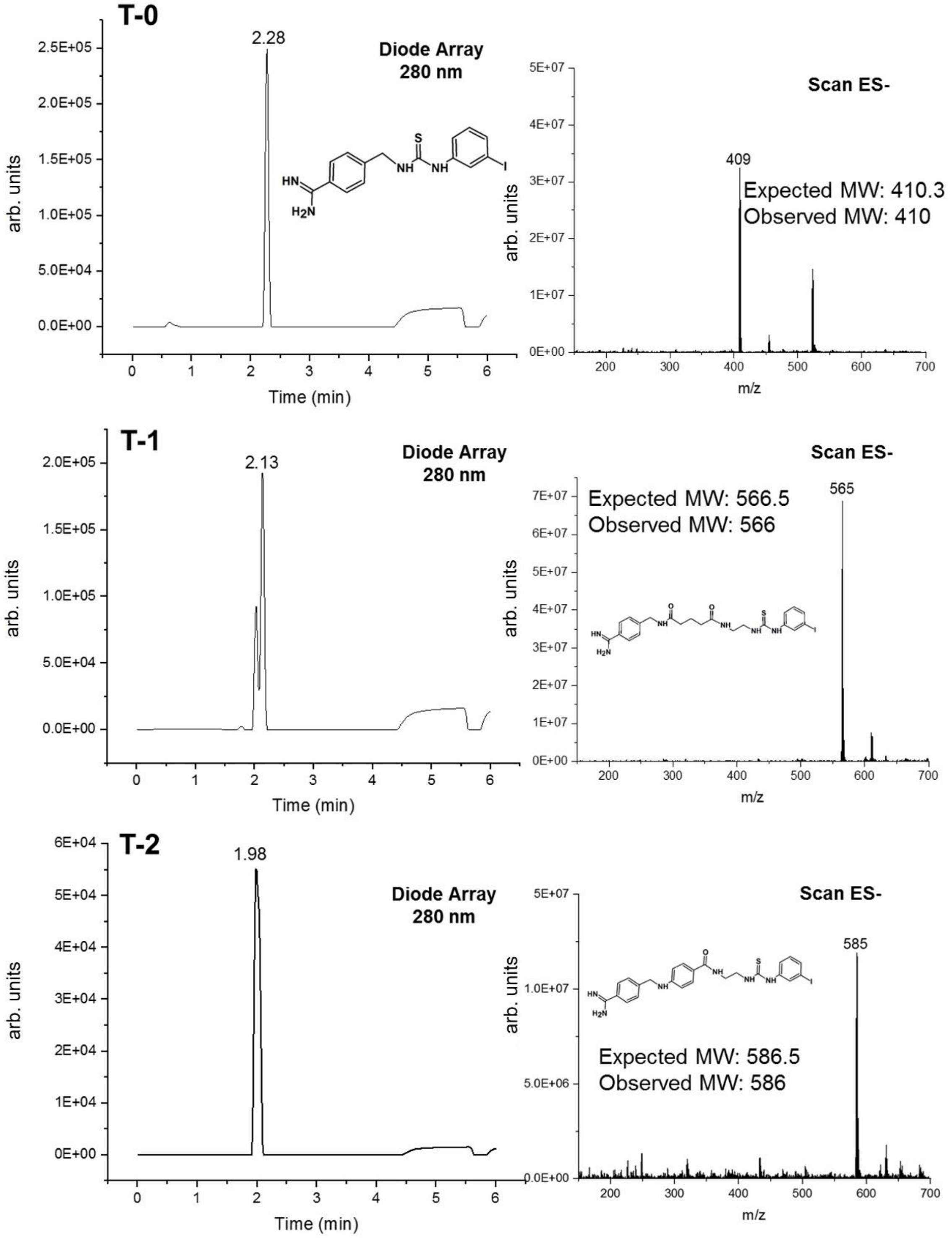

Supplementary Figure 37. UPLC and ESI-MS validation of compounds T-0, T-1, and T-2. ES: Electrospray Ionization Mode, arb. units: Arbitrary Units.

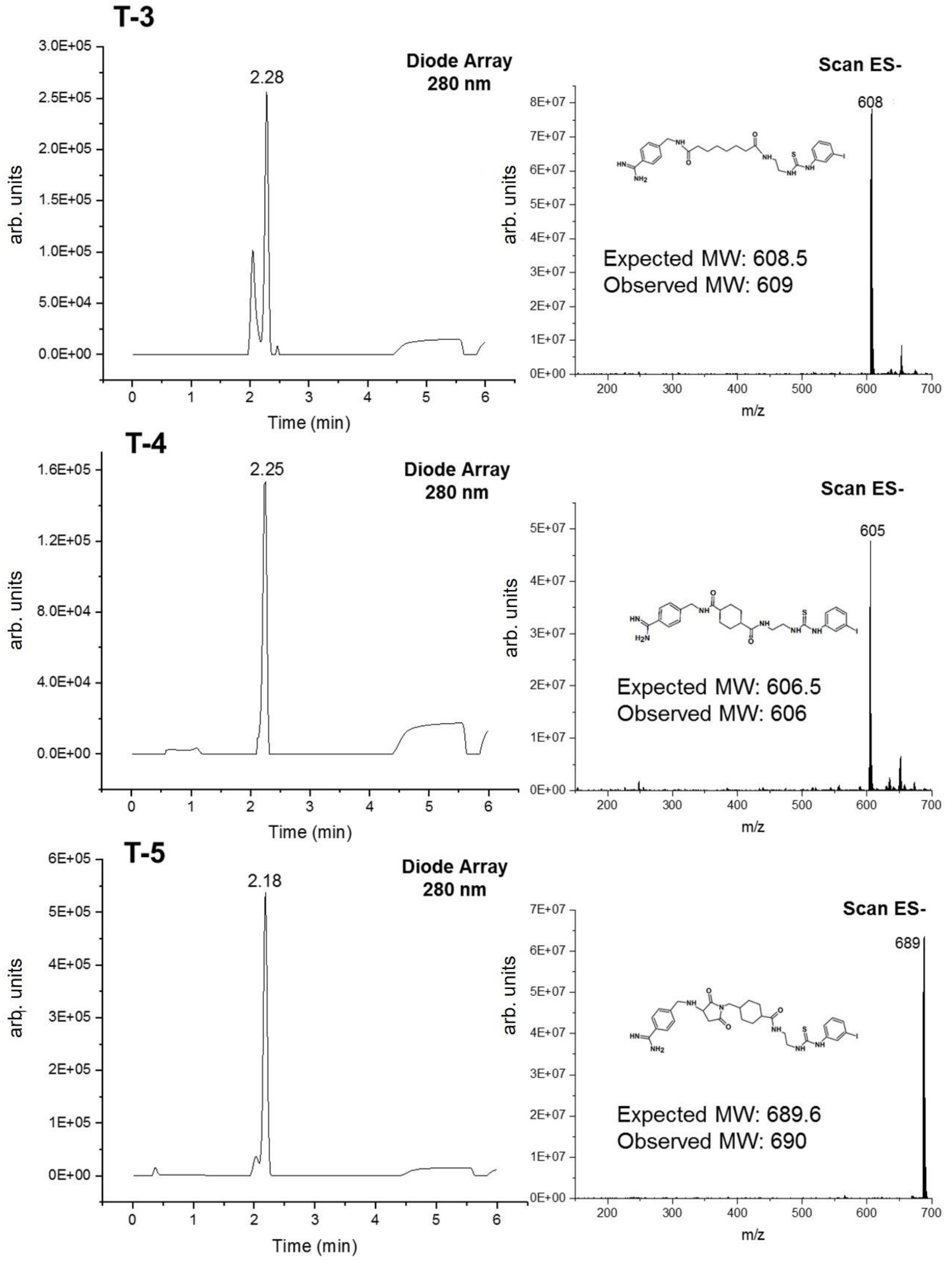

Supplementary Figure 38. UPLC and ESI-MS validation of compounds T-3, T-4, and T-5. ES: Electrospray Ionization Mode, arbitrary units: Arbitrary Units.

693_12_828

Expected MW: 606.67272
Observed MW: 606

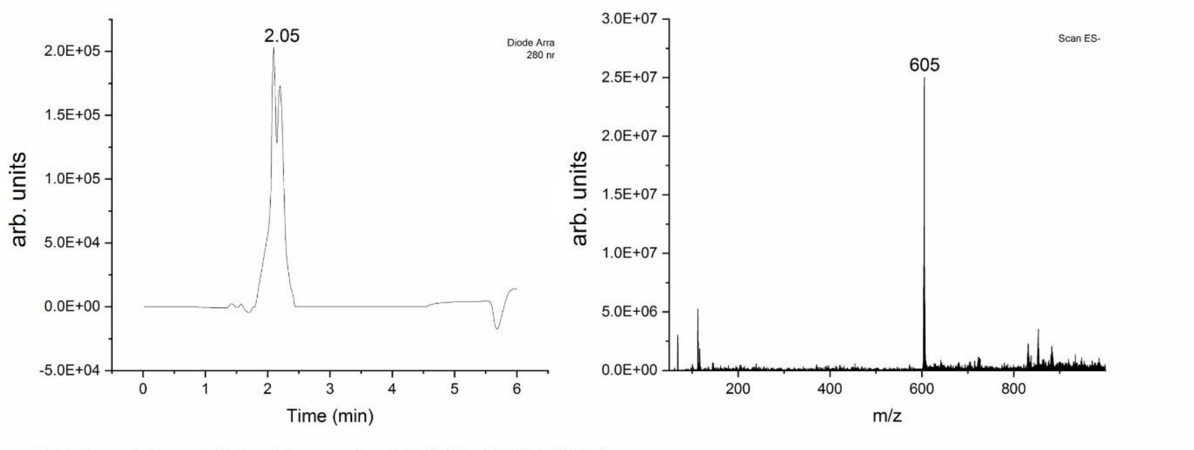

693_12_826

Expected MW: 660.7234
Observed MW: 660

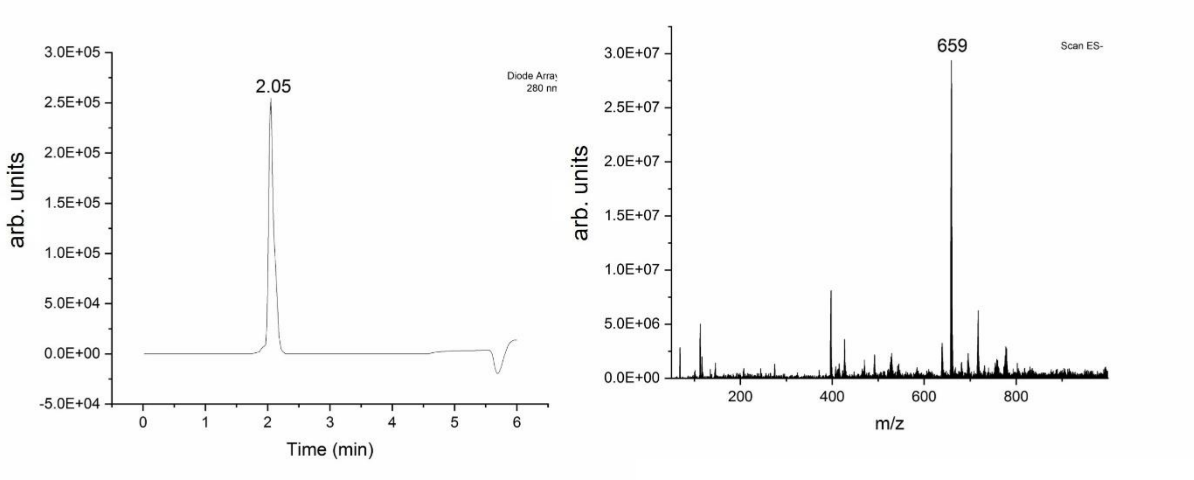

693_12_787

Expected MW: 665.74322
Observed MW: 665

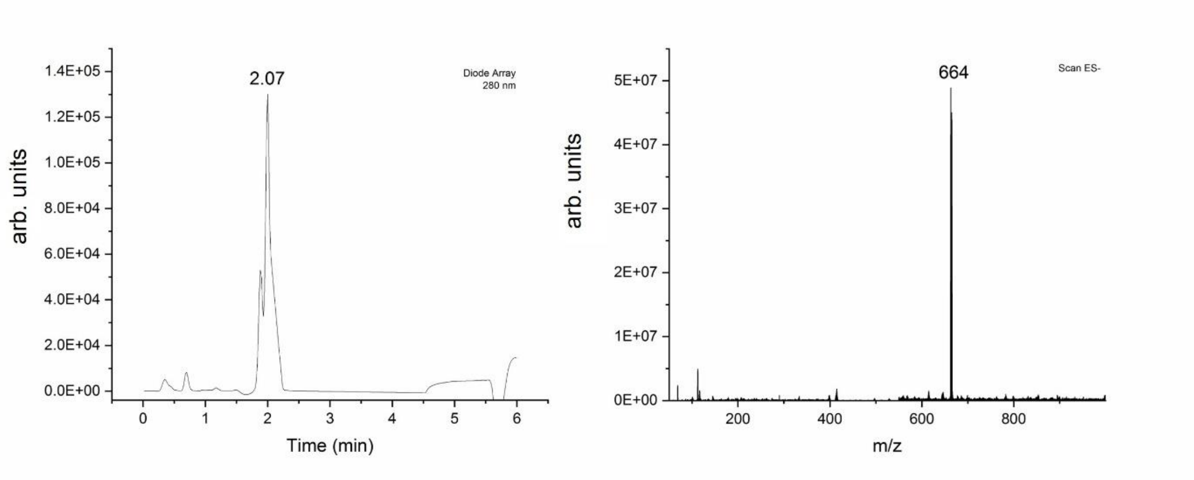

Supplementary Figure 39. UPLC and ESI-MS validation of compounds **693_12_828**, **693_12_826**, and **693_12_787**. ES: Electrospray Ionization Mode, arb units: Arbitrary Units.

693_24_828

Expected MW: 556.63718
Observed MW: 556

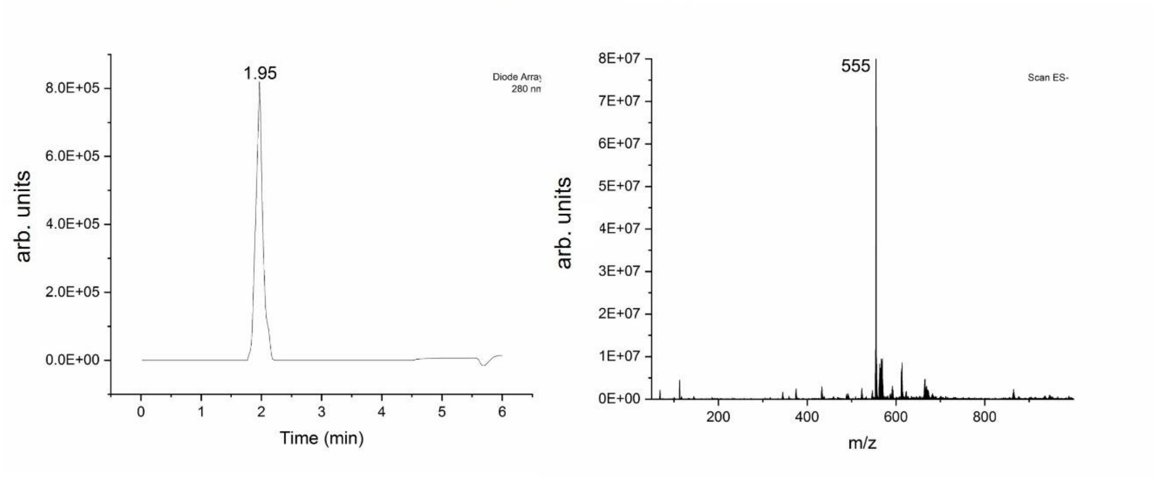

693_24_826

Expected MW: 610.68786
Observed MW: 610

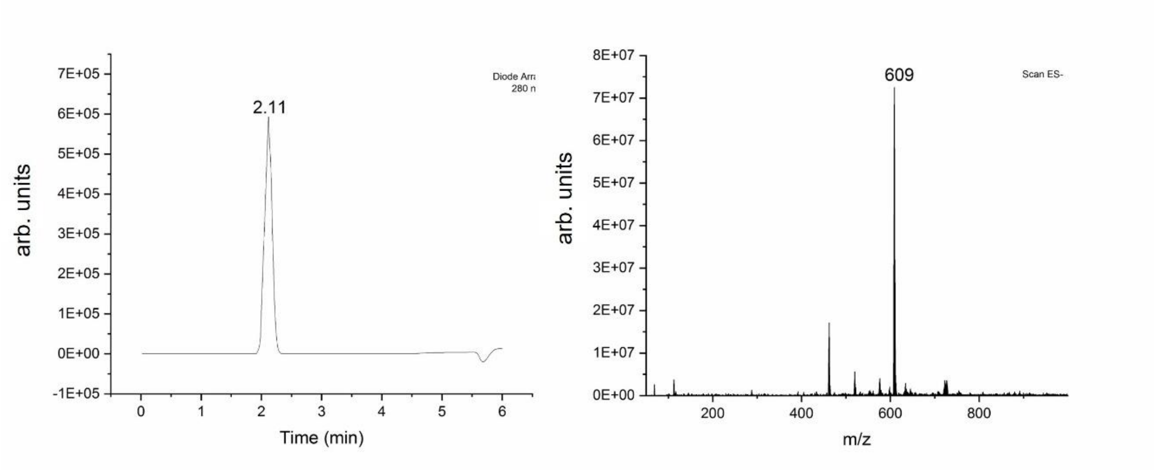

693_24_787

Expected MW: 473.55188
Observed MW: 473

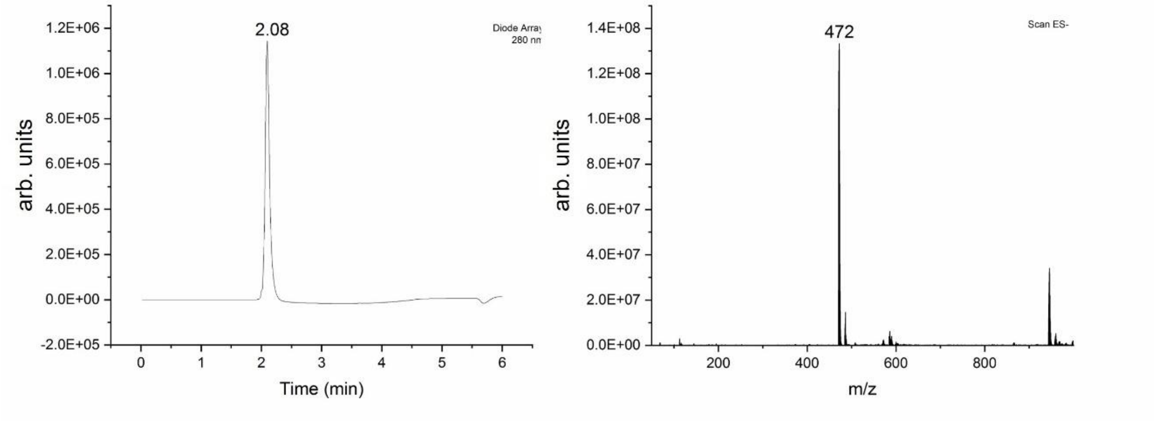

Supplementary Figure 40. UPLC and ESI-MS validation of compounds **693_24_828**, **693_24_826**, and **693_24_787**. ES: Electrospray Ionization Mode, arb.units: Arbitrary Units.

693_10_828

Expected MW: 527.57282
Observed MW: 527

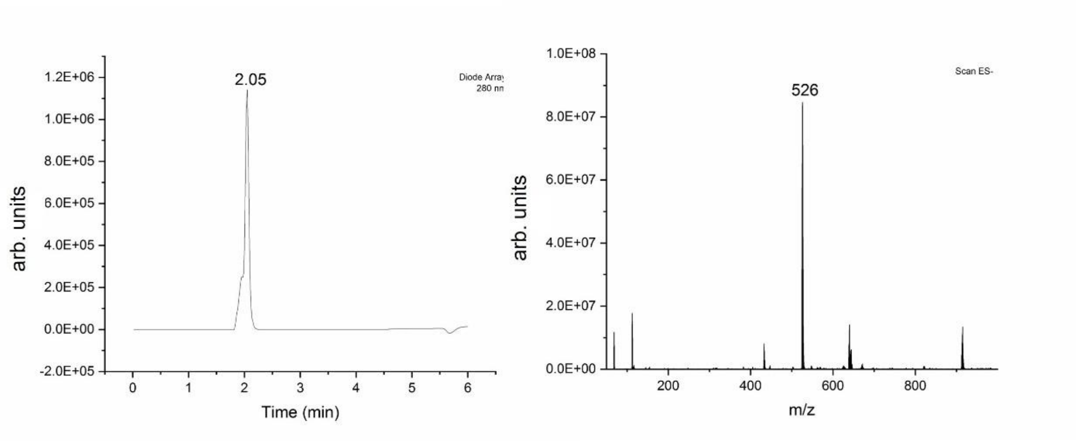

693_10_826

Expected MW: 581.6235
Observed MW: 581

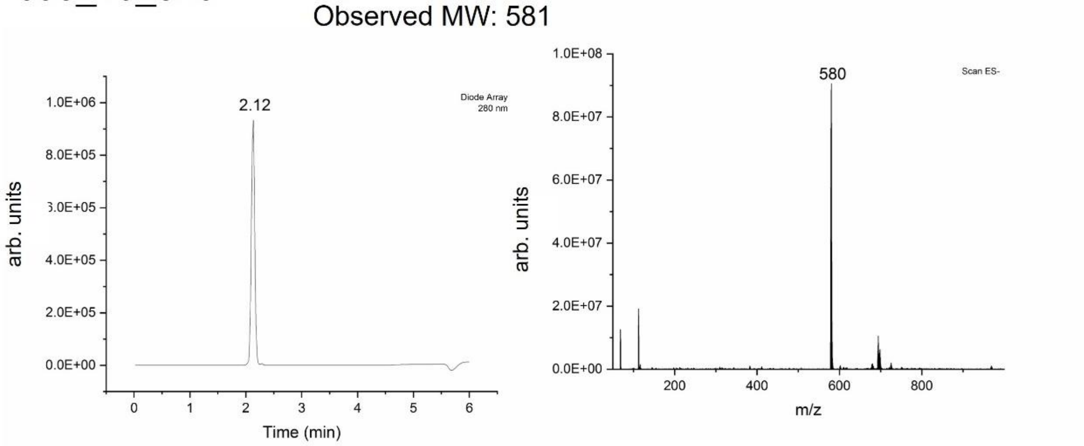

693_10_787

Expected MW: 586.64332
Observed MW: 586

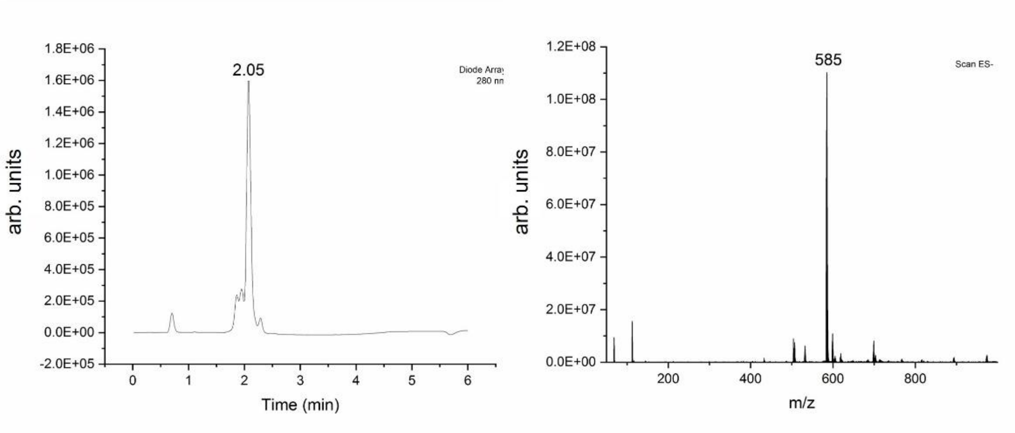

Supplementary Figure 41. UPLC and ESI-MS validation of compounds **693_10_828**, **693_10_826**, and **693_10_787**. ES: Electrospray Ionization Mode, arb. units: Arbitrary Units.

693_4_828

Expected MW: 523.62718
Observed MW: 523

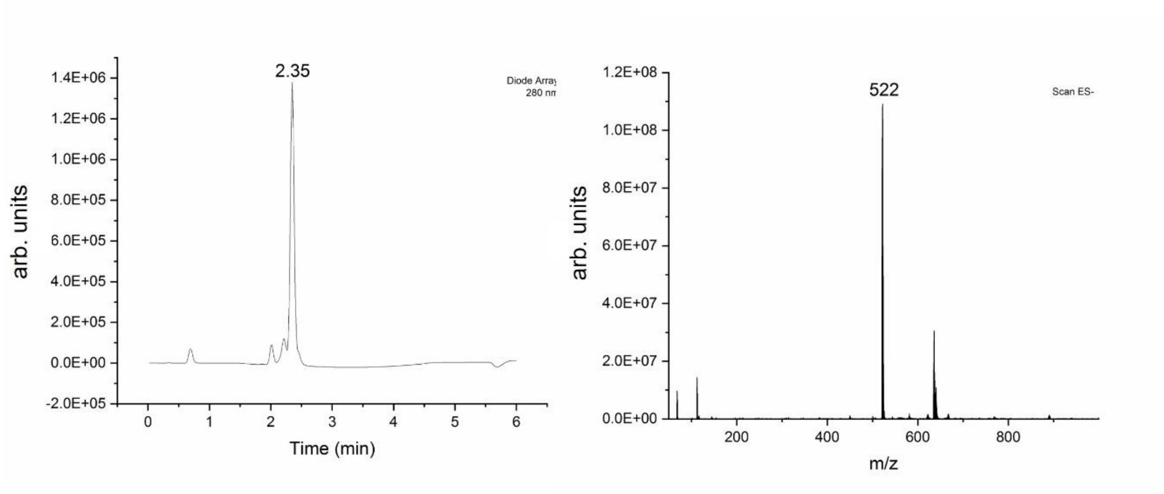

693_4_826

Expected MW: 577.67786
Observed MW: 577

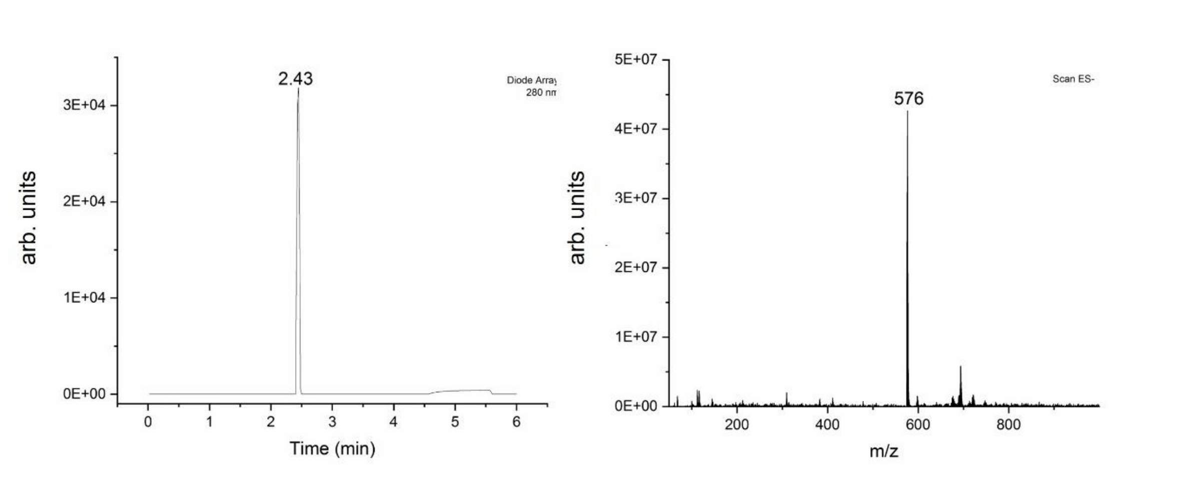

693_4_787

Expected MW: 582.69768
Observed MW: 582

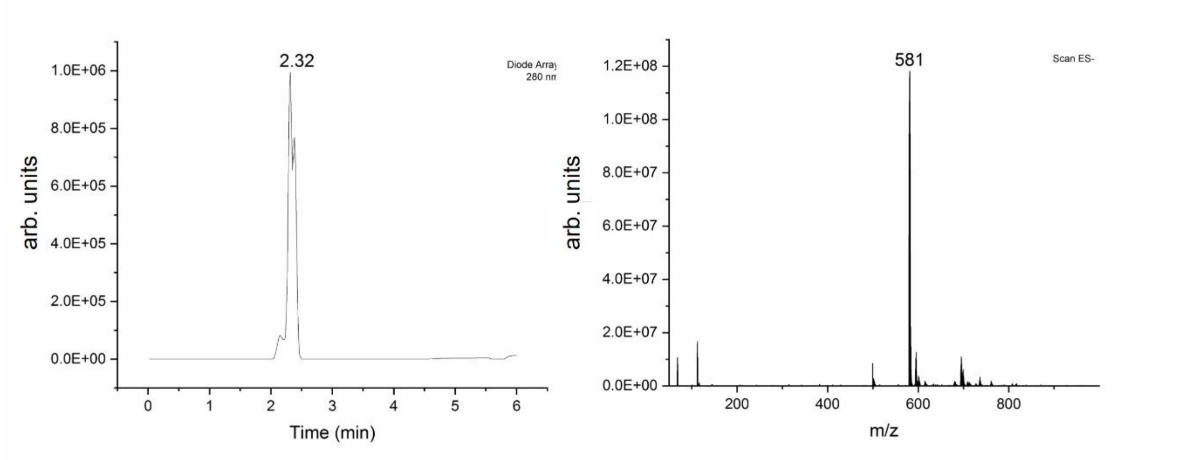

Supplementary Figure 42. UPLC and ESI-MS validation of compounds **693_4_828**, **693_4_826**, and **693_4_787**. ES: Electrospray Ionization Mode, arb. units: Arbitrary Units.

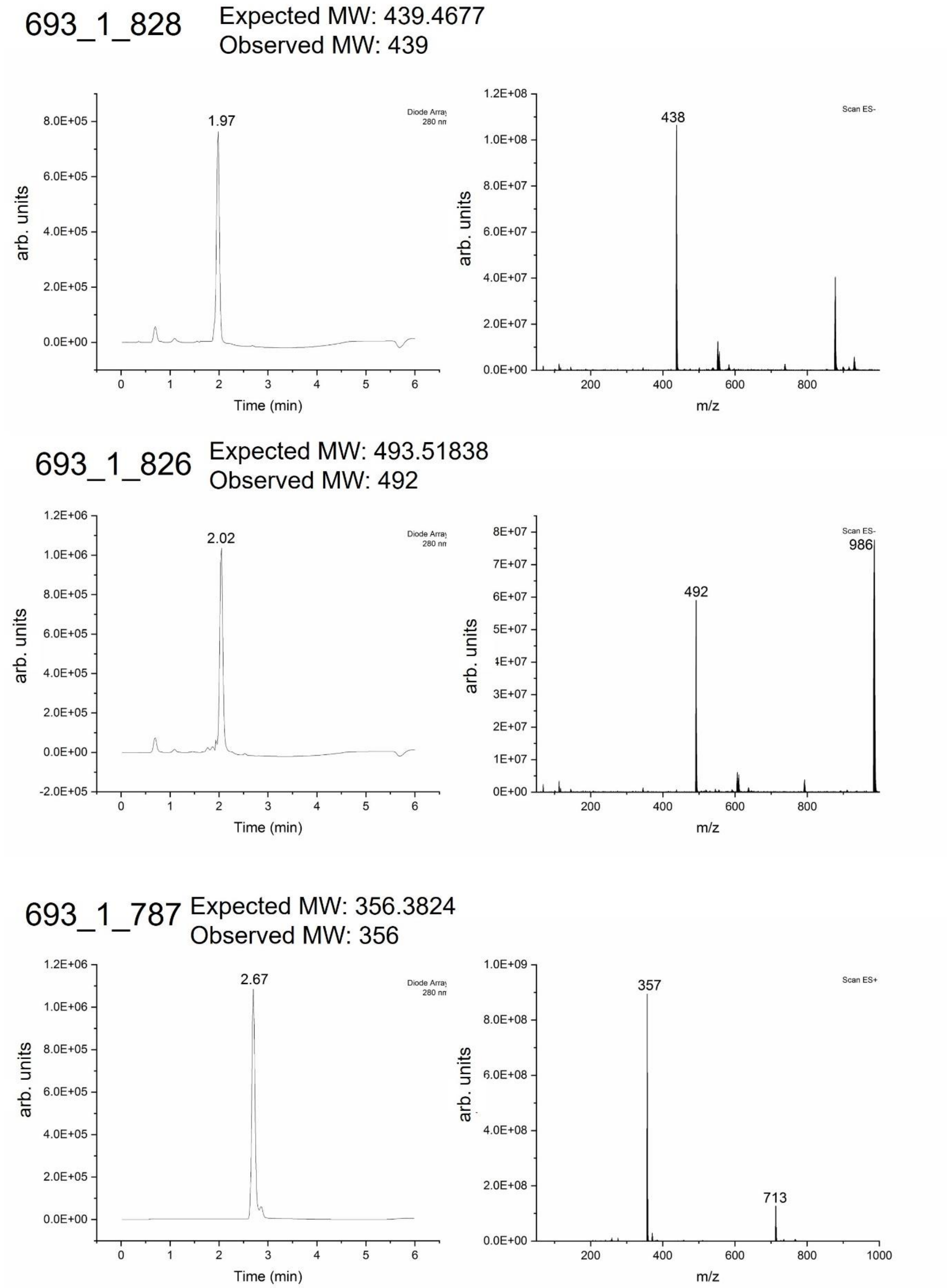

Supplementary Figure 43. UPLC and ESI-MS validation of compounds **693_1_828**, **693_1_826**, and **693_1_787**. ES: Electrospray Ionization Mode, arb. units: Arbitrary Units.

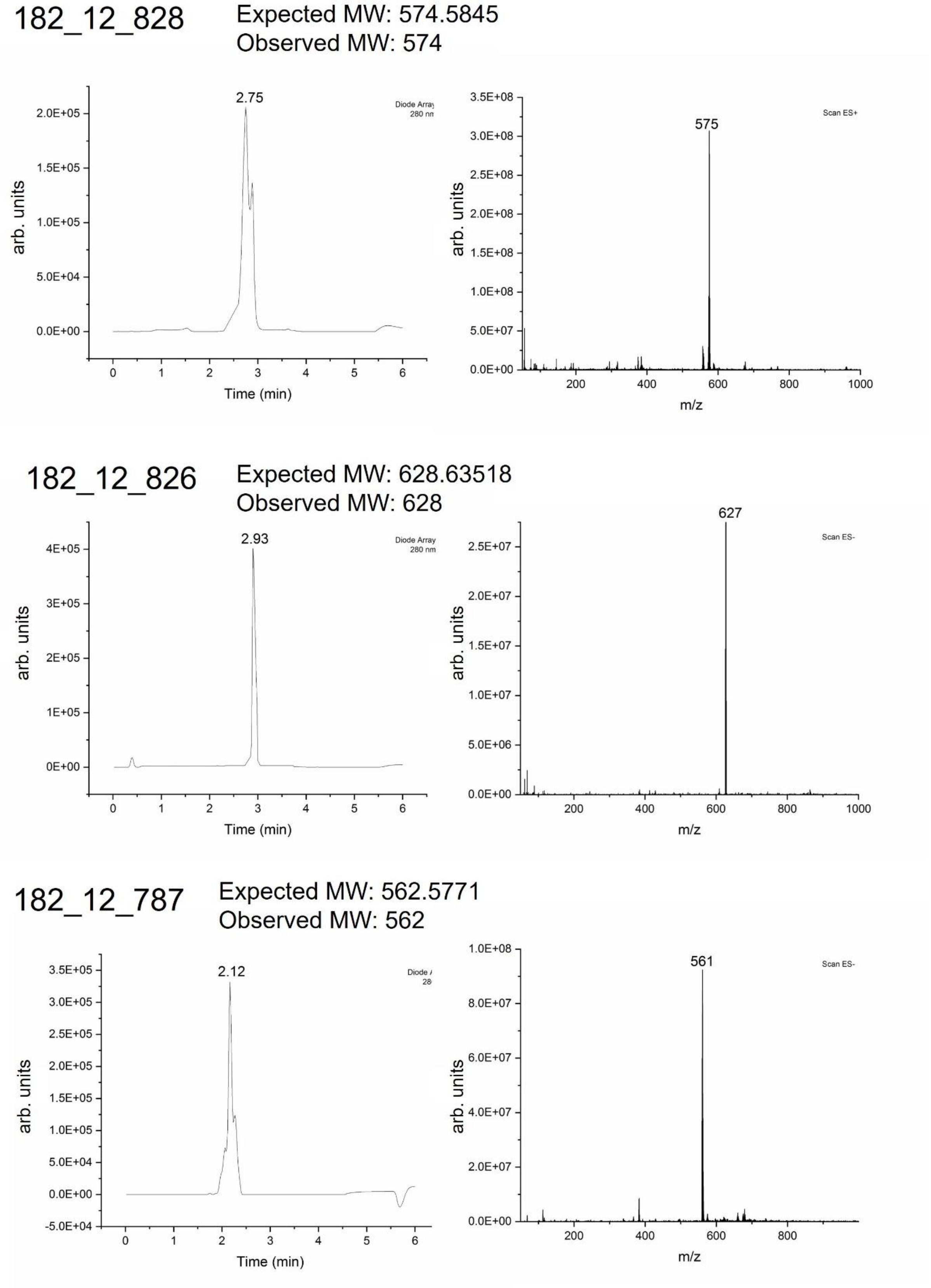

Supplementary Figure 44. UPLC and ESI-MS validation of compounds **182_12_828**, **182_12_826**, and **182_12_787**. ES: Electrospray Ionization Mode, arb. units: Arbitrary Units.

182_24_828 Expected MW: 524.54896
Observed MW: 524

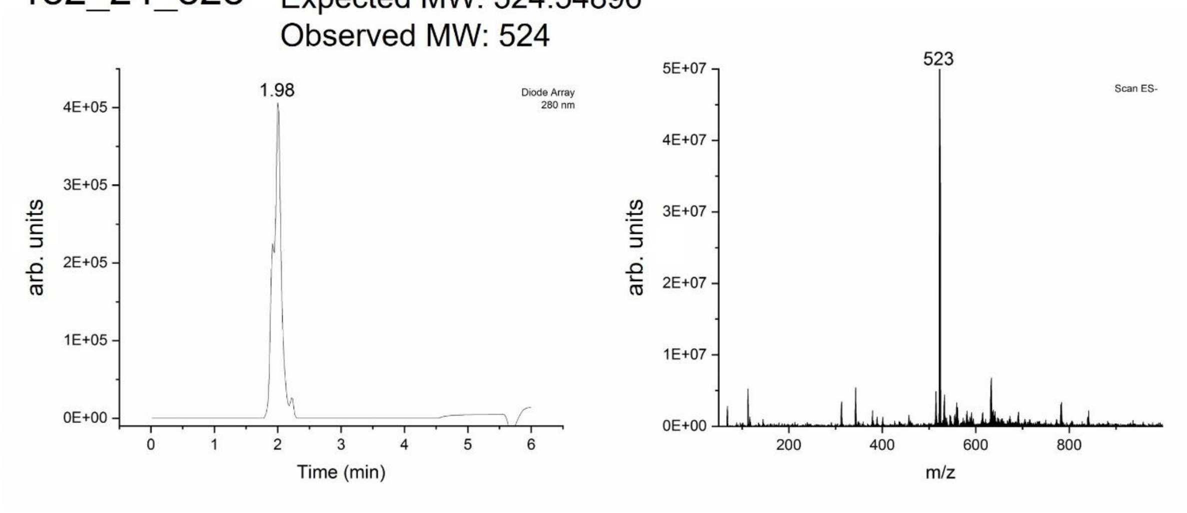

182_24_826 Expected MW: 578.59964
Observed MW: 578

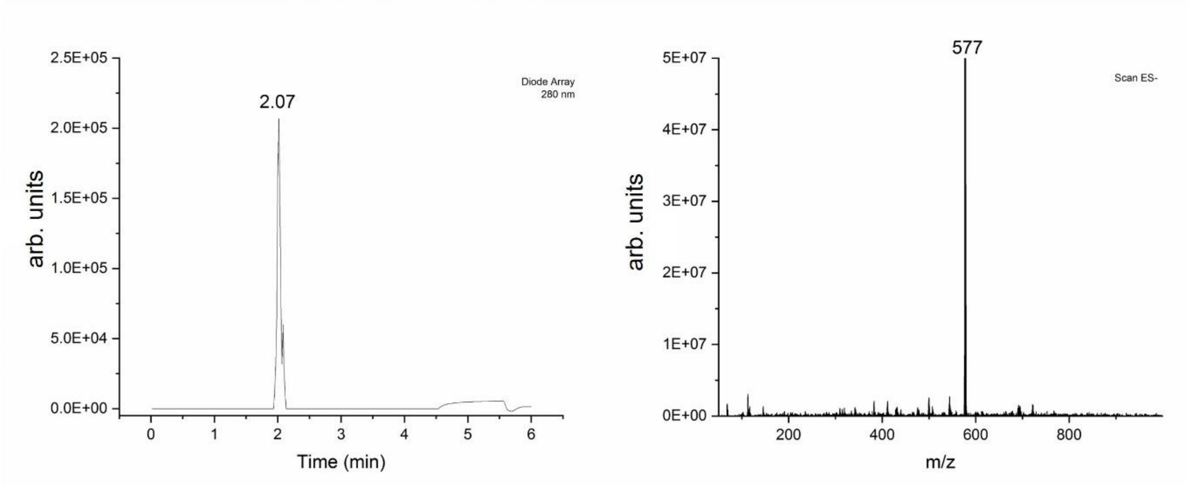

182_24_787 Expected MW: 441.46366
Observed MW:441

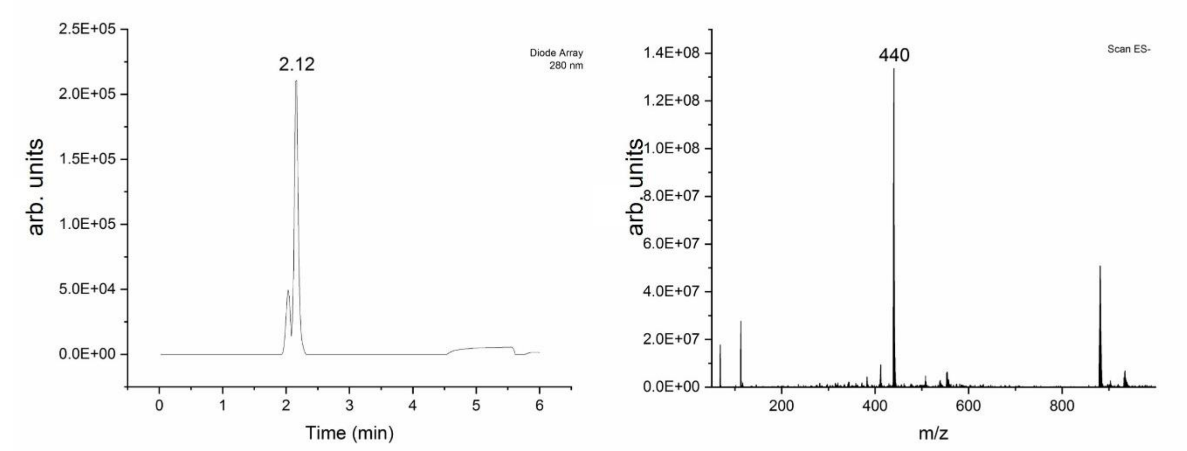

Supplementary Figure 45. UPLC and ESI-MS validation of compounds **182_24_828**, **182_24_826**, and **182_24_787**. ES: Electrospray Ionization Mode, arb. units: Arbitrary Units.

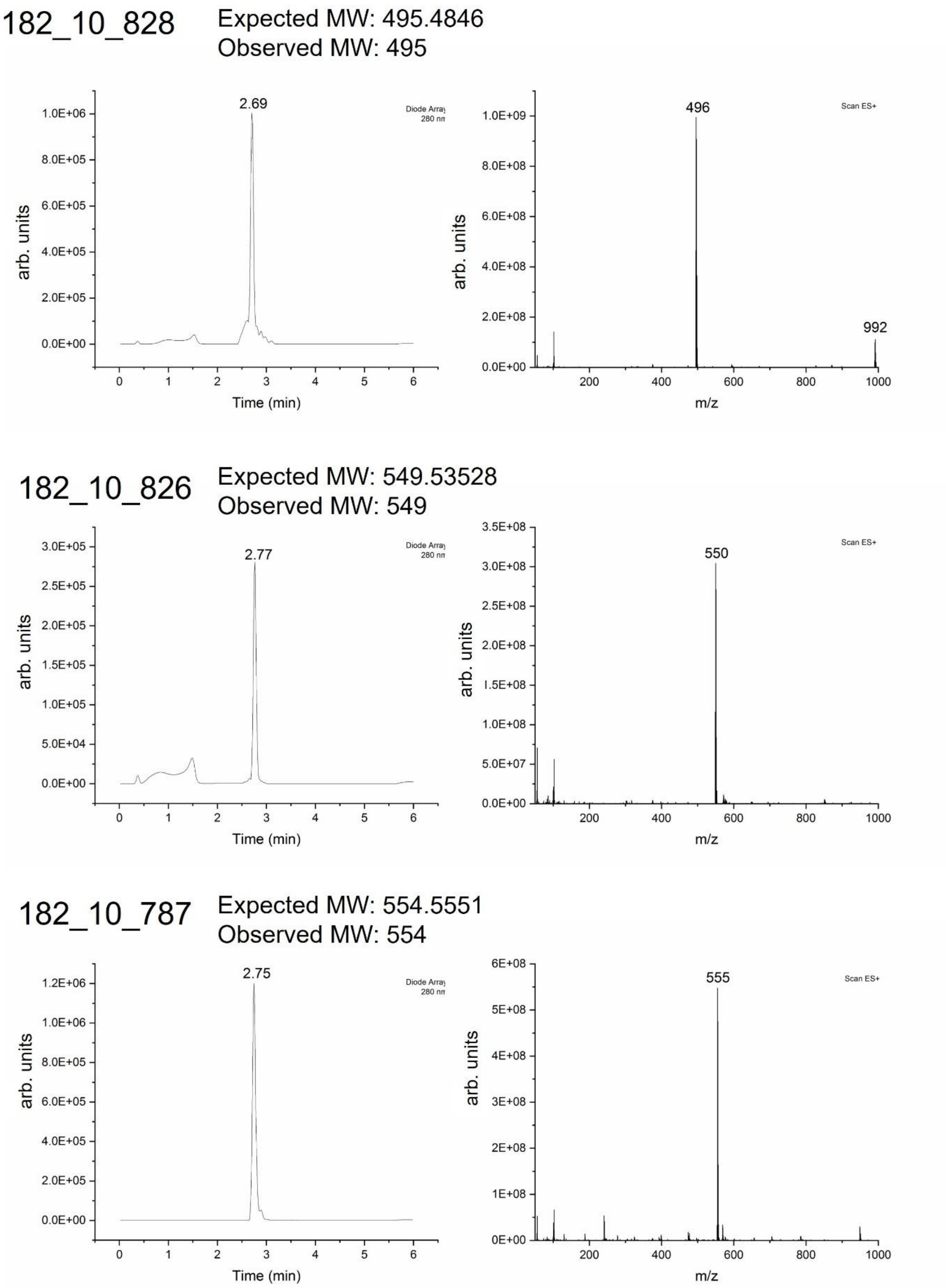

Supplementary Figure 46. UPLC and ESI-MS validation of compounds **182_10_828**, **182_10_826**, and **182_10_787**. ES: Electrospray Ionization Mode, arb. units: Arbitrary Units.

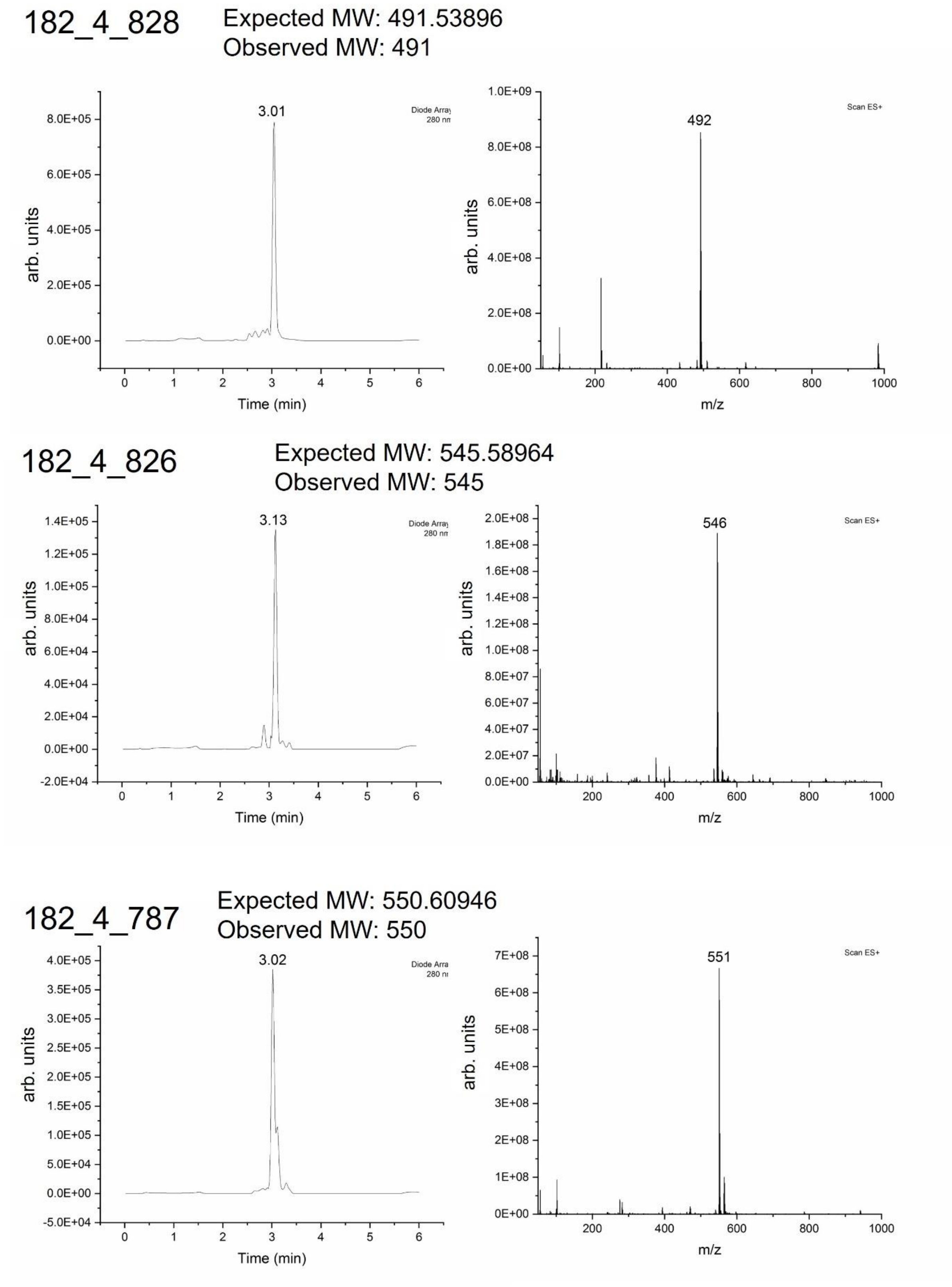

Supplementary Figure 47. UPLC and ESI-MS validation of compounds **182_4_828**, **182_4_826**, and **182_4_787**. ES: Electrospray Ionization Mode, arb. units: Arbitrary Units.

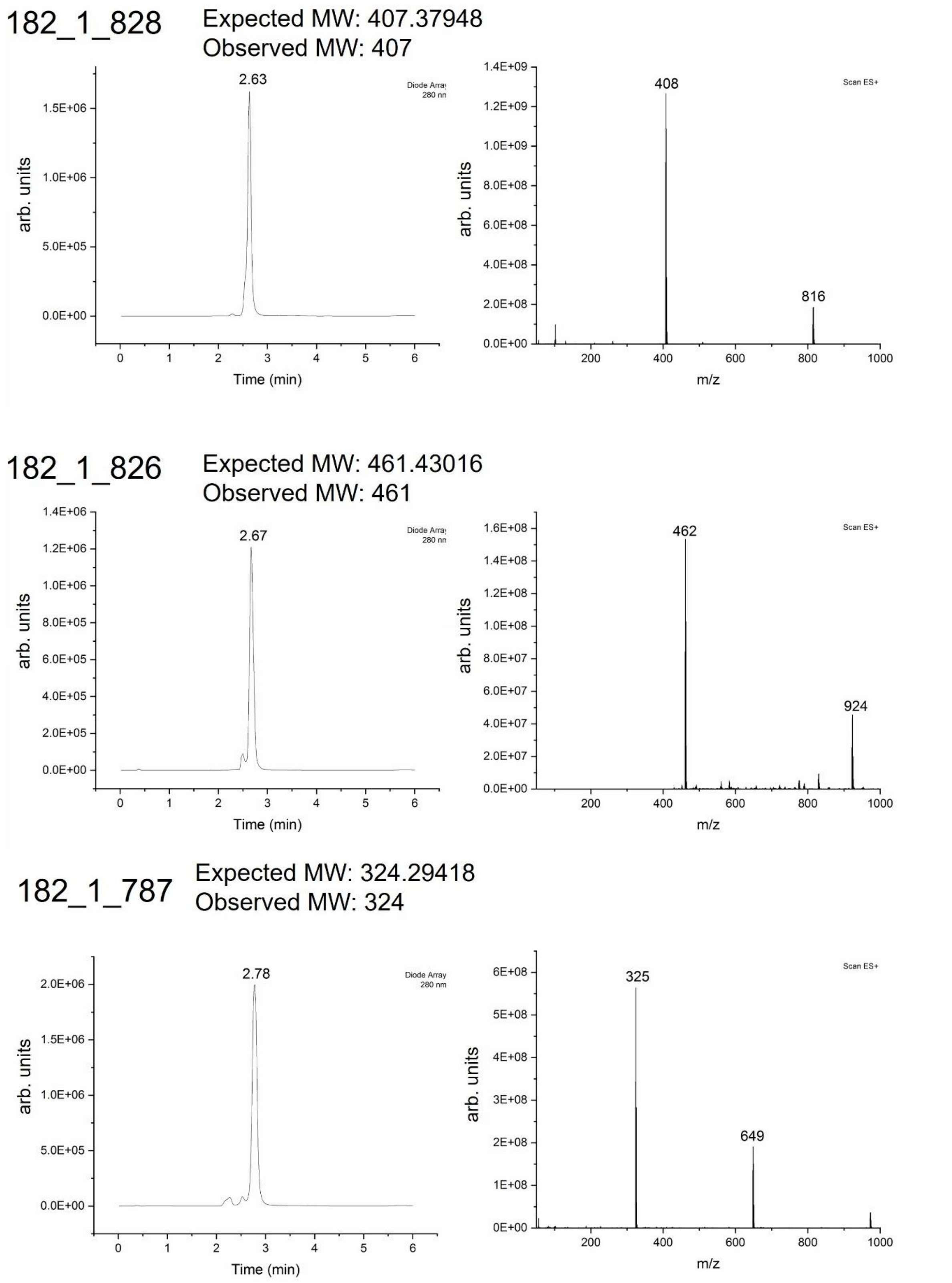

Supplementary Figure 48. UPLC and ESI-MS validation of compounds **182_1_828**, **182_1_826**, and **182_1_787**. ES: Electrospray Ionization Mode, arb. units: Arbitrary Units.

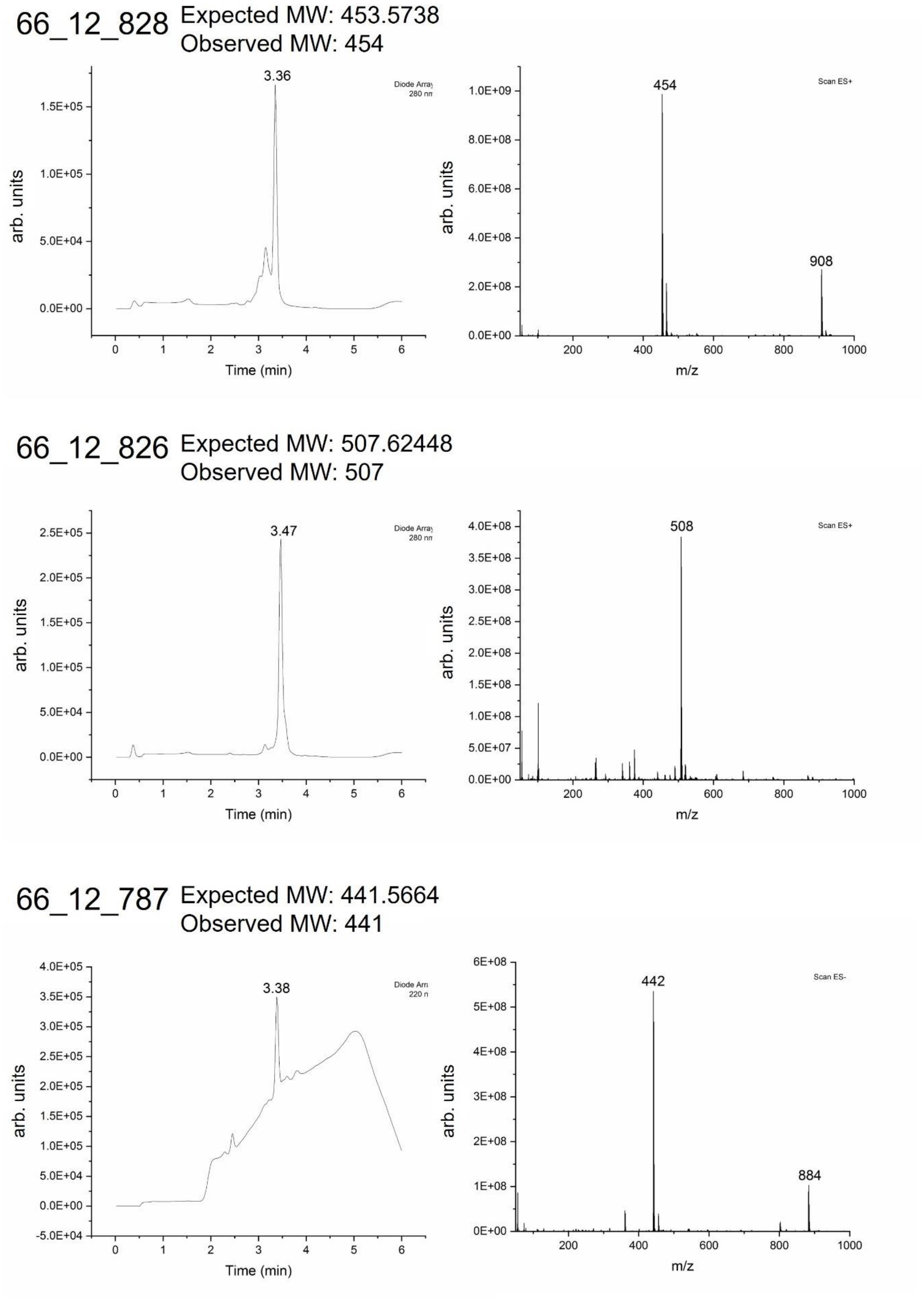

Supplementary Figure 49. UPLC and ESI-MS validation of compounds **66_12_828**, **66_12_826**, and **66_12_787**. ES: Electrospray Ionization Mode, arb. units: Arbitrary Units.

66_24_828 Expected MW: 403.53826
Observed MW: 403

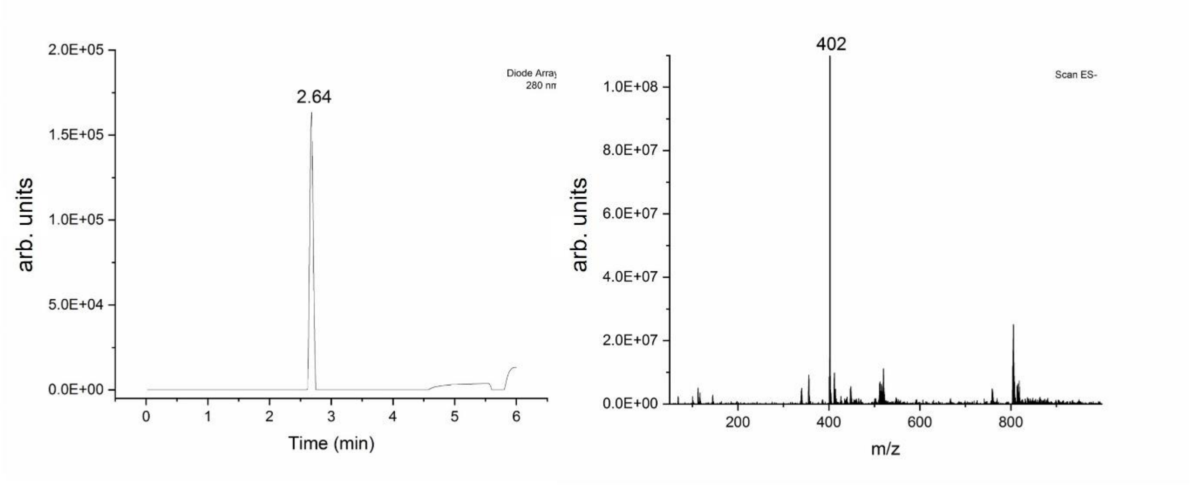

66_24_826 Expected MW: 457.58894
Observed MW: 457

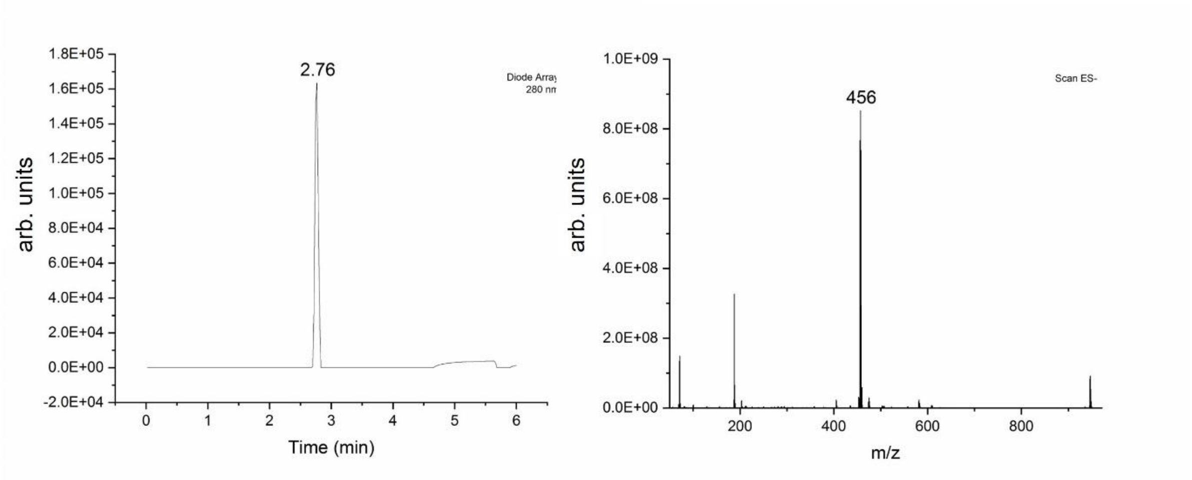

66_24_787 Expected MW: 449.57024
Observed MW: 449

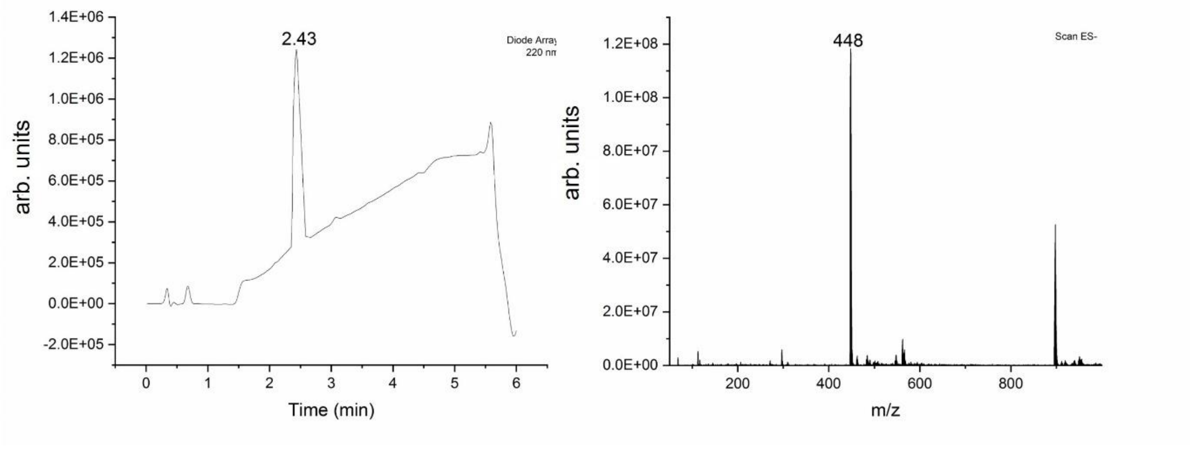

Supplementary Figure 50. UPLC and ESI-MS validation of compounds **66_24_828**, **66_24_826**, and **66_24_787**. ES: Electrospray Ionization Mode, arb. units: Arbitrary Units.

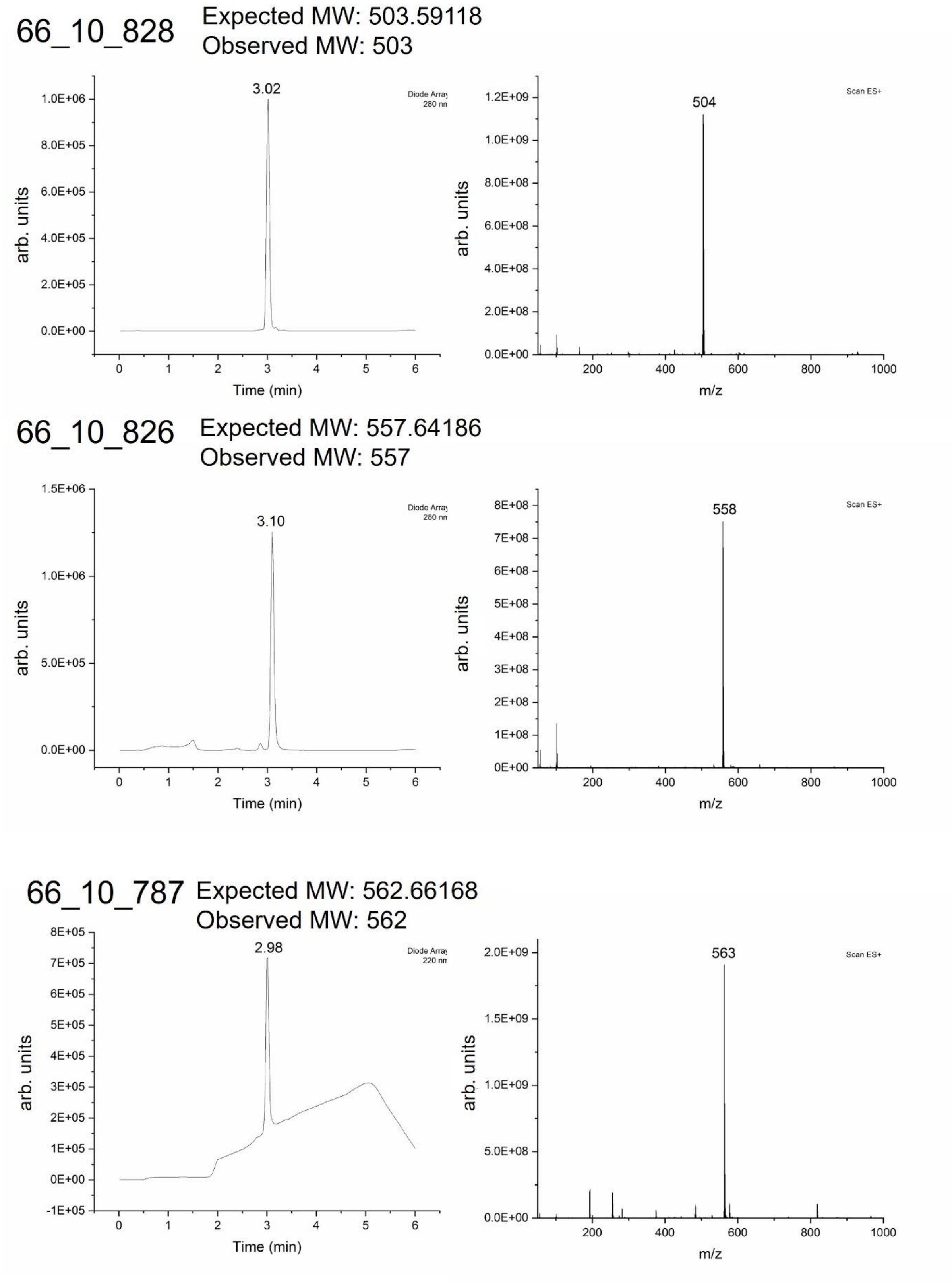

Supplementary Figure 51. UPLC and ESI-MS validation of compounds **66_10_828**, **66_10_826**, and **66_10_787**. ES: Electrospray Ionization Mode, arb. units: Arbitrary Units.

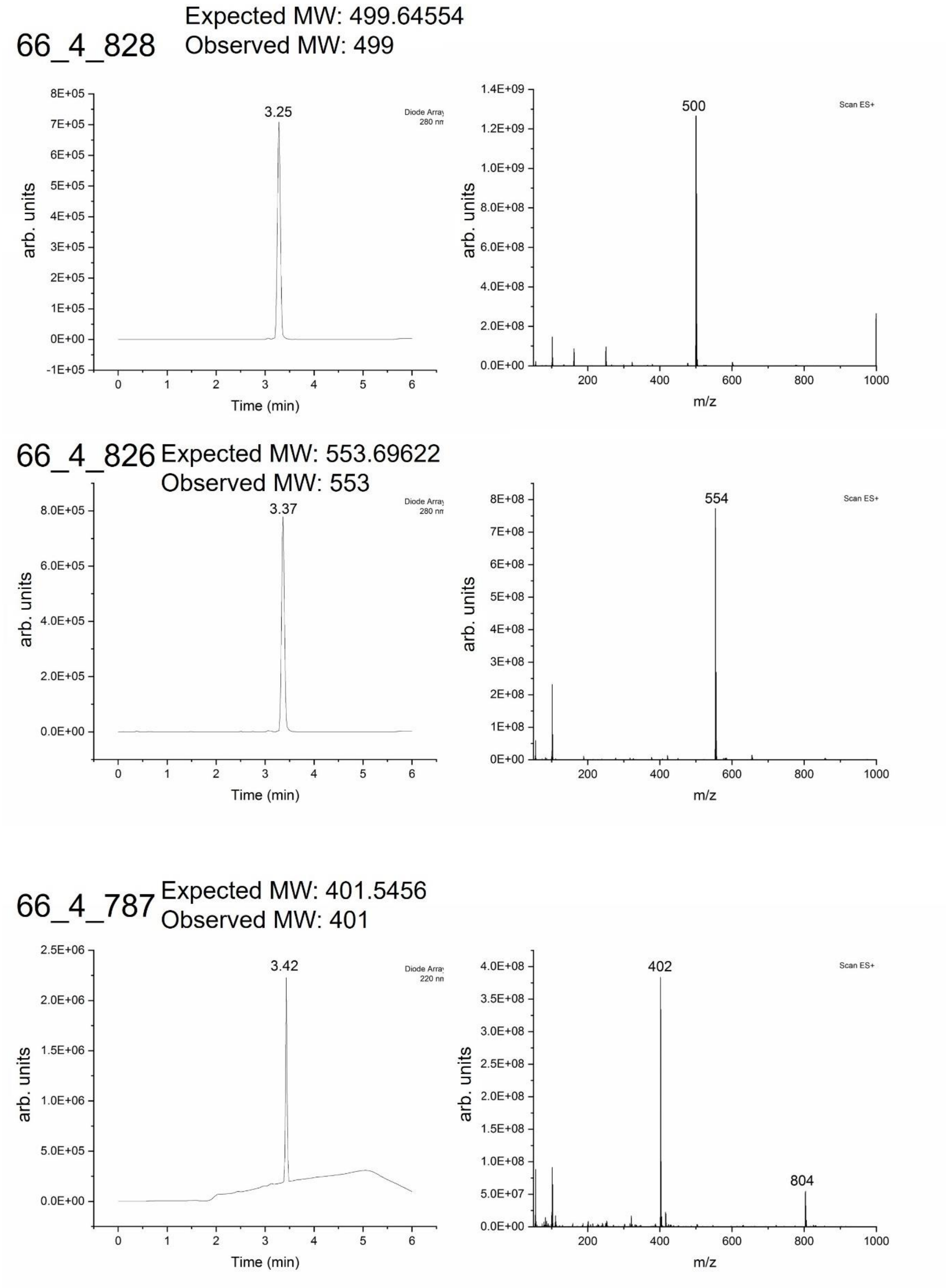

Supplementary Figure 52. UPLC and ESI-MS validation of compounds **66_4_828**, **66_4_826**, and **66_4_787**. ES: Electrospray Ionization Mode, arb. units: Arbitrary Units

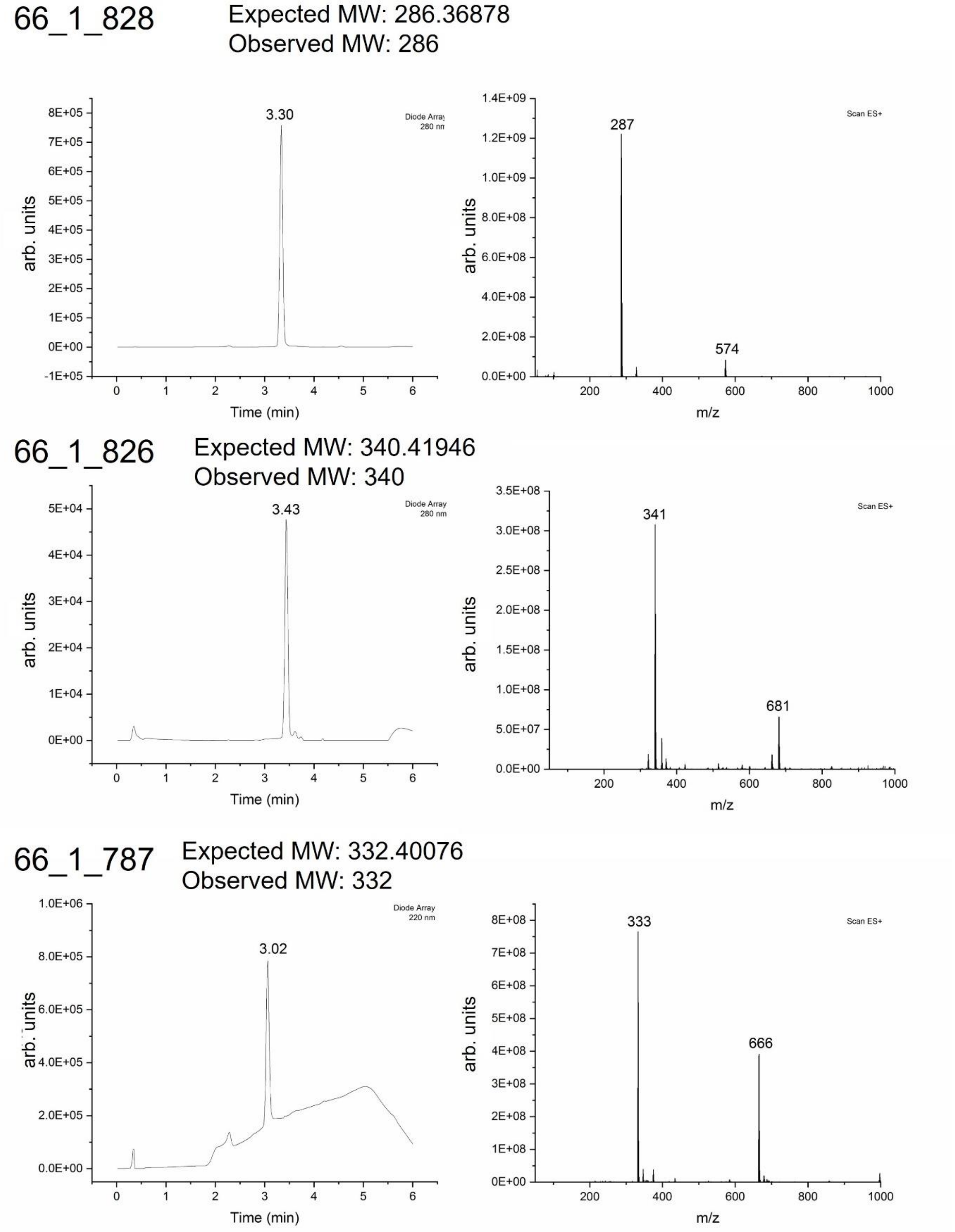

Supplementary Figure 53. UPLC and ESI-MS validation of compounds **66_1_828**, **66_1_826**, and **66_1_787**. ES: Electrospray Ionization Mode, arb. units: Arbitrary Units.

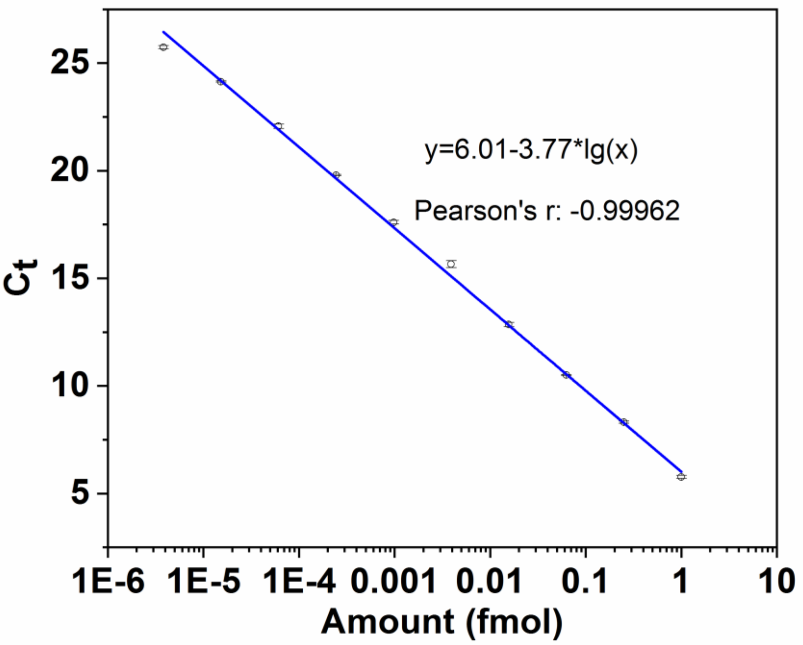

Supplementary Figure 54. The standard curve showing the correlation between the amount of the template and Ct value measured by qPCR.

Supplementary Note 3

DNA sequences used in constructing the trio-pharmacophore library

Table S1 Oligonucleotides for conjugating building blocks

	Sequence (5' to 3')	modification
5' holder for DTS	GGA GGT TGG GAT CGC AGC TCG TTT GAC TGT CAG	5' C6 amino
3' holder for DTS	CTG ACA GTC AAA TAC TCT GTC GTC TAC ACC TCC	3' C7 amino
3' holder for DTS	CTG ACA GTC AAA TAC TCT GTC GTC TAC ACC TCC	3' C7 thiol
5' holder for sub-library A	GGA GGT TGG GAT CGC AGC AGG TAC GTG	5' C6 amino
3' holder for sub-library C	CTC GAT CTG GCT GCG ATC CCA ACC TCC	3' C7 amino

Table S2 Oligonucleotides for encoding sub-library B members

	Sequence (5’ to 3’)	Modification
Code 1	AGCACCATCTCGATGAGACTCTCTGCGTCCGACGCTCTTCCGATCTC	5’ phosphorylation
Code 2	AGCACCATCCGACGTCGATCGCAGACATCCGACGCTCTTCCGATCTC	5’ phosphorylation
Code 3	AGCACCATCAGCGAGCTACGATGCAGTCACGACGCTCTTCCGATCTC	5’ phosphorylation
Code 4	AGCACCATCTATCGCGATCGATGCAGACGCGACGCTCTTCCGATCTC	5’ phosphorylation
Code 5	AGCACCATCCGCGTATATCGCGACACGATCGACGCTCTTCCGATCTC	5’ phosphorylation
Code 6	AGCACCATCGAGTGTCTCGATACACATCGCGACGCTCTTCCGATCTC	5’ phosphorylation
Code 7	AGCACCATCAGATGCTATGCGACATACACCGACGCTCTTCCGATCTC	5’ phosphorylation
Code 8	AGCACCATCATACGCTCGTCTCGTCTCGTCGACGCTCTTCCGATCTC	5’ phosphorylation
Code 9	AGCACCATCGCTGACGCGAGATGCGTGATCGACGCTCTTCCGATCTC	5’ phosphorylation
Code 10	AGCACCATCCTCACATCGAGCTCATATCACGACGCTCTTCCGATCTC	5’ phosphorylation
Code 11	AGCACCATCAGCACATAGTAGCGTGCATCCGACGCTCTTCCGATCTC	5’ phosphorylation
Code 12	AGCACCATCGCTCGCGCTGCTATACTGTCCGACGCTCTTCCGATCTC	5’ phosphorylation
Code 13	AGCACCATCCGATCATATCGAGTGTGATGCGACGCTCTTCCGATCTC	5’ phosphorylation
Code 14	AGCACCATCATCACTATCGATACTGCGATCGACGCTCTTCCGATCTC	5’ phosphorylation
Code 15	AGCACCATCTAGTGTCTACGATGTCACACCGACGCTCTTCCGATCTC	5’ phosphorylation
Code 16	AGCACCATCATCGAGAGCGAGCGTCTCGACGACGCTCTTCCGATCTC	5’ phosphorylation
Code 17	AGCACCATCTCGACTCGCATATCACTCGTCGACGCTCTTCCGATCTC	5’ phosphorylation
Code 18	AGCACCATCATATCTATATAGATGTGTCGCGACGCTCTTCCGATCTC	5’ phosphorylation
Code 19	AGCACCATCATCATAGATCGATCACTCATCGACGCTCTTCCGATCTC	5’ phosphorylation
Code 20	AGCACCATCCTCGTGATCATCTCTCATGTCGACGCTCTTCCGATCTC	5’ phosphorylation
Code 21	AGCACCATCGATCAGAGCATAGTACATCACGACGCTCTTCCGATCTC	5’ phosphorylation
Code 22	AGCACCATCCTCACTAGACGAGACGCACGCGACGCTCTTCCGATCTC	5’ phosphorylation
Code 23	AGCACCATCATATGCGCGATAGACACGTCCGACGCTCTTCCGATCTC	5’ phosphorylation
Code 24	AGCACCATCGCGACTCTATCGACTCTGACCGACGCTCTTCCGATCTC	5’ phosphorylation
Code 25	AGCACCATCTCGACTAGCGCGCAGTATCGCGACGCTCTTCCGATCTC	5’ phosphorylation
Code 26	AGCACCATCATCATGCGATCGCGCACGATCGACGCTCTTCCGATCTC	5’ phosphorylation
Code 27	AGCACCATCCTATGTAGCAGCTGCAGATGCGACGCTCTTCCGATCTC	5’ phosphorylation
Code 28	AGCACCATCCGATCTCGCGATCGCGTCACCGACGCTCTTCCGATCTC	5’ phosphorylation
Code 29	AGCACCATCCGATCTCGCGATCGCGTCACCGACGCTCTTCCGATCTC	5’ phosphorylation
Code 30	AGCACCATCCGATCTCGCGATCGCGTCACCGACGCTCTTCCGATCTC	5’ phosphorylation

Table S3. Code-specific primers for detecting individual sub-library B members

	Sequence (5' to 3')	Modification
Code 1	CGACGTACTGATATCGCTCA	none
Code 2	GTCAGTGAGATCTCGCACTAG	none
Code 3	GACGCAGAGAGTCTCATCGA	none
Code 4	TCGGATGTCTGCGATCGACG	none
Code 5	GTCGTGACTGCATCGTAGCT	none
Code 6	GTCGCGTCTGCATCGATC	none
Code 7	CGTGTCGCGATATACGCGG	none
Code 8	CGATGTGTATCGAGACACTC	none
Code 9	GTGTATGTCGCATAGCATCTG	none
Code 10	ACGAGACGAGACGAGCGTAT	none
Code 11	ATCACGCATCTCGCGTCAGC	none
Code 12	TGATATGAGCTCGATGTGAG	none
Code 13	GCGTCGGATGCACGCTACTA	none
Code 14	GTCGGACAGTATAGCAGCG	none
Code 15	ATCACACTCGATATGATCGGA	none
Code 16	GAGCGTCGATCGCAGTATC	none
Code 17	GTGTGACATCGTAGACACTA	none
Code 18	TCGAGACGCTCGCTCTCGAT	none
Code 19	GTCGACGAGTGATATGCGAG	none
Code 20	GCGTCGCGACACATCTATA	none
Code 21	TCGATGAGTGATCGATCTATGAT	none
Code 22	ACATGAGAGATGATCACGAG	none
Code 23	GTGATGTACTATGCTCTGATC	none
Code 24	CGTGCGTCTCGTCTAGTGAG	none
Code 25	ACGTGTCTATCGCGCATATGAT	none
Code 26	GCGTCGGTCAGAGTCGATA	none
Code 27	CGATACTGCGCGCTAGTCGA	none
Code 28	ATCGTGCGCGATCGCATGAT	none
Code 29	CATCTGCAGCTGCTACATAG	none
Code 30	AGCGTCGGTGACGCGATC	none

Note: The sequences listed in the table are reverse primers of the template. The forward primer is constant in all members binding to the constant region of the members with the sequence of 5' GGTTGGGATCGCAGCTCG 3'.

Supplementary Note 4

NGS data decoding was performed in two steps.1. Decoding 2. Assigning. In the decoding step, the raw NGS data were stripped to generate individual code sequence and its count. Then each code sequence was assigned to the building block ID by looking up the code list of a DNA-encoded library. The following are the scripts for decoding and assigning.

1. Decoding

```
from collections import defaultdict
import sys
import re

counter = defaultdict(int) # dictionary to count compounds

file_name = input('Enter file name of sequencing data:')

i=0
for line in open(file_name):
	line=line.strip()

	if line[:12]=='' and line[89:101]=='': line = line[:12]+"\t"+line[30:55]

	else: line = "N"+"\t"+"N"

	i+=1
	if i%4 == 2: # line number i is 4*n+2
		if all(c in "ACGTN\t" for c in line):
			counter[line]+=1
		else:
			print("Problem in line %d. Expected DNA sequence, but
found:\n%s"%(i,line))
			sys.exit(1)
f= open(" ", "w+")

print("Frequency, DNA sequence")
for (k,v) in sorted(counter.items(), key=lambda kv: kv[1], reverse=True):
	f.write("%d\t %s"%(v,k))
	f.write ("\n")
	print("%d\t %s"%(v,k))

f.close()
```

2. Assignment

```
from collections import defaultdict
import sys
import re
import csv
import codecs

code_list_name = input('Enter file name of code list:')
file_name = input('Enter output file name from the decoding program:')

# load the code to number association to dictionary
dictionarycodelist = defaultdict(int)

csv_reader = csv.reader(codecs.open(code_list_name, 'rU', 'utf-8'), delimiter=';')
for line_entries in csv_reader:
    dictionarycodelist[line_entries[0]] = line_entries[1]

# create a output file handle
fq_extension = '.fq'
output_file_name = 'out_' + file_name[:len(file_name)-len(fq_extension)]+'.csv'

output_file_handle = codecs.open(output_file_name, 'w+', 'utf-8')

# read sequencing file and replace sequences
csv_reader = csv.reader(codecs.open(file_name, 'rU', 'utf-8'), delimiter='\t')
for line_entries in csv_reader:
    sequence_replacement = dictionarycodelist[line_entries[2]]
    entry_length = len(line_entries)
    sequence_replacement2 =0
    if entry_length >3:
        sequence_replacement2 = dictionarycodelist[line_entries[3]]
        if sequence_replacement != 0 and sequence_replacement2 != 0:
            new_line = line_entries[0] + ';' + sequence_replacement + ';' +
sequence_replacement2 + '\n'
            output_file_handle.write(new_line)

    elif sequence_replacement != 0:
        new_line = line_entries[0] + ';' + sequence_replacement + '\n'
        output_file_handle.write(new_line)

output_file_handle.close()
```

Supplementary References

1. Kanan, M. W., Rozenman, M. M., Sakurai, K., Snyder, T. M. & Liu, D. R. Reaction discovery enabled by DNA-templated synthesis and in vitro selection. *Nature* **431**, 545–549 (2004).
2. Momiyama, N., Kanan, M. W. & Liu, D. R. Synthesis of acyclic α,β-unsaturated ketones via Pd(II)-catalyzed intermolecular reaction of alkynamides and alkenes. *J Am Chem Soc* **129**, 2230–2231 (2007).
3. Wichert, M. *et al.* Dual-display of small molecules enables the discovery of ligand pairs and facilitates affinity maturation. *Nat Chem* **7**, 241–249 (2015).
4. Bigatti, M. *et al.* Impact of a Central Scaffold on the Binding Affinity of Fragment Pairs Isolated from DNA-Encoded Self-Assembling Chemical Libraries. *ChemMedChem* **12**, 1748–1752 (2017).
5. Fabre, B., Ramos, A. & De Pascual-Teresa, B. Targeting matrix metalloproteinases: Exploring the dynamics of the S1′ pocket in the design of selective, small molecule inhibitors. *J Med Chem* **57**, 10205–10219 (2014).
6. Rowsell, S. *et al.* Crystal structure of human MMP9 in complex with a reverse hydroxamate inhibitor. *J Mol Biol* **319**, 173–181 (2002).
7. Hashimoto, H. *et al.* Structural basis for matrix metalloproteinase-2 (MMP-2)-selective inhibitory action of β-amyloid precursor protein-derived inhibitor. *Journal of Biological Chemistry* **286**, 33236–33243 (2011).
8. Tochowicz, A. *et al.* Crystal Structures of MMP-9 Complexes with Five Inhibitors: Contribution of the Flexible Arg424 Side-chain to Selectivity. *J Mol Biol* **371**, 989–1006 (2007).
